# Supplementary figures and images for: Man vs. machine: Multi-country experimental evidence on the quality and perceptions of AI-generated research blog content
Source: PLoS One. 2026 Mar 25;21(3):e0342852. doi: 10.1371/journal.pone.0342852 (PMC13016282; doi:10.1371/journal.pone.0342852)

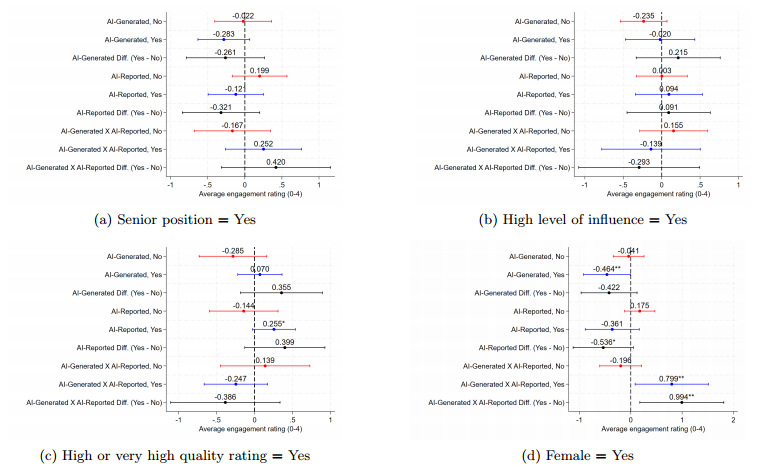

Supplement: S1 Fig — Notes: * p < 0.1, ** p < 0.05, *** p < 0.01. This figure reports on the respondents’ reported likelihood of engagement across four dimensions of heterogeneity. Engagement is measured as the mean of five actions: whether they are likely to re-read the blog, share the blog with others, look up studies cited in the blog, look up relate studies, or contact the authors. The responses follow a Likert scale with 0 indicating that they are ‘very unlikely’ and 4 indicating they are ‘very likely’ to take that action. Panel a) is whether the respondent holds a senior position, Panel b) is whether the respondent holds a high degree of influence in policy-making, Panel c) is whether the respondent gave the blog a ‘high’ or ‘very high’ quality rating, and Panel d) is whether the respondent is female. We control for respondent characteristics, wave, and strata (country, seniority, and gender) fixed effects. Standard errors are clustered at the individual level (the unit of randomization). (TIFF) [file pone.0342852.s001.tiff]

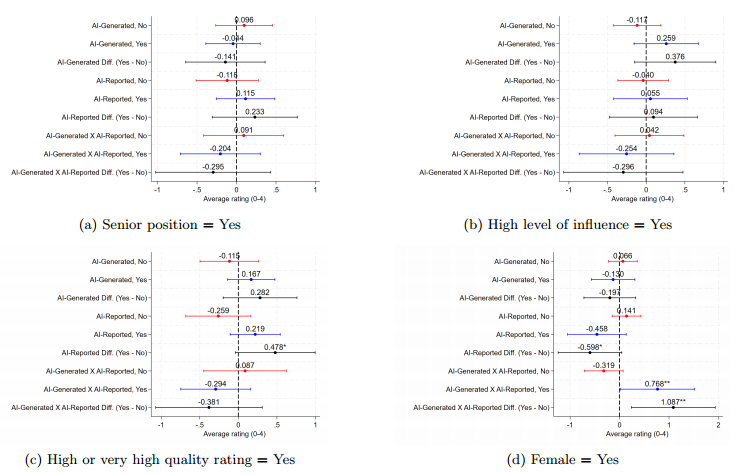

Supplement: S2 Fig — Notes: * p < 0.1, ** p < 0.05, *** p < 0.01. This figure reports on the respondents’ reported beliefs about others’ likelihood of engagement across four dimensions of heterogeneity. Engagement is measured as the mean of five actions: whether others are likely to re-read the blog, share the blog with others, look up studies cited in the blog, look up relate studies, or contact the authors. The responses follow a Likert scale with 0 indicating that others are ‘very unlikely’ and 4 indicating they are ‘very likely’ to take that action. Panel a) is whether the respondent holds a senior position, Panel b) is whether the respondent holds a high degree of influence in policy-making, Panel c) is whether the respondent gave the blog a ‘high’ or ‘very high’ quality rating, and Panel d) is whether the respondent is female. We control for respondent characteristics, wave, and strata (country, seniority, and gender) fixed effects. Standard errors are clustered at the individual level (the unit of randomization). (PDF) [file pone.0342852.s002.tiff]
