# Supplementary material for: Man vs. machine: Multi-country experimental evidence on the quality and perceptions of AI-generated research blog content
Source: PLoS One. 2026 Mar 25;21(3):e0342852. doi: 10.1371/journal.pone.0342852 (PMC13016282; doi:10.1371/journal.pone.0342852)
Supplement: S1 File — (PDF) [file pone.0342852.s003.pdf]

Appendix

A Additional Tables

Table S1. Balance

	(1)	(2)	(3)	(4)		(1)-(2)	(1)-(3)	(1)-(4)	(2)-(3)	(2)-(4)	(3)-(4)
Variable		Mean / (sd)			(p-value)			Mean Difference			
Age	43.131	40.894	41.722	43.224	0.702	2.237	1.409	-0.094	-0.829	-2.331	-1.502
	(1.628)	(1.269)	(1.464)	(1.389)	0.552						
Female	0.298	0.287	0.233	0.265	0.343	0.010	0.064	0.032	0.054	0.022	-0.032
	(0.050)	(0.047)	(0.045)	(0.045)	0.794						
Master's	0.440	0.447	0.467	0.429	0.067	-0.006	-0.026	0.012	-0.020	0.018	0.038
	(0.054)	(0.052)	(0.053)	(0.050)	0.977						
PhD	0.321	0.351	0.344	0.418	0.686	-0.030	-0.023	-0.097	0.007	-0.067	-0.074
	(0.051)	(0.049)	(0.050)	(0.050)	0.561						
Seniority: Mid-level	0.262	0.255	0.289	0.214	0.413	0.007	-0.027	0.048	-0.034	0.041	0.075
	(0.048)	(0.045)	(0.048)	(0.042)	0.744						
Seniority: Senior-level	0.512	0.500	0.500	0.531	0.067	0.012	0.012	-0.019	0.000	-0.031	-0.031
	(0.055)	(0.052)	(0.053)	(0.051)	0.977						
NGO	0.202	0.298	0.200	0.163	1.655	-0.095	0.002	0.039	0.098*	0.135**	0.037
	(0.044)	(0.047)	(0.042)	(0.038)	0.176						
Research Institute	0.452	0.383	0.400	0.408	0.264	0.069	0.052	0.044	-0.017	-0.025	-0.008
	(0.055)	(0.050)	(0.052)	(0.050)	0.851						
Students and temporary workers	0.060	0.021	0.067	0.092	2.112*	0.038	-0.007	-0.032	-0.045	-0.071**	-0.025
	(0.026)	(0.015)	(0.026)	(0.029)	0.098						
Private sector	0.155	0.096	0.056	0.122	1.913	0.059	0.099**	0.032	0.040	-0.027	-0.067*
	(0.040)	(0.031)	(0.024)	(0.033)	0.127						
Agriculture	0.607	0.596	0.567	0.520	0.583	0.011	0.040	0.087	0.029	0.075	0.046
	(0.054)	(0.051)	(0.053)	(0.051)	0.626						
Economics, finance & business	0.321	0.255	0.311	0.265	0.589	0.066	0.010	0.056	-0.056	-0.010	0.046
	(0.051)	(0.045)	(0.049)	(0.045)	0.623						
Human capital (education, health)	0.679	0.734	0.656	0.755	0.903	-0.055	0.023	-0.077	0.078	-0.021	-0.100
	(0.051)	(0.046)	(0.050)	(0.044)	0.440						
Environment	0.238	0.298	0.200	0.327	1.629	-0.060	0.038	-0.088	0.098	-0.029	-0.127*
	(0.047)	(0.047)	(0.042)	(0.048)	0.182						
Other field	0.095	0.191	0.133	0.133	1.120	-0.096*	-0.038	-0.037	0.058	0.059	0.001
	(0.032)	(0.041)	(0.036)	(0.034)	0.341						
Policy influence 'high' or 'very high'	0.357	0.340	0.344	0.357	0.025	0.017	0.013	0.000	-0.004	-0.017	-0.013
	(0.053)	(0.049)	(0.050)	(0.049)	0.995						
p-value of joint significance						0.175	0.284	0.195	0.154	0.187	0.274
Number of observations	84	94	90	98	366	178	174	182	184	192	188

Notes: * p<0.1, ** p<0.05, *** p<0.01.This table reports on the results of the balance tests for the key study indicators. Columns 1 to 4 show the means and standard errors for the 4 groups where 1 represents Human-Human, 2 represents Human-AI, 3 represents AI-AI and 4 AI-Human. Columns 6 to 11 report on the pairwise t-tests between the groups.

Table S2. Summary Statistics

	Mean	Standard deviation
**Perceived quality of blog:**		
Unweighted average quality (0-4)	2.874	0.655
Weighted average quality	2.903	0.657
Number of 'agree' and 'strongly agree' statements (0-1)	9.306	3.372
Overall quality of brief rated 'high' or 'very high'	0.601	0.490
**Intended engagement with blog:**		
Average engagement rating (0-4)	2.647	0.840
Number of 'likely' and 'very likely' statements (0-5)	3.748	1.973
**Others' intended engagement with blog:**		
Average rating (0-4)	2.55	0.815
Number of 'likely' or 'very likely' statements (0-5)	3.473	2.160
Others' overall rating	0.593	0.492
**Preferences of policy blogs characteristics (0-4)**:		
Relevance to my work	3.500	0.669
Wide range of views	3.306	0.796
Level of detail provided	3.336	0.800
Title of the policy blog sparks my curiosity	3.464	0.745
Rationale presented is convincing	3.483	0.656
Visual presentation is attractive	3.188	0.837
Length of the policy blog is adequate	3.133	0.798
Content is easy to understand	3.612	0.616
Proposed recommendations are clear	3.661	0.628
Preference on number of pages	7.915	13.113
Preference on number of figures	6.581	7.026
Trust AI to write high-quality blogs	0.604	0.489
Aware of AI tools	0.809	0.394
Used AI tools conditional on awareness (N=296)	0.757	0.429
N		366

Table S3. Perceived Quality of the Blog, with Controls

	(1)	(2)	(3)	(4)	(5)	(6)
	Unweighted Average Quality (0-4) (10 Attributes)	Weighted Average Quality (0-4) (9 Attributes)	Unweighted average quality (0-4) (9 Attributes)	Standardized First Principal Component (10 Attributes)	Number of 'agree' or 'strongly agree' statements (0-10)	Overall quality of brief rated 'high' or 'very high'
(1) AI-Generated	-0.171*	-0.154	-0.158	-0.225	-0.939**	-0.156**
	(0.098)	(0.099)	(0.099)	(0.144)	(0.422)	(0.074)
RI p-value	[.095]	[.176]	[.132]	[.118]	[.038]	[.03]
(2) AI-Reported	-0.070	-0.065	-0.078	-0.099	-0.474	-0.113
	(0.099)	(0.102)	(0.102)	(0.146)	(0.447)	(0.076)
RI p-value	[.455]	[.529]	[.438]	[.491]	[.261]	[.14]
(3) AI-Generated × AI-Reported	0.247*	0.242*	0.256*	0.322	1.238**	0.226**
	(0.138)	(0.142)	(0.142)	(0.203)	(0.618)	(0.110)
RI p-value (Permuting AI-Generated)	[.091]	[.13]	[.088]	[.147]	[.053]	[.035]
RI p-value (Permuting AI-Reported)	[.104]	[.1]	[.086]	[.135]	[.041]	[.04]
Mean of Human-Generated and Human-Reported	2.954	2.943	2.939	.141	7.75	.702
(1) + (3): Total Effect of AI-Generated	0.076	0.088	0.098	0.097	0.299	0.070
(1) + (2) + (3): Total Effect of AI	0.006	0.023	0.020	-0.001	-0.176	-0.043
Observations	366	366	366	366	366	366

Notes: * p<0.1, ** p<0.05, *** p<0.01. This table reports the effects of the treatments on perceptions of the blog's quality measured using 10 attributes of high quality blogs (Cash2003, Balian2016, Nagyova2023 and Fillol2022a). Perceptions are measured using a 5-point Likert scale ranging from 'strongly disagree' that the blog is high-quality with respect to this attribute (0) to 'strongly agree' (4). The 10 attributes are: blog had an appropriate amount of detail, a clear rationale, a catchy title, good visual presentation, an appropriate tone, was of adequate length, was easy to understand, and provided clear, relevant, and sufficiently detailed recommendations. The outcome in the first column is the unweighted average across the 10 attributes, and the outcome in the second column is the weighted average across nine attributes (weighted by respondents' reported relative importance of these attributes save appropriate tone). The outcome in the third column is the unweighted average of the nine attributes that are included in the weighted score, as a robustness check. The outcome in the fourth column is the standardized score of the first principal component, calculated based on the same 10 attributes using Polychoric PCA. The outcome in the fifth column is the total number of the 10 attributes rated as either 'agree' or 'highly agree' while 'neutral', 'disagree', and 'strongly disagree' are assigned a value of 0. The outcome in the sixth column is an indicator variable equal to 1 if the overall quality of the brief is rated either 'high' or 'very high' from a separate survey question, while 'average', 'low', and 'very low' are coded as 0. We control for respondent characteristics, wave, and strata (country, seniority, and gender) fixed effects. Standard errors are clustered at the individual level (the unit of randomization).

Table S4. Perceived Quality of the Blog, without Controls

	(1)	(2)	(3)	(4)	(5)	(6)
	Unweighted Average Quality (0-4) (10 Attributes)	Weighted Average Quality (0-4) (9 Attributes)	Unweighted average quality (0-4) (10 Attributes)	Standardized First Principal Component (10 Attributes)	Number of 'agree' or 'strongly agree' statements (0-10)	Overall quality of brief rated 'high' or 'very high'
(1) AI-Generated	-0.189*	-0.174*	-0.179*	-0.249*	-0.922**	-0.164**
	(0.100)	(0.103)	(0.103)	(0.146)	(0.431)	(0.071)
RI p-value	[.071]	[.088]	[.079]	[.088]	[.036]	[.024]
(2) AI-Reported	-0.070	-0.063	-0.077	-0.090	-0.453	-0.104
	(0.098)	(0.100)	(0.101)	(0.145)	(0.433)	(0.075)
RI p-value	[.441]	[.536]	[.435]	[.551]	[.287]	[.161]
(3) AI-Generated × AI-Reported	0.224	0.214	0.231	0.277	1.058*	0.212**
	(0.139)	(0.144)	(0.144)	(0.205)	(0.606)	(0.105)
RI p-value (Permuting AI-Generated)	[.126]	[.145]	[.124]	[.167]	[.091]	[.052]
RI p-value (Permuting AI-Reported)	[.106]	[.119]	[.111]	[.176]	[.08]	[.045]
Mean of Human-Generated and Human-Reported	2.954	2.943	2.939	.141	7.75	.702
(1) + (3): Total Effect of AI-Generated	0.035	0.040	0.051	0.029	0.136	0.048
(1) + (2) + (3): Total Effect of AI	-0.035	-0.023	-0.025	-0.061	-0.317	-0.056
Observations	366	366	366	366	366	366

Notes: * p<0.1, ** p<0.05, *** p<0.01. This table reports the effects of the treatments on perceptions of the blog's quality measured using 10 attributes of high quality blogs (Cash2003, Balian2016, Nagyova2023 and Fillol2022a). Perceptions are measured using a 5-point Likert scale ranging from 'strongly disagree' that the blog is high-quality with respect to this attribute (0) to 'strongly agree' (4). The 10 attributes are: blog had an appropriate amount of detail, a clear rationale, a catchy title, good visual presentation, an appropriate tone, was of adequate length, was easy to understand, and provided clear, relevant, and sufficiently detailed recommendations. The outcome in the first column is the unweighted average across the 10 attributes, and the outcome in the second column is the weighted average across nine attributes (weighted by respondents' reported relative importance of these attributes save appropriate tone). The outcome in the third column is the unweighted average of the nine attributes that are included in the weighted score, as a robustness check. The outcome in the fourth column is the standardized score of the first principal component, calculated based on the same 10 attributes using Polychoric PCA. The outcome in the fifth column is the total number of the 10 attributes rated as either 'agree' or 'highly agree' while 'neutral', 'disagree', and 'strongly disagree' are assigned a value of 0. The outcome in the sixth column is an indicator variable equal to 1 if the overall quality of the brief is rated either either 'high' or 'very high' from a separate survey question, while 'average', 'low', and 'very low' are coded as 0. We control for wave and strata (country, seniority, and gender) fixed effects. Standard errors are clustered at the individual level (the unit of randomization).

Table S5. Perceived Quality by Reading Grade Level, with Controls

	(1)	(2)	(3)	(4)	(5)	(6)
	Unweighted Average Quality (0-4) (10 Attributes)	Weighted Average Quality (0-4) (9 Attributes)	Unweighted average quality (0-4) (10 Attributes)	Standardized First Principal Component (10 Attributes)	Number of 'agree' or 'strongly agree' statements (0-10)	Overall quality of brief rated 'high' or 'very high'
(1) AI Generated	-1.220	-1.203	-1.248	-1.570	-7.881	0.290
	(1.229)	(1.269)	(1.262)	(1.809)	(5.502)	(0.948)
RI p-value	[.167]	[.183]	[.167]	[.215]	[.022]	[.641]
(2) AI-reported	-0.949	-1.284	-1.098	-1.067	-6.005	-0.603
	(1.402)	(1.469)	(1.447)	(2.056)	(6.555)	(1.111)
RI p-value	[.516]	[.412]	[.475]	[.625]	[.378]	[.59]
(3) AI Generated × AI-reported	1.979	2.256	2.181	2.367	9.479	0.859
	(1.512)	(1.577)	(1.562)	(2.220)	(7.018)	(1.231)
RI p-value (Permuting AI Generated)	[.102]	[.07]	[.08]	[.188]	[.056]	[.322]
RI p-value (Permuting AI-reported)	[.239]	[.205]	[.21]	[.334]	[.225]	[.501]
(4) Reading Grade Level	-0.010	-0.012	-0.014	-0.010	-0.244	0.009
	(0.070)	(0.073)	(0.072)	(0.103)	(0.330)	(0.054)
RI p-value	[.753]	[.729]	[.696]	[.831]	[.081]	[.717]
(5) AI Generated × Reading Grade Level	0.071	0.070	0.073	0.091	0.438	-0.029
	(0.074)	(0.077)	(0.076)	(0.110)	(0.328)	(0.058)
RI p-value (Permuting AI Generated)	[.194]	[.209]	[.193]	[.244]	[.044]	[.435]
RI p-value (Permuting Reading Grade Level)	[.133]	[.115]	[.104]	[.153]	[.027]	[.33]
(6) AI-reported × Reading Grade Level	0.052	0.072	0.060	0.057	0.326	0.029
	(0.082)	(0.086)	(0.085)	(0.120)	(0.383)	(0.065)
RI p-value (Permuting AI-reported)	[.547]	[.429]	[.496]	[.636]	[.412]	[.652]
RI p-value (Permuting Reading Grade Level)	[.363]	[.162]	[.274]	[.517]	[.064]	[.31]
(7) AI Generated × AI-reported × Reading Grade Level	-0.110	-0.127	-0.122	-0.131	-0.513	-0.039
	(0.091)	(0.095)	(0.094)	(0.133)	(0.417)	(0.074)
RI p-value (Permuting AI Generated)	[.142]	[.101]	[.114]	[.242]	[.089]	[.483]
RI p-value (Permuting AI-reported)	[.278]	[.241]	[.25]	[.365]	[.271]	[.616]
RI p-value (Permuting Reading Grade Level)	[.046]	[.033]	[.037]	[.094]	[.046]	[.402]
Mean of Human-Generated and Human-Reported	2.954	2.943	2.939	.141	7.75	.702
(1) + (5): Effect of AI-Generated, +1 Grade	-1.149	-1.133	-1.175	-1.479	-7.443	0.260
(2) + (6): Effect of AI-Reported, +1 Grade	-0.898	-1.212	-1.038	-1.010	-5.679	-0.575
(3) + (7): Effect of AI-Generated × AI-Reported, +1 Grade	1.868	2.129	2.059	2.236	8.967	0.820
(1) + (2) + (3): Total Effect of AI, +1 Grade	-0.190	-0.231	-0.165	-0.270	-4.407	0.545
(1) + (2) + (3) + (5) + (6) + (7): Total Effect of AI, +1 Grade	-0.178	-0.215	-0.154	-0.253	-4.155	0.506
(5) + (6) + (7): Difference in Total Effect of AI, +1 Grade	0.012	0.016	0.011	0.017	0.252	-0.039
Observations	366	366	366	366	366	366

Notes: * p<0.1, ** p<0.05, *** p<0.01. This table reports the heterogeneous effects of the treatments on perceptions of the blog's quality by the reading level of the blog. The Flesch-Kincaid reading-level measure is used to construct a continuous measure of the grade level of the blogs Flesch1948. We include the grade level of the blog as well as interact it with indicators for AI-generated blogs, AI-reported blogs, and their interaction (resulting in a triple interaction). Perceptions are measured using a 5-point Likert scale ranging from 'strongly disagree' that the blog is high-quality with respect to this attribute (0) to 'strongly agree' (4). The 10 attributes are: blog had an appropriate amount of detail, a clear rationale, a catchy title, good visual presentation, an appropriate tone, was of adequate length, was easy to understand, and provided clear, relevant, and sufficiently detailed recommendations. The outcome in the first column is the unweighted average across the 10 attributes, and the outcome in the second column is the weighted average across nine attributes (weighted by respondents' reported relative importance of these attributes save appropriate tone). The outcome in the third column is the unweighted average of the nine statements, as a robustness check. The outcome in the fourth column is the standardized score of the first principal component, calculated based on the same 10 attributes using Polychoric PCA. The outcome in the fifth column is the total number of the 10 attributes rated as either 'agree' or 'highly agree' while 'neutral', 'disagree', and 'strongly disagree' are assigned a value of 0. The outcome in the sixth column is an indicator variable equal to 1 if the overall quality of the brief is rated either 'high' or 'very high' while 'neutral', 'low', and 'very low' are assigned a value of 0. We control for respondent characteristics, wave, and strata (country, seniority, and gender) fixed effects. Standard errors are clustered at the individual level (the unit of randomization).

Table S6. Perceived Quality by Reading Grade Level, without Controls

	(1)	(2)	(3)	(4)	(5)	(6)
	Unweighted Average Quality (0-4) (10 Attributes)	Weighted Average Quality (0-4) (9 Attributes)	Unweighted average quality (0-4) (10 Attributes)	Standardized First Principal Component (10 Attributes)	Number of 'agree' and 'strongly agree' statements (0-10)	Overall quality of brief rated 'high' or 'very high'
(1) AI-Generated	-0.658	-0.545	-0.630	-0.714	-5.772	0.324
	(1.197)	(1.231)	(1.225)	(1.772)	(5.270)	(0.927)
RI p-value	[.439]	[.533]	[.476]	[.556]	[.106]	[.586]
(2) AI-Reported	-0.502	-0.746	-0.591	-0.483	-4.243	-0.565
	(1.363)	(1.415)	(1.400)	(2.009)	(6.246)	(1.139)
RI p-value	[.73]	[.63]	[.691]	[.823]	[.526]	[.607]
(3) AI-Generated × AI-Reported	1.553	1.753	1.692	1.792	7.803	0.901
	(1.502)	(1.557)	(1.548)	(2.211)	(6.798)	(1.254)
RI p-value (Permuting AI-Generated)	[.17]	[.137]	[.154]	[.283]	[.097]	[.266]
RI p-value (Permuting AI-Reported)	[.332]	[.307]	[.302]	[.45]	[.285]	[.47]
(4) Reading Grade Level	0.041	0.047	0.042	0.067	-0.033	0.023
	(0.068)	(0.071)	(0.070)	(0.101)	(0.310)	(0.052)
RI p-value	[.243]	[.202]	[.257]	[.195]	[.822]	[.325]
(5) AI-Generated × Reading Grade Level	0.039	0.034	0.038	0.044	0.330	-0.030
	(0.072)	(0.074)	(0.074)	(0.107)	(0.314)	(0.057)
RI p-value (Permuting AI-Generated)	[.458]	[.526]	[.471]	[.55]	[.12]	[.399]
RI p-value (Permuting Reading Grade Level)	[.352]	[.41]	[.354]	[.449]	[.098]	[.304]
(6) AI-Reported × Reading Grade Level	0.025	0.040	0.030	0.023	0.223	0.027
	(0.080)	(0.083)	(0.082)	(0.118)	(0.365)	(0.067)
RI p-value (Permuting AI-Reported)	[.766]	[.652]	[.73]	[.856]	[.562]	[.676]
RI p-value (Permuting Reading Grade Level)	[.703]	[.552]	[.649]	[.845]	[.275]	[.311]
(7) AI-Generated × AI-Reported × Reading Grade Level	-0.087	-0.099	-0.096	-0.101	-0.428	-0.043
	(0.090)	(0.094)	(0.093)	(0.133)	(0.406)	(0.076)
RI p-value (Permuting AI-Generated)	[.215]	[.17]	[.191]	[.335]	[.145]	[.407]
RI p-value (Permuting AI-Reported)	[.363]	[.332]	[.343]	[.491]	[.329]	[.574]
RI p-value (Permuting Reading Grade Level)	[.098]	[.065]	[.074]	[.173]	[.067]	[.315]
Mean of Human-Generated and Human-Reported	2.954	2.943	2.939	.141	7.75	.702
(1) + (5): Effect of AI-Generated, +1 Grade	-0.618	-0.512	-0.592	-0.670	-5.442	0.294
(2) + (6): Effect of AI-Reported, +1 Grade	-0.477	-0.706	-0.561	-0.460	-4.020	-0.538
(3) + (7): Effect of AI-Generated × AI-Reported, +1 Grade	1.466	1.654	1.596	1.692	7.376	0.858
(1) + (2) + (3): Total Effect of AI, +1 Grade	0.393	0.461	0.470	0.595	-2.212	0.660
(1) + (2) + (3) + (5) + (6) + (7): Total Effect of AI, +1 Grade	0.370	0.436	0.443	0.561	-2.087	0.614
(5) + (6) + (7): Difference in Total Effect of AI, +1 Grade	-0.022	-0.025	-0.027	-0.034	0.126	-0.046
Observations	366	366	366	366	366	366

Notes: * $p<0.1$, ** $p<0.05$, *** $p<0.01$. This table reports the heterogeneous effects of the treatments on perceptions of the blog's quality by the reading level of the blog. The Flesch-Kincaid reading-level measure is used to construct a continuous measure of the grade level of the blogs Flesch1948. We include the grade level of the blog as well as interact it with indicators for AI-generated blogs, AI-reported blogs, and their interaction (resulting in a triple interaction). Perceptions are measured using a 5-point Likert scale ranging from 'strongly disagree' that the blog is high-quality with respect to this attribute (0) to 'strongly agree' (4). The 10 attributes are: blog had an appropriate amount of detail, a clear rationale, a catchy title, good visual presentation, an appropriate tone, was of adequate length, was easy to understand, and provided clear, relevant, and sufficiently detailed recommendations. The outcome in the first column is the unweighted average across the 10 attributes, and the outcome in the second column is the weighted average across nine attributes (weighted by respondents' reported relative importance of these attributes save appropriate tone). The outcome in the third column is the unweighted average of the nine statements, as a robustness check. The outcome in the fourth column is the standardized score of the first principal component, calculated based on the same 10 attributes using Polychoric PCA. The outcome in the fifth column is the total number of the 10 attributes rated as either 'agree' or 'highly agree' while 'neutral', 'disagree', and 'strongly disagree' are assigned a value of 0. The outcome in the sixth column is an indicator variable equal to 1 if the overall quality of the brief is rated either 'high' or 'very high' while 'average', 'low', and 'very low' are assigned a value of 0. We control for wave and strata (country, seniority, and gender) fixed effects. Standard errors are clustered at the individual level (the unit of randomization).

Table S7. Perceived Quality by Reading Grade Level, with Controls - Disaggregated

	(1)	(2)	(3)	(4)	(5)	(6)	(7)	(8)	(9)	(10)
	Title sparked curiosity	Appropriate level of detail	Clear rationale	Visual presentation	Appropriate tone	Adequate length	Easy to understand	Clear proposed recommendations	Relevant proposed recommendations	Sufficiently detailed recommendations
(1) AI Generated	0.015	-0.692	-3.479**	-3.273	-0.968	-1.048	-0.992	-1.054	-2.124	1.417
	(1.710)	(1.741)	(1.709)	(2.071)	(1.503)	(2.204)	(1.662)	(1.573)	(1.701)	(2.068)
RI p-value	[.995]	[.602]	[.004]	[.01]	[.393]	[.453]	[.311]	[.354]	[.047]	[.311]
(2) AI-reported	-0.396	0.671	-2.556	-0.519	0.389	-1.400	-1.495	-1.545	-2.430	-0.213
	(1.794)	(2.022)	(2.052)	(2.365)	(1.613)	(2.365)	(1.783)	(2.012)	(2.139)	(2.518)
RI p-value	[.832]	[.748]	[.284]	[.851]	[.821]	[.546]	[.411]	[.45]	[.305]	[.941]
(3) AI Generated × AI-reported	1.798	1.484	3.844*	3.035	0.160	2.851	1.633	1.146	3.590	0.249
	(1.986)	(2.271)	(2.217)	(2.537)	(1.768)	(2.585)	(1.908)	(2.144)	(2.280)	(2.725)
RI p-value (Permuting AI Generated)	[.291]	[.41]	[.024]	[.141]	[.892]	[.12]	[.209]	[.466]	[.028]	[.904]
RI p-value (Permuting AI-reported)	[.365]	[.541]	[.118]	[.291]	[.923]	[.292]	[.422]	[.623]	[.141]	[.929]
(4) Reading Grade Level	0.012	0.054	-0.140	-0.061	0.018	0.081	-0.064	-0.069	-0.074	0.140
	(0.093)	(0.102)	(0.099)	(0.121)	(0.084)	(0.120)	(0.095)	(0.091)	(0.099)	(0.120)
RI p-value	[.818]	[.301]	[0]	[.285]	[.609]	[.079]	[.085]	[.101]	[.1]	[.027]
(5) AI Generated × Reading Grade Level	-0.014	0.038	0.201*	0.191	0.050	0.067	0.051	0.063	0.143	-0.082
	(0.104)	(0.104)	(0.103)	(0.123)	(0.090)	(0.136)	(0.101)	(0.095)	(0.103)	(0.123)
RI p-value (Permuting AI Generated)	[.868]	[.651]	[.009]	[.015]	[.462]	[.454]	[.4]	[.36]	[.036]	[.339]
RI p-value (Permuting Reading Grade Level	[.764]	[.607]	[.005]	[.057]	[.463]	[.298]	[.199]	[.19]	[.02]	[.25]
(6) AI-reported × Reading Grade Level	0.020	-0.049	0.155	0.024	-0.023	0.075	0.084	0.088	0.143	0.003
	(0.105)	(0.117)	(0.120)	(0.138)	(0.094)	(0.141)	(0.104)	(0.117)	(0.125)	(0.146)
RI p-value (Permuting AI-reported)	[.856]	[.689]	[.271]	[.879]	[.815]	[.592]	[.418]	[.442]	[.305]	[.992]
RI p-value (Permuting Reading Grade Level)	[.741]	[.552]	[.002]	[.8]	[.73]	[.418]	[.047]	[.101]	[.011]	[.99]
(7) AI Generated × AI-reported × Reading Grade Level	-0.093	-0.091	-0.220*	-0.174	-0.004	-0.163	-0.089	-0.056	-0.222	0.008
	(0.121)	(0.136)	(0.132)	(0.151)	(0.105)	(0.158)	(0.114)	(0.127)	(0.137)	(0.162)
RI p-value (Permuting AI Generated)	[.385]	[.41]	[.032]	[.167]	[.968]	[.162]	[.281]	[.553]	[.034]	[.956]
RI p-value (Permuting AI-reported)	[.452]	[.524]	[.142]	[.313]	[.97]	[.332]	[.465]	[.694]	[.129]	[.962]
RI p-value (Permuting Reading Grade Level)	[.281]	[.411]	[.023]	[.164]	[.954]	[.027]	[.111]	[.411]	[.013]	[.96]
Mean of Human-Generated and Human-Reported	3.107	2.929	2.893	2.583	3.083	3.071	3.25	3.012	2.94	2.667
(1) + (5): Effect of AI-Generated, +1 Grade	0.000	-0.654	-3.278**	-3.082	-0.917	-0.981	-0.941	-0.990	-1.981	1.335
(2) + (6): Effect of AI-Reported, +1 Grade	-0.376	0.622	-2.402	-0.495	0.366	-1.325	-1.411	-1.456	-2.288	-0.210
(3) + (7): Effect of AI-Generated × AI-Reported, +1 Grade	1.705	1.393	3.624*	2.861	0.156	2.688	1.543	1.090	3.367	0.257
(1) + (2) + (3): Total Effect of AI, +1 Grade	1.417	1.462	-2.191	-0.757	-0.419	0.403	-0.854	-1.452	-0.964	1.454
(1) + (2) + (3) + (5) + (6) + (7): Total Effect of AI, +1 Grade	1.329	1.360	-2.056	-0.717	-0.395	0.382	-0.809	-1.356	-0.901	1.382
(5) + (6) + (7): Difference in Total Effect of AI, +1 Grade	-0.087	-0.102	0.135	0.040	0.024	-0.022	0.045	0.096	0.063	-0.072
Observations	366	366	366	366	366	366	366	366	366	366

Notes: * p<0.1, ** p<0.05, *** p<0.01. This table reports the heterogeneous effects of the treatments on perceptions of the blog's quality by the reading level of the blog for each of the 10 quality attributes separately. The Flesch-Kincaid reading-level measure is used to construct a continuous measure of the grade level of the blogs Flesch1948. We include the grade level of the blog and interact it with indicators for AI-generated blogs, AI-reported blogs, and their interaction (resulting in a triple interaction). Perceptions are measured using a 5-point Likert scale ranging from 'strongly disagree' that the blog is high-quality with respect to this attribute (0) to 'strongly agree' (4). An indicator variable is assigned a value of 1 if the response is either 'agree' or 'strongly agree' while 'neutral', 'disagree', and 'strongly disagree' are assigned a value of 0. We control for respondent characteristics, wave, and strata (country, seniority, and gender) fixed effects. Standard errors are clustered at the individual level (the unit of randomization).

Table S8. Perceived Quality by Reading Grade Level, without Controls - Disaggregated

	(1)	(2)	(3)	(4)	(5)	(6)	(7)	(8)	(9)	(10)
	Title sparked curiosity	Appropriate level of detail	Clear rationale	Visual presentation	Appropriate tone	Adequate length	Easy to understand	Clear proposed recommendations	Relevant proposed recommendations	Sufficiently detailed recommendations
(1) AI-Generated	0.015	0.344	-2.829*	-2.416	-0.910	-0.814	-0.149	-0.182	-1.622	1.984
	(1.683)	(1.761)	(1.687)	(1.984)	(1.465)	(2.131)	(1.558)	(1.562)	(1.713)	(1.978)
RI p-value	[.994]	[.785]	[.02]	[.058]	[.394]	[.546]	[.878]	[.861]	[.134]	[.13]
(2) AI-Reported	-0.451	1.191	-1.993	0.291	0.300	-0.707	-1.044	-1.119	-1.924	0.433
	(1.757)	(2.004)	(1.959)	(2.293)	(1.595)	(2.318)	(1.726)	(1.948)	(2.054)	(2.424)
RI p-value	[.8]	[.566]	[.361]	[.893]	[.846]	[.749]	[.536]	[.569]	[.392]	[.879]
(3) AI-Generated × AI-Reported	1.678	0.732	3.424	2.130	0.304	2.179	1.349	1.048	3.059	-0.373
	(2.006)	(2.263)	(2.137)	(2.503)	(1.735)	(2.578)	(1.895)	(2.129)	(2.219)	(2.630)
RI p-value (Permuting AI-Generated)	[.289]	[.677]	[.021]	[.278]	[.819]	[.211]	[.286]	[.499]	[.059]	[.842]
RI p-value (Permuting AI-Reported)	[.421]	[.751]	[.134]	[.424]	[.858]	[.413]	[.487]	[.634]	[.195]	[.886]
(4) Reading Grade Level	0.019	0.125	-0.078	0.016	0.034	0.103	0.017	0.012	-0.018	0.182
	(0.090)	(0.102)	(0.096)	(0.116)	(0.080)	(0.113)	(0.089)	(0.088)	(0.099)	(0.116)
RI p-value	[.7]	[.028]	[.056]	[.757]	[.38]	[.02]	[.689]	[.812]	[.704]	[.001]
(5) AI-Generated × Reading Grade Level	-0.014	-0.022	0.167	0.142	0.049	0.055	0.003	0.013	0.115	-0.114
	(0.102)	(0.105)	(0.101)	(0.118)	(0.088)	(0.131)	(0.094)	(0.093)	(0.104)	(0.117)
RI p-value (Permuting AI-Generated)	[.844]	[.78]	[.023]	[.074]	[.444]	[.515]	[.96]	[.855]	[.088]	[.149]
RI p-value (Permuting Reading Grade Level)	[.793]	[.701]	[.011]	[.156]	[.464]	[.387]	[.949]	[.791]	[.082]	[.119]
(6) AI-Reported × Reading Grade Level	0.021	-0.077	0.123	-0.025	-0.018	0.039	0.058	0.061	0.108	-0.036
	(0.103)	(0.116)	(0.115)	(0.134)	(0.093)	(0.137)	(0.101)	(0.113)	(0.120)	(0.141)
RI p-value (Permuting AI-Reported)	[.839]	[.524]	[.335]	[.856]	[.841]	[.767]	[.567]	[.589]	[.401]	[.827]
RI p-value (Permuting Reading Grade Level)	[.732]	[.424]	[.01]	[.83]	[.801]	[.745]	[.269]	[.327]	[.055]	[.831]
(7) AI-Generated × AI-Reported × Reading Grade Level	-0.084	-0.051	-0.202	-0.122	-0.013	-0.129	-0.074	-0.051	-0.189	0.041
	(0.123)	(0.136)	(0.128)	(0.149)	(0.103)	(0.158)	(0.114)	(0.126)	(0.133)	(0.157)
RI p-value (Permuting AI-Generated)	[.391]	[.624]	[.031]	[.305]	[.877]	[.243]	[.355]	[.574]	[.056]	[.723]
RI p-value (Permuting AI-Reported)	[.509]	[.72]	[.144]	[.449]	[.906]	[.426]	[.517]	[.7]	[.182]	[.797]
RI p-value (Permuting Reading Grade Level)	[.276]	[.542]	[.02]	[.273]	[.817]	[.074]	[.19]	[.456]	[.047]	[.763]
Mean of Human-Generated and Human-Reported	3.107	2.929	2.893	2.583	3.083	3.071	3.25	3.012	2.94	2.667
(1) + (5): Effect of AI-Generated, +1 Grade	0.001	0.322	-2.662*	-2.273	-0.861	-0.759	-0.146	-0.169	-1.507	1.870
(2) + (6): Effect of AI-Reported, +1 Grade	-0.430	1.114	-1.871	0.267	0.282	-0.669	-0.987	-1.058	-1.816	0.397
(3) + (7): Effect of AI-Generated × AI-Reported, +1 Grade	1.594	0.682	3.222	2.008	0.291	2.050	1.275	0.996	2.870	-0.332
(1) + (2) + (3): Total Effect of AI, +1 Grade	1.242	2.268	-1.398	0.005	-0.307	0.658	0.155	-0.254	-0.486	2.043
(1) + (2) + (3) + (5) + (6) + (7): Total Effect of AI, +1 Grade	1.166	2.118	-1.310	0.001	-0.288	0.623	0.142	-0.231	-0.453	1.935
(5) + (6) + (7): Difference in Total Effect of AI, +1 Grade	-0.077	-0.150	0.088	-0.004	0.019	-0.036	-0.013	0.022	0.034	-0.108
Observations	366	366	366	366	366	366	366	366	366	366

Notes: * $p<0.1$, ** $p<0.05$, *** $p<0.01$. This table reports the heterogeneous effects of the treatments on perceptions of the blog's quality by the reading level of the blog for each of the 10 quality attributes separately. The Flesch-Kincaid reading-level measure is used to construct a continuous measure of the grade level of the blogs Flesch1948. We include the grade level of the blog and interact it with indicators for AI-generated blogs, AI-reported blogs, and their interaction (resulting in a triple interaction). Perceptions are measured using a 5-point Likert scale ranging from 'strongly disagree' that the blog is high-quality with respect to this attribute (0) to 'strongly agree' (4). An indicator variable is assigned a value of 1 if the response is either 'agree' or 'strongly agree' while 'neutral', 'disagree', and 'strongly disagree' are assigned a value of 0. We control for wave and strata (country, seniority, and gender) fixed effects. Standard errors are clustered at the individual level (the unit of randomization).

Table S9. Perceived Quality of the Blog, with Controls - Disaggregated

	(1)	(2)	(3)	(4)	(5)	(6)	(7)	(8)	(9)	(10)
	Title sparked curiosity	Appropriate level of detail	Clear rationale	Visual presentation	Appropriate tone	Adequate length	Easy to understand	Clear proposed recommendations	Relevant proposed recommendations	Sufficiently detailed recommendations
(1) AI-Generated	-0.213*	-0.278*	-0.227	-0.364**	-0.287**	-0.282*	-0.102	0.032	0.124	-0.118
	(0.128)	(0.150)	(0.142)	(0.177)	(0.127)	(0.156)	(0.118)	(0.132)	(0.129)	(0.169)
RI p-value	[.109]	[.085]	[.119]	[.038]	[.024]	[.086]	[.424]	[.85]	[.367]	[.485]
(2) AI-Reported	-0.062	-0.158	0.067	-0.124	-0.005	-0.137	-0.066	-0.042	-0.007	-0.168
	(0.141)	(0.145)	(0.135)	(0.174)	(0.112)	(0.158)	(0.115)	(0.142)	(0.148)	(0.171)
RI p-value	[.644]	[.27]	[.593]	[.477]	[.97]	[.342]	[.566]	[.782]	[.96]	[.332]
(3) AI-Generated × AI-Reported	0.401**	0.282	0.266	0.447*	0.166	0.302	0.128	0.123	0.008	0.350
	(0.203)	(0.213)	(0.194)	(0.248)	(0.163)	(0.218)	(0.158)	(0.192)	(0.191)	(0.241)
RI p-value (Permuting AI-Generated)	[.059]	[.194]	[.161]	[.08]	[.302]	[.173]	[.454]	[.524]	[.97]	[.171]
RI p-value (Permuting AI-Reported)	[.048]	[.197]	[.187]	[.08]	[.327]	[.2]	[.438]	[.560]	[.971]	[.164]
Mean of Human-Generated and Human-Reported	3.107	2.929	2.893	2.583	3.083	3.071	3.25	3.012	2.94	2.667
(1) + (3): Total Effect of AI-Generated	0.188	0.005	0.039	0.083	-0.121	0.020	0.026	0.156	0.132	0.232
(1) + (2) + (3): Total Effect of AI	0.126	-0.154	0.106	-0.041	-0.125	-0.117	-0.040	0.113	0.125	0.065
Observations	366	366	366	366	366	366	366	366	366	366

Notes: * p<0.1, ** p<0.05, *** p<0.01. This table reports the effects of the treatments on perceptions of the policy blog's quality for each of the 10 quality attributes separately. Perceptions are measured using a 5-point Likert scale ranging from 'strongly disagree' that the blog is high-quality with respect to this attribute (0) to 'strongly agree' (4). An indicator variable is assigned a value of 1 if the response is 'strongly agree' or 'agree', while 'neutral', 'disagree', and 'strongly disagree' are assigned a value of 0. We control for respondent characteristics, wave, and strata (country, seniority, and gender) fixed effects. Standard errors are clustered at the individual level (the unit of randomization).

Table S10. Perceived Quality of the Blog, without Controls - Disaggregated

	(1)	(2)	(3)	(4)	(5)	(6)	(7)	(8)	(9)	(10)
	Title sparked curiosity	Appropriate level of detail	Clear rationale	Visual presentation	Appropriate tone	Adequate length	Easy to understand	Clear proposed recommendations	Relevant proposed recommendations	Sufficiently detailed recommendations
(1) AI-Generated	-0.226*	-0.285*	-0.221	-0.398**	-0.280**	-0.278*	-0.152	-0.024	0.080	-0.111
	(0.128)	(0.154)	(0.143)	(0.169)	(0.124)	(0.153)	(0.114)	(0.138)	(0.134)	(0.162)
RI p-value	[.111]	[.071]	[.119]	[.023]	[.033]	[.066]	[.184]	[.873]	[.57]	[.503]
(2) AI-Reported	-0.088	-0.109	0.091	-0.125	-0.009	-0.047	-0.065	-0.085	-0.088	-0.173
	(0.134)	(0.144)	(0.133)	(0.172)	(0.108)	(0.145)	(0.114)	(0.140)	(0.146)	(0.167)
RI p-value	[.495]	[.434]	[.488]	[.466]	[.932]	[.783]	[.552]	[.543]	[.544]	[.285]
(3) AI-Generated × AI-Reported	0.403**	0.189	0.197	0.432*	0.166	0.211	0.136	0.150	0.051	0.309
	(0.189)	(0.211)	(0.191)	(0.242)	(0.161)	(0.210)	(0.160)	(0.196)	(0.195)	(0.234)
RI p-value (Permuting AI-Generated)	[.061]	[.362]	[.286]	[.059]	[.302]	[.322]	[.411]	[.451]	[.803]	[.179]
RI p-value (Permuting AI-Reported)	[.048]	[.379]	[.288]	[.073]	[.31]	[.343]	[.406]	[.426]	[.773]	[.178]
Mean of Human-Generated and Human-Reported	3.107	2.929	2.893	2.583	3.083	3.071	3.25	3.012	2.94	2.667
(1) + (3): Total Effect of AI-Generated	0.177	-0.096	-0.025	0.033	-0.114	-0.067	-0.016	0.127	0.131	0.198
(1) + (2) + (3): Total Effect of AI	0.089	-0.206	0.067	-0.092	-0.123	-0.114	-0.081	0.041	0.043	0.025
Observations	366	366	366	366	366	366	366	366	366	366

Notes: * $p<0.1$, ** $p<0.05$, *** $p<0.01$. This table reports the effects of the treatments on perceptions of the policy blog's quality for each of the 10 quality attributes separately. Perceptions are measured using a 5-point Likert scale ranging from 'strongly disagree' that the blog is high-quality with respect to this attribute (0) to 'strongly agree' (4). An indicator variable is assigned a value of 1 if the response is 'strongly agree' or 'agree', while 'neutral', 'disagree', and 'strongly disagree' are assigned a value of 0. We control for wave and strata (country, seniority, and gender) fixed effects. Standard errors are clustered at the individual level (the unit of randomization).

Table S11. Perceived Quality by Previously used AI, with Controls

	(1)	(2)	(3)	(4)	(5)	(6)
	Unweighted Average Quality (0-4) (10 Attributes)	Weighted Average Quality (0-4) (9 Attributes)	Unweighted average quality (0-4) (10 Attributes)	Standardized First Principal Component (10 Attributes)	Number of 'agree' or 'strongly agree' statements (0-10)	Overall quality of brief rated 'high' or 'very high'
(1) AI-Generated	-0.535**	-0.538**	-0.518**	-0.775**	-2.596***	-0.238*
	(0.210)	(0.212)	(0.213)	(0.305)	(0.945)	(0.141)
RI p-value	[.021]	[.021]	[.029]	[.023]	[.008]	[.087]
(2) AI-Reported	-0.462**	-0.479**	-0.472**	-0.721**	-1.770	-0.181
	(0.209)	(0.217)	(0.216)	(0.306)	(1.091)	(0.162)
RI p-value	[.027]	[.028]	[.031]	[.019]	[.092]	[.255]
(3) AI-Generated × AI-Reported	0.855***	0.885***	0.847**	1.293***	3.175**	0.400*
	(0.329)	(0.342)	(0.338)	(0.479)	(1.551)	(0.240)
RI p-value (Permuting AI-Generated)	[.02]	[.021]	[.024]	[.017]	[.047]	[.099]
RI p-value (Permuting AI-Reported)	[.016]	[.013]	[.017]	[.015]	[.051]	[.092]
(4) Previously used AI	-0.221	-0.223	-0.204	-0.340	-1.182	-0.056
	(0.169)	(0.174)	(0.174)	(0.247)	(0.813)	(0.129)
RI p-value	[.443]	[.481]	[.515]	[.411]	[.307]	[.717]
(5) AI-Generated × Previously used AI	0.396	0.431	0.394	0.603	1.947	0.091
	(0.267)	(0.273)	(0.271)	(0.386)	(1.195)	(0.183)
RI p-value (Permuting AI-Generated)	[.129]	[.104]	[.138]	[.104]	[.106]	[.601]
RI p-value (Permuting Previously used AI)	[.237]	[.226]	[.257]	[.222]	[.184]	[.681]
(6) AI-Reported × Previously used AI	0.477*	0.510*	0.481*	0.759**	1.502	0.065
	(0.257)	(0.264)	(0.265)	(0.378)	(1.280)	(0.196)
RI p-value (Permuting AI-Reported)	[.046]	[.043]	[.056]	[.032]	[.228]	[.743]
RI p-value (Permuting Previously used AI)	[.212]	[.198]	[.228]	[.186]	[.326]	[.77]
(7) AI-Generated × AI-Reported × Previously used AI	-0.690*	-0.740*	-0.669*	-1.115*	-2.185	-0.171
	(0.390)	(0.405)	(0.402)	(0.569)	(1.793)	(0.276)
RI p-value (Permuting AI-Generated)	[.08]	[.072]	[.093]	[.044]	[.206]	[.556]
RI p-value (Permuting AI-Reported)	[.081]	[.069]	[.098]	[.055]	[.232]	[.532]
RI p-value (Permuting Previously used AI)	[.119]	[.104]	[.138]	[.094]	[.218]	[.561]
Mean of Human-Generated and Human-Reported	2.954	2.943	2.939	.141	7.75	.702
(1) + (5): Effect of AI-Generated, Used AI= 1	-0.139	-0.108	-0.124	-0.172	-0.649	-0.147
(2) + (6): Effect of AI-Reported, Used AI= 1	0.015	0.031	0.009	0.038	-0.268	-0.116
(3) + (7): Effect of AI-Generated × AI-Reported, Used AI= 1	0.165	0.144	0.178	0.177	0.989	0.229
(1) + (2) + (3): Total Effect of AI, Used AI= 0	-0.142	-0.133	-0.143	-0.203	-1.191	-0.019
(1) + (2) + (3) + (5) + (6) + (7): Total Effect of AI, Used AI = 1	0.041	0.068	0.063	0.044	0.072	-0.034
(5) + (6) + (7): Difference in Total Effect of AI, Used AI= 0 vs. Used AI= 1	0.183	0.200	0.206	0.247	1.263	-0.015
Observations	296	296	296	296	296	296

Notes: * p<0.1, ** p<0.05, *** p<0.01. This table reports the heterogeneous effects of the treatments on perceptions of the blog's quality by whether the respondent has previously used AI (indicator equal to 1). We include this indicator as well as interact it with indicators for AI-generated blogs, AI-reported blogs, and their interaction (resulting in a triple interaction). Perceptions are measured using a 5-point Likert scale ranging from 'strongly disagree' that the blog is high-quality with respect to this attribute (0) to 'strongly agree' (4). The 10 attributes are: blog had an appropriate amount of detail, a clear rationale, a catchy title, good visual presentation, an appropriate tone, was of adequate length, was easy to understand, and provided clear, relevant, and sufficiently detailed recommendations. The outcome in the first column is the unweighted average across the 10 attributes, and the outcome in the second column is the weighted average across nine attributes (weighted by respondents' reported relative importance of these attributes save appropriate tone). The outcome in the third column is the unweighted average of the nine statements, as a robustness check. The outcome in the fourth column is the standardized score of the first principal component, calculated based on the same 10 attributes using Polychoric PCA. The outcome in the fifth column is the total number of the 10 attributes rated as either 'agree' or 'strongly agree' while 'neutral', 'disagree', and 'strongly disagree' are assigned a value of 0. The outcome in the sixth column is an indicator variable equal to 1 if the overall quality of the brief is rated either 'high' or 'very high' from a separate survey question, while 'average', 'low', and 'very low' are coded as 0. We control for respondent characteristics, wave, and strata (country, seniority, and gender) fixed effects. Standard errors are clustered at the individual level (the unit of randomization).

Table S12. Perceived Quality by Previously used AI, without Controls

	(1)	(2)	(3)	(4)	(5)	(6)
	Unweighted Average Quality (0-4) (10 Attributes)	Weighted Average Quality (0-4) (9 Attributes)	Unweighted average quality (0-4) (10 Attributes)	Standardized First Principal Component (10 Attributes)	Number of 'agree' or 'strongly agree' statements (0-10)	Overall quality of brief rated 'high' or 'very high'
(1) AI-Generated	-0.574**	-0.583**	-0.564**	-0.811**	-2.611**	-0.263*
	(0.238)	(0.242)	(0.244)	(0.343)	(1.025)	(0.142)
RI p-value	[.02]	[.012]	[.025]	[.023]	[.012]	[.041]
(2) AI-Reported	-0.421**	-0.436**	-0.432**	-0.660**	-1.398	-0.192
	(0.211)	(0.217)	(0.218)	(0.309)	(1.031)	(0.173)
RI p-value	[.056]	[.056]	[.069]	[.042]	[.195]	[.238]
(3) AI-Generated × AI-Reported	0.826**	0.860**	0.822**	1.241**	2.840*	0.407*
	(0.348)	(0.361)	(0.359)	(0.507)	(1.528)	(0.243)
RI p-value (Permuting AI-Generated)	[.02]	[.019]	[.031]	[.029]	[.063]	[.105]
RI p-value (Permuting AI-Reported)	[.019]	[.019]	[.029]	[.016]	[.074]	[.079]
(4) Previously used AI	-0.144	-0.150	-0.129	-0.225	-0.797	0.006
	(0.180)	(0.183)	(0.184)	(0.263)	(0.823)	(0.124)
RI p-value	[.605]	[.627]	[.656]	[.589]	[.516]	[.97]
(5) AI-Generated × Previously used AI	0.360	0.396	0.359	0.531	1.755	0.087
	(0.284)	(0.288)	(0.289)	(0.409)	(1.251)	(0.183)
RI p-value (Permuting AI-Generated)	[.211]	[.147]	[.177]	[.183]	[.158]	[.615]
RI p-value (Permuting Previously used AI)	[.223]	[.209]	[.235]	[.25]	[.188]	[.678]
(6) AI-Reported × Previously used AI	0.399	0.429	0.403	0.655*	1.050	0.069
	(0.256)	(0.263)	(0.264)	(0.376)	(1.218)	(0.199)
RI p-value (Permuting AI-Reported)	[.127]	[.082]	[.126]	[.094]	[.385]	[.727]
RI p-value (Permuting Previously used AI)	[.33]	[.287]	[.328]	[.278]	[.521]	[.762]
(7) AI-Generated × AI-Reported × Previously used AI	-0.666*	-0.725*	-0.651	-1.084*	-2.013	-0.199
	(0.400)	(0.412)	(0.411)	(0.584)	(1.762)	(0.279)
RI p-value (Permuting AI-Generated)	[.113]	[.073]	[.11]	[.08]	[.25]	[.445]
RI p-value (Permuting AI-Reported)	[.099]	[.068]	[.122]	[.053]	[.233]	[.455]
RI p-value (Permuting Previously used AI)	[.116]	[.095]	[.123]	[.07]	[.238]	[.503]
Mean of Human-Generated and Human-Reported	2.954	2.943	2.939	.141	7.75	.702
(1) + (5): Effect of AI-Generated, Used AI= 1	-0.215	-0.187	-0.205	-0.279	-0.856	-0.176*
(2) + (6): Effect of AI-Reported, Used AI= 1	-0.021	-0.007	-0.029	-0.004	-0.348	-0.122
(3) + (7): Effect of AI-Generated × AI-Reported, Used AI= 1	0.160	0.135	0.172	0.157	0.826	0.208
(1) + (2) + (3): Total Effect of AI, Used AI= 0	-0.169	-0.159	-0.173	-0.229	-1.170	-0.047
(1) + (2) + (3) + (5) + (6) + (7): Total Effect of AI, Used AI= 1	-0.076	-0.059	-0.062	-0.126	-0.378	-0.090
(5) + (6) + (7): Difference in Total Effect of AI, Used AI= 0 vs. Used AI= 1	0.092	0.100	0.111	0.103	0.792	-0.043
Observations	296	296	296	296	296	296

Notes: * p<0.1, ** p<0.05, *** p<0.01. This table reports the heterogeneous effects of the treatments on perceptions of the blog's quality by whether the respondent has previously used AI (indicator equal to 1). We include this indicator as well as interact it with indicators for AI-generated blogs, AI-reported blogs, and their interaction (resulting in a triple interaction). Perceptions are measured using a 5-point Likert scale ranging from 'strongly disagree' that the blog is high-quality with respect to this attribute (0) to 'strongly agree' (4). The 10 attributes are: blog had an appropriate amount of detail, a clear rationale, a catchy title, good visual presentation, an appropriate tone, was of adequate length, was easy to understand, and provided clear, relevant, and sufficiently detailed recommendations. The outcome in the first column is the unweighted average across the 10 attributes, and the outcome in the second column is the weighted average across nine attributes (weighted by respondents' reported relative importance of these attributes save appropriate tone). The outcome in the third column is the unweighted average of the nine statements, as a robustness check. The outcome in the fourth column is the standardized score of the first principal component, calculated based on the same 10 attributes using Polychoric PCA. The outcome in the fifth column is the total number of the 10 attributes rated as either 'agree' or 'strongly agree', while 'neutral', 'disagree', and 'stronly disagree' are assigned a value of 0. The outcome in the sixth column is an indicator variable equal to 1 if the overall quality of the brief is rated either 'high' or 'very high' from a separate survey question, while 'average', 'low', and 'very low' are coded as 0. We control for wave and strata (country, seniority, and gender) fixed effects. Standard errors are clustered at the individual level (the unit of randomization).

Table S13. Perceived Quality by Previously used AI, with Controls - Disaggregated

	(1)	(2)	(3)	(4)	(5)	(6)	(7)	(8)	(9)	(10)
	Title sparked curiosity	Appropriate level of detail	Clear rationale	Visual presentation	Appropriate tone	Adequate length	Easy to understand	Clear proposed recommendations	Relevant proposed recommendations	Sufficiently detailed recommendations
(1) AI-Generated	-0.075	-0.489	-0.703**	-0.518	-0.693**	-0.461	-0.563**	-0.590**	-0.493*	-0.768**
	(0.302)	(0.334)	(0.341)	(0.385)	(0.320)	(0.338)	(0.246)	(0.257)	(0.289)	(0.352)
RI p-value	[.815]	[.14]	[.055]	[.212]	[.036]	[.17]	[.025]	[.045]	[.114]	[.025]
(2) AI-Reported	-0.514	-0.002	-0.372	-0.685*	-0.371	-0.578**	-0.279	-0.616*	-0.827***	-0.371
	(0.362)	(0.292)	(0.356)	(0.403)	(0.262)	(0.285)	(0.228)	(0.341)	(0.315)	(0.353)
RI p-value	[.147]	[.997]	[.277]	[.11]	[.131]	[.044]	[.233]	[.057]	[.017]	[.291]
(3) AI-Generated × AI-Reported	0.575	0.321	0.745	1.258**	0.928**	0.669	0.679*	1.135**	1.151**	1.089*
	(0.482)	(0.484)	(0.494)	(0.538)	(0.408)	(0.517)	(0.348)	(0.473)	(0.458)	(0.560)
RI p-value (Permuting AI-Generated)	[.232]	[.539]	[.156]	[.033]	[.026]	[.23]	[.078]	[.025]	[.017]	[.09]
RI p-value (Permuting AI-Reported)	[.237]	[.521]	[.143]	[.025]	[.023]	[.223]	[.073]	[.031]	[.02]	[.059]
(4) Previously used AI	-0.178	0.176	-0.453	-0.034	-0.370*	-0.261	-0.364*	-0.271	-0.377	-0.074
	(0.239)	(0.263)	(0.291)	(0.380)	(0.195)	(0.261)	(0.212)	(0.253)	(0.252)	(0.310)
RI p-value	[.457]	[.557]	[.126]	[.917]	[.057]	[.49]	[.315]	[.459]	[.311]	[.872]
(5) AI-Generated × Previously used AI	-0.100	0.121	0.551	-0.005	0.418	0.152	0.620**	0.758**	0.671*	0.778*
	(0.361)	(0.409)	(0.422)	(0.475)	(0.382)	(0.405)	(0.302)	(0.336)	(0.369)	(0.442)
RI p-value (Permuting AI-Generated)	[.791]	[.773]	[.184]	[.991]	[.232]	[.704]	[.045]	[.019]	[.072]	[.064]
RI p-value (Permuting Previously used AI)	[.773]	[.78]	[.211]	[.992]	[.245]	[.73]	[.233]	[.134]	[.081]	[.227]
(6) AI-Reported × Previously used AI	0.697*	-0.214	0.468	0.631	0.439	0.653*	0.255	0.572	0.935**	0.334
	(0.408)	(0.359)	(0.415)	(0.484)	(0.306)	(0.365)	(0.300)	(0.412)	(0.377)	(0.436)
RI p-value (Permuting AI-Reported)	[.078]	[.547]	[.219]	[.202]	[.119]	[.06]	[.382]	[.141]	[.013]	[.426]
RI p-value (Permuting Previously used AI)	[.105]	[.668]	[.329]	[.235]	[.203]	[.244]	[.519]	[.186]	[.097]	[.564]
(7) AI-Generated × AI-Reported × Previously used AI	-0.255	0.035	-0.507	-0.777	-0.881*	-0.507	-0.632	-1.123**	-1.202**	-1.051
	(0.543)	(0.557)	(0.572)	(0.648)	(0.462)	(0.602)	(0.416)	(0.571)	(0.536)	(0.655)
RI p-value (Permuting AI-Generated)	[.618]	[.952]	[.402]	[.229]	[.048]	[.394]	[.125]	[.05]	[.026]	[.139]
RI p-value (Permuting AI-Reported)	[.638]	[.957]	[.365]	[.251]	[.065]	[.413]	[.146]	[.05]	[.035]	[.117]
RI p-value (Permuting Previously used AI)	[.621]	[.955]	[.379]	[.213]	[.082]	[.407]	[.22]	[.061]	[.047]	[.196]
Mean of Human-Generated and Human-Reported	3.107	2.929	2.893	2.583	3.083	3.071	3.25	3.012	2.94	2.667
(1) + (5): Effect of AI-Generated, Used = 1	-0.175	-0.368*	-0.153	-0.523**	-0.275	-0.310	0.057	0.169	0.177	0.010
(2) + (6): Effect of AI-Reported, Used = 1	0.182	-0.216	0.095	-0.054	0.068	0.074	-0.025	-0.044	0.108	-0.037
(3) + (7): Effect of AI-Generated × AI-Reported, Used = 1	0.320	0.356	0.237	0.481	0.047	0.162	0.047	0.012	-0.051	0.038
(1) + (2) + (3): Total Effect of AI, Used = 0	-0.014	-0.170	-0.331	0.054	-0.135	-0.371	-0.164	-0.071	-0.169	-0.051
(1) + (2) + (3) + (5) + (6) + (7): Total Effect of AI, Used = 1	0.327*	-0.228	0.180	-0.096	-0.160	-0.073	0.079	0.136	0.235	0.010
(5) + (6) + (7): Difference in Total Effect of AI, Used = 0 vs. Used = 1	0.341	-0.058	0.511	-0.150	-0.024	0.297	0.243	0.207	0.403	0.061
Observations	296	296	296	296	296	296	296	296	296	296

Notes: * p<0.1, ** p<0.05, *** p<0.01. This table reports the heterogeneous effects of the treatments on perceptions of the blog's quality by whether the respondent has previously used AI (indicator equal to 1) for each of the 10 quality attributes separately. We include this indicator and interact it with indicators for AI-generated blogs, AI-reported blogs, and their interaction (resulting in a triple interaction). Perceptions are measured using a 5-point Likert scale ranging from 'strongly disagree' that the blog is high-quality with respect to this attribute (0) to 'strongly agree' (4). An indicator variable is assigned a value of 1 if the response is 'strongly agree' or 'agree', while 'neutral', 'disagree', and 'strongly disagree' are assigned a value of 0. We control for respondent characteristics, wave, and strata (country, seniority, and gender) fixed effects. Standard errors are clustered at the individual level (the unit of randomization).

Table S14. Perceived Quality by Previously used AI, without Controls - Disaggregated

	(1)	(2)	(3)	(4)	(5)	(6)	(7)	(8)	(9)	(10)
	Title sparked curiosity	Appropriate level of detail	Clear rationale	Visual presentation	Appropriate tone	Adequate length	Easy to understand	Clear proposed recommendations	Relevant proposed recommendations	Sufficiently detailed recommendations
(1) AI-Generated	-0.219	-0.511	-0.724**	-0.603	-0.666**	-0.465	-0.558*	-0.610*	-0.576*	-0.809**
	(0.317)	(0.354)	(0.345)	(0.395)	(0.311)	(0.343)	(0.296)	(0.313)	(0.306)	(0.333)
RI p-value	[.503]	[.15]	[.043]	[.126]	[.031]	[.166]	[.059]	[.06]	[.062]	[.018]
(2) AI-Reported	-0.627*	0.064	-0.307	-0.543	-0.319	-0.473*	-0.249	-0.547	-0.897***	-0.308
	(0.342)	(0.307)	(0.368)	(0.401)	(0.244)	(0.279)	(0.241)	(0.340)	(0.287)	(0.348)
RI p-value	[.067]	[.862]	[.406]	[.21]	[.163]	[.107]	[.324]	[.116]	[.004]	[.371]
(3) AI-Generated × AI-Reported	0.702	0.241	0.642	1.155**	0.859**	0.634	0.674*	1.095**	1.234***	1.024*
	(0.491)	(0.498)	(0.514)	(0.534)	(0.396)	(0.528)	(0.391)	(0.512)	(0.437)	(0.557)
RI p-value (Permuting AI-Generated)	[.138]	[.659]	[.24]	[.035]	[.032]	[.268]	[.087]	[.027]	[.002]	[.086]
RI p-value (Permuting AI-Reported)	[.138]	[.687]	[.194]	[.055]	[.035]	[.213]	[.086]	[.042]	[.012]	[.059]
(4) Previously used AI	-0.155	0.221	-0.357	0.012	-0.283	-0.097	-0.204	-0.108	-0.417*	-0.052
	(0.225)	(0.277)	(0.293)	(0.357)	(0.202)	(0.256)	(0.212)	(0.268)	(0.244)	(0.302)
RI p-value	[.539]	[.436]	[.245]	[.971]	[.157]	[.75]	[.624]	[.765]	[.188]	[.903]
(5) AI-Generated × Previously used AI	-0.025	0.050	0.515	0.037	0.367	0.041	0.527	0.668*	0.688*	0.727*
	(0.369)	(0.416)	(0.410)	(0.478)	(0.375)	(0.411)	(0.340)	(0.373)	(0.372)	(0.423)
RI p-value (Permuting AI-Generated)	[.945]	[.914]	[.189]	[.932]	[.307]	[.9]	[.102]	[.065]	[.06]	[.09]
RI p-value (Permuting Previously used AI)	[.95]	[.912]	[.21]	[.932]	[.299]	[.915]	[.314]	[.141]	[.062]	[.22]
(6) AI-Reported × Previously used AI	0.753*	-0.260	0.430	0.448	0.364	0.531	0.187	0.437	0.914***	0.188
	(0.388)	(0.369)	(0.410)	(0.471)	(0.290)	(0.350)	(0.301)	(0.406)	(0.353)	(0.428)
RI p-value (Permuting AI-Reported)	[.046]	[.514]	[.3]	[.383]	[.185]	[.096]	[.517]	[.245]	[.018]	[.64]
RI p-value (Permuting Previously used AI)	[.085]	[.57]	[.371]	[.378]	[.253]	[.325]	[.688]	[.292]	[.076]	[.713]
(7) AI-Generated × AI-Reported × Previously used AI	-0.372	0.075	-0.483	-0.679	-0.809*	-0.457	-0.630	-1.069*	-1.286**	-0.954
	(0.558)	(0.563)	(0.570)	(0.644)	(0.468)	(0.606)	(0.455)	(0.583)	(0.507)	(0.650)
RI p-value (Permuting AI-Generated)	[.479]	[.905]	[.415]	[.291]	[.067]	[.46]	[.145]	[.074]	[.018]	[.162]
RI p-value (Permuting AI-Reported)	[.499]	[.912]	[.399]	[.35]	[.068]	[.439]	[.173]	[.054]	[.02]	[.126]
RI p-value (Permuting Previously used AI)	[.474]	[.901]	[.373]	[.269]	[.094]	[.389]	[.219]	[.039]	[.032]	[.186]
Mean of Human-Generated and Human-Reported	3.107	2.929	2.893	2.583	3.083	3.071	3.25	3.012	2.94	2.667
(1) + (5): Effect of AI-Generated, Used AI= 1	-0.244	-0.461**	-0.209	-0.566**	-0.299*	-0.424**	-0.031	0.058	0.112	-0.082
(2) + (6): Effect of AI-Reported, Used AI = 1	0.126	-0.196	0.123	-0.095	0.045	0.058	-0.062	-0.110	0.017	-0.120
(3) + (7): Effect of AI-Generated × AI-Reported, Used AI= 1	0.330	0.316	0.159	0.476	0.050	0.178	0.044	0.026	-0.052	0.071
(1) + (2) + (3): Total Effect of AI, Used AI= 0	-0.144	-0.206	-0.389	0.008	-0.127	-0.303	-0.133	-0.062	-0.239	-0.093
(1) + (2) + (3) + (5) + (6) + (7): Total Effect of AI, Used AI= 1	0.212	-0.341*	0.073	-0.184	-0.205	-0.188	-0.049	-0.027	0.077	-0.131
(5) + (6) + (7): Difference in Total Effect of AI, Used AI= 0 vs. Used AI= 1	0.356	-0.135	0.462	-0.193	-0.078	0.115	0.084	0.036	0.316	-0.038
Observations	296	296	296	296	296	296	296	296	296	296

Notes: * p<0.1, ** p<0.05, *** p<0.01. This table reports the heterogeneous effects of the treatments on perceptions of the blog's quality by whether the respondent has previously used AI (indicator equal to 1) for each of the 10 quality attributes separately. We include this indicator and interact it with indicators for AI-generated blogs, AI-reported blogs, and their interaction (resulting in a triple interaction). Perceptions are measured using a 5-point Likert scale ranging from 'strongly disagree' that the blog is high-quality with respect to this attribute (0) to 'strongly agree' (4). An indicator variable is assigned a value of 1 if the response is 'strongly agree' or 'agree', while 'neutral', 'disagree', and 'strongly disagree' are assigned a value of 0. We control for wave and strata (country, seniority, and gender) fixed effects. Standard errors are clustered at the individual level (the unit of randomization).

Table S15. Perceived Quality by Trust in AI blog writing, with Controls

	(1)	(2)	(3)	(4)	(5)	(6)
	Unweighted Average Quality (0-4) (10 Attributes)	Weighted Average Quality (0-4) (9 Attributes)	Unweighted average quality (0-4) (10 Attributes)	Standardized First Principal Component (10 Attributes)	Number of 'agree' or 'strongly agree' statements (0-10)	Overall quality of brief rated 'high' or 'very high'
(1) AI-Generated	-0.229	-0.217	-0.213	-0.327	-1.012	-0.212*
	(0.177)	(0.182)	(0.182)	(0.259)	(0.769)	(0.116)
RI p-value	[.205]	[.244]	[.256]	[.205]	[.219]	[.064]
(2) AI-Reported	-0.048	-0.023	-0.041	-0.074	-0.215	-0.087
	(0.164)	(0.172)	(0.173)	(0.244)	(0.725)	(0.115)
RI p-value	[.772]	[.91]	[.810]	[.765]	[.79]	[.449]
(3) AI-Generated × AI-Reported	0.205	0.192	0.205	0.240	1.104	0.177
	(0.250)	(0.261)	(0.259)	(0.364)	(1.079)	(0.177)
RI p-value (Permuting AI-Generated)	[.427]	[.486]	[.448]	[.53]	[.308]	[.314]
RI p-value (Permuting AI-Reported)	[.399]	[.469]	[.425]	[.497]	[.289]	[.289]
(4) Trust in AI blog writing	0.173	0.202	0.201	0.206	1.177*	0.101
	(0.140)	(0.144)	(0.144)	(0.204)	(0.643)	(0.108)
RI p-value	[.177]	[.132]	[.127]	[.258]	[.057]	[.341]
(5) AI-Generated × Trust in AI blog writing	0.073	0.080	0.065	0.139	-0.008	0.080
	(0.224)	(0.230)	(0.228)	(0.326)	(0.967)	(0.163)
RI p-value (Permuting AI-Generated)	[.747]	[.73]	[.768]	[.663]	[.997]	[.617]
RI p-value (Permuting Trust in AI blog writing)	[.723]	[.706]	[.751]	[.649]	[.994]	[.598]
(6) AI-Reported × Trust in AI blog writing	-0.076	-0.114	-0.104	-0.094	-0.646	-0.068
	(0.209)	(0.218)	(0.218)	(0.310)	(0.907)	(0.157)
RI p-value (Permuting AI-Reported)	[.715]	[.619]	[.639]	[.756]	[.51]	[.667]
RI p-value (Permuting Trust in AI blog writing)	[.665]	[.559]	[.582]	[.723]	[.464]	[.646]
(7) AI-Generated × AI-Reported × Trust in AI blog writing	0.087	0.100	0.104	0.155	0.326	0.088
	(0.315)	(0.327)	(0.325)	(0.458)	(1.355)	(0.232)
RI p-value (Permuting AI-Generated)	[.781]	[.765]	[.759]	[.725]	[.783]	[.712]
RI p-value (Permuting AI-Reported)	[.799]	[.763]	[.758]	[.738]	[.816]	[.692]
RI p-value (Permuting Trust in AI blog writing)	[.752]	[.716]	[.713]	[.699]	[.776]	[.684]
Mean of Human-Generated and Human-Reported	2.954	2.943	2.939	.141	7.75	.702
(1) + (5): Effect of AI-Generated, Trust = 1	-0.156	-0.137	-0.148	-0.188	-1.019*	-0.132
(2) + (6): Effect of AI-Reported, Trust = 1	-0.124	-0.136	-0.145	-0.167	-0.861	-0.155
(3) + (7): Effect of AI-Generated × AI-Reported, Trust = 1	0.291*	0.292	0.308*	0.395	1.430*	0.265*
(1) + (2) + (3): Total Effect of AI, Trust = 0	-0.073	-0.048	-0.050	-0.161	-0.123	-0.122
(1) + (2) + (3) + (5) + (6) + (7): Total Effect of AI, Trust = 1	0.011	0.018	0.015	0.040	-0.451	-0.022
(5) + (6) + (7): Difference in Total Effect of AI, Trust = 0 vs. Trust = 1	0.084	0.066	0.065	0.201	-0.328	0.100
Observations	366	366	366	366	366	366

Notes: * $p<0.1$, ** $p<0.05$, *** $p<0.01$. This table reports the heterogeneous effects of the treatments on perceptions of the blog's quality by whether the respondent trusts AI to generate high-quality blogs (indicator equal to 1). We include this indicator as well as interact it with indicators for AI-generated blogs, AI-reported blogs, and their interaction (resulting in a triple interaction). Perceptions are measured using a 5-point Likert scale ranging from 'strongly disagree' that the blog is high-quality with respect to this attribute (0) to 'strongly agree' (4). The 10 attributes are: blog had an appropriate amount of detail, a clear rationale, a catchy title, good visual presentation, an appropriate tone, was of adequate length, was easy to understand, and provided clear, relevant, and sufficiently detailed recommendations. The outcome in the first column is the unweighted average across the 10 attributes, and the outcome in the second column is the weighted average across nine attributes (weighted by respondents' reported relative importance of these attributes save appropriate tone). The outcome in the third column is the unweighted average of the nine statements, as a robustness check. The outcome in the fourth column is the standardized score of the first principal component, calculated based on the same 10 attributes using Polychoric PCA. The outcome in the fifth column is the total number of the 10 attributes rated as either 'agree' or 'strongly agree', while 'neutral', 'disagree', and 'strongly disagree' are assigned a value of 0. The outcome in the sixth column is an indicator variable equal to 1 if the overall quality of the brief is rated either 'high' or 'very high' from a separate survey question, while 'average', 'low' and 'very low' are coded as 0. We control for respondent characteristics, wave, and strata (country, seniority, and gender) fixed effects. Standard errors are clustered at the individual level (the unit of randomization).

Table S16. Perceived Quality by Trust in AI blog writing, without Controls

	(1)	(2)	(3)	(4)	(5)	(6)
	Unweighted Average Quality (0-4) (10 Attributes)	Weighted Average Quality (0-4) (9 Attributes)	Unweighted average quality (0-4) (10 Attributes)	Standardized First Principal Component (10 Attributes)	Number of 'agree' or 'strongly agree' statements (0-10)	Overall quality of brief rated 'high' or 'very high'
(1) AI-Generated	-0.182	-0.166	-0.164	-0.237	-0.846	-0.200*
	(0.175)	(0.180)	(0.181)	(0.254)	(0.754)	(0.109)
RI p-value	[.288]	[.38]	[.363]	[.36]	[.247]	[.069]
(2) AI-Reported	-0.010	0.012	-0.006	-0.001	-0.099	-0.061
	(0.162)	(0.169)	(0.170)	(0.240)	(0.713)	(0.115)
RI p-value	[.961]	[.944]	[.971]	[.995]	[.907]	[.604]
(3) AI-Generated × AI-Reported	0.157	0.141	0.155	0.144	0.948	0.144
	(0.246)	(0.257)	(0.256)	(0.357)	(1.054)	(0.170)
RI p-value (Permuting AI-Generated)	[.525]	[.598]	[.553]	[.679]	[.412]	[.422]
RI p-value (Permuting AI-Reported)	[.518]	[.588]	[.563]	[.667]	[.344]	[.403]
(4) Trust in AI blog writing	0.269**	0.300**	0.298**	0.365*	1.435**	0.124
	(0.133)	(0.137)	(0.136)	(0.195)	(0.609)	(0.103)
RI p-value	[.047]	[.028]	[.045]	[.072]	[.023]	[.228]
(5) AI-Generated × Trust in AI blog writing	-0.035	-0.039	-0.050	-0.049	-0.245	0.051
	(0.213)	(0.218)	(0.218)	(0.310)	(0.919)	(0.150)
RI p-value (Permuting AI-Generated)	[.865]	[.854]	[.827]	[.872]	[.810]	[.74]
RI p-value (Permuting Trust in AI blog writing)	[.884]	[.852]	[.843]	[.864]	[.788]	[.723]
(6) AI-Reported × Trust in AI blog writing	-0.140	-0.168	-0.161	-0.202	-0.784	-0.091
	(0.205)	(0.211)	(0.212)	(0.305)	(0.877)	(0.151)
RI p-value (Permuting AI-Reported)	[.489]	[.438]	[.458]	[.515]	[.393]	[.548]
RI p-value (Permuting Trust in AI blog writing)	[.443]	[.384]	[.41]	[.458]	[.348]	[.555]
(7) AI-Generated × AI-Reported × Trust in AI blog writing	0.134	0.145	0.149	0.248	0.313	0.116
	(0.303)	(0.314)	(0.312)	(0.442)	(1.291)	(0.219)
RI p-value (Permuting AI-Generated)	[.674]	[.654]	[.657]	[.584]	[.827]	[.623]
RI p-value (Permuting AI-Reported)	[.672]	[.649]	[.644]	[.587]	[.810]	[.593]
RI p-value (Permuting Trust in AI blog writing)	[.62]	[.631]	[.605]	[.5621]	[.8]	[.587]
Mean of Human-Generated and Human-Reported	2.954	2.943	2.939	.141	7.75	.702
(1) + (5): Effect of AI-Generated, Trust = 1	-0.217*	-0.205*	-0.214*	-0.286*	-1.091**	-0.149
(2) + (6): Effect of AI-Reported, Trust = 1	-0.150	-0.156	-0.167	-0.203	-0.883*	-0.152
(3) + (7): Effect of AI-Generated × AI-Reported, Trust = 1	0.291*	0.286*	0.305*	0.392	1.261*	0.260*
(1) + (2) + (3): Total Effect of AI, Trust = 0	-0.035	-0.012	-0.015	-0.095	0.003	-0.117
(1) + (2) + (3) + (5) + (6) + (7): Total Effect of AI, Trust = 1	-0.076	-0.074	-0.076	-0.098	-0.713	-0.041
(5) + (6) + (7): Difference in Total Effect of AI, Trust = 0 vs. Trust = 1	-0.041	-0.062	-0.061	-0.003	-0.716	0.076
Observations	366	366	366	366	366	366

Notes: * p<0.1, ** p<0.05, *** p<0.01. This table reports the heterogeneous effects of the treatments on perceptions of the blog's quality by whether the respondent trusts AI to write high quality blogs (indicator equal to 1). We include this indicator as well as interact it with indicators for AI-generated blogs, AI-reported blogs, and their interaction (resulting in a triple interaction). Perceptions are measured using a 5-point Likert scale ranging from 'strongly disagree' that the blog is high-quality with respect to this attribute (0) to 'strongly agree' (4). The 10 attributes are: blog had an appropriate amount of detail, a clear rationale, a catchy title, good visual presentation, an appropriate tone, was of adequate length, was easy to understand, and provided clear, relevant, and sufficiently detailed recommendations. The outcome in the first column is the unweighted average across the 10 attributes, and the outcome in the second column is the weighted average across nine attributes (weighted by respondents' reported relative importance of these attributes save appropriate tone). The outcome in the third column is the unweighted average of the nine statements, as a robustness check. The outcome in the fourth column is the standardized score of the first principal component, calculated based on the same 10 attributes using Polychoric PCA. The outcome in the fifth column is the total number of the 10 attributes rated as either 'agree' or 'strongly agree', while 'neutral', 'disagree', and 'strongly disagree' are assigned a value of 0. The outcome in the sixth column is an indicator variable equal to 1 if the overall quality of the brief is rated either 'high' or 'very high' from a separate survey question, while 'average', 'low', and 'very low' are coded as 0. We control for wave and strata (country, seniority, and gender) fixed effects. Standard errors are clustered at the individual level (the unit of randomization).

Table S17. Perceived Quality by Trust in AI blog writing, with Controls - Disaggregated

	(1) Title sparked curiosity	(2) Appropriate level of detail	(3) Clear rationale	(4) Visual presentation	(5) Appropriate tone	(6) Adequate length	(7) Easy to understand	(8) Clear proposed recommendations	(9) Relevant proposed recommendations	(10) Sufficiently detailed recommendations
(1) AI-Generated	-0.082	-0.451*	-0.523**	0.044	-0.371*	-0.452*	-0.013	-0.259	-0.110	-0.073
	(0.228)	(0.266)	(0.246)	(0.269)	(0.216)	(0.252)	(0.201)	(0.237)	(0.237)	(0.287)
RI p-value	[.755]	[.105]	[.031]	[.872]	[.061]	[.079]	[.943]	[.304]	[.663]	[.82]
(2) AI-Reported	-0.033	0.155	-0.093	-0.230	-0.114	0.015	0.064	-0.035	-0.209	-0.003
	(0.252)	(0.247)	(0.237)	(0.263)	(0.165)	(0.251)	(0.190)	(0.232)	(0.250)	(0.290)
RI p-value	[.897]	[.536]	[.702]	[.376]	[.483]	[.947]	[.762]	[.877]	[.43]	[.995]
(3) AI-Generated × AI-Reported	0.321	0.179	0.521	0.268	0.205	0.472	-0.038	0.126	0.079	-0.088
	(0.368)	(0.366)	(0.343)	(0.419)	(0.276)	(0.358)	(0.277)	(0.339)	(0.346)	(0.418)
RI p-value (Permuting AI-Generated)	[.431]	[.656]	[.138]	[.5593]	[.442]	[.206]	[.886]	[.734]	[.804]	[.844]
RI p-value (Permuting AI-Reported)	[.389]	[.643]	[.132]	[.496]	[.433]	[.209]	[.905]	[.734]	[.818]	[.828]
(4) Trust in AI blog writing	0.129	0.440**	-0.086	0.548**	-0.085	0.141	0.203	0.076	-0.074	0.435*
	(0.181)	(0.212)	(0.207)	(0.246)	(0.168)	(0.241)	(0.177)	(0.211)	(0.216)	(0.259)
RI p-value	[.436]	[.036]	[.642]	[.023]	[.558]	[.512]	[.224]	[.707]	[.729]	[.075]
(5) AI-Generated × Trust in AI blog writing	-0.230	0.248	0.493	-0.746**	0.141	0.279	-0.167	0.466	0.378	-0.134
	(0.298)	(0.319)	(0.334)	(0.362)	(0.295)	(0.338)	(0.258)	(0.296)	(0.312)	(0.366)
RI p-value (Permuting AI-Generated)	[.446]	[.457]	[.119]	[.032]	[.584]	[.406]	[.505]	[.124]	[.241]	[.729]
RI p-value (Permuting Trust in AI blog writing)	[.398]	[.429]	[.095]	[.048]	[.596]	[.401]	[.497]	[.111]	[.199]	[.734]
(6) AI-Reported × Trust in AI blog writing	-0.059	-0.589*	0.240	0.085	0.176	-0.271	-0.237	-0.056	0.301	-0.352
	(0.312)	(0.301)	(0.294)	(0.365)	(0.229)	(0.326)	(0.249)	(0.301)	(0.323)	(0.376)
RI p-value (Permuting AI-Reported)	[.841]	[.06]	[.413]	[.841]	[.458]	[.415]	[.328]	[.848]	[.38]	[.348]
RI p-value (Permuting Trust in AI blog writing)	[.829]	[.053]	[.361]	[.8]	[.388]	[.332]	[.322]	[.855]	[.402]	[.318]
(7) AI-Generated × AI-Reported × Trust in AI blog writing	0.143	0.190	-0.414	0.366	-0.063	-0.276	0.282	0.001	-0.108	0.750
	(0.459)	(0.448)	(0.441)	(0.522)	(0.360)	(0.466)	(0.361)	(0.442)	(0.443)	(0.522)
RI p-value (Permuting AI-Generated)	[.743]	[.702]	[.336]	[.508]	[.856]	[.548]	[.43]	[.996]	[.818]	[.17]
RI p-value (Permuting AI-Reported)	[.759]	[.679]	[.37]	[.454]	[.866]	[.566]	[.446]	[1]	[.810]	[.143]
RI p-value (Permuting Trust in AI blog writing)	[.702]	[.661]	[.286]	[.465]	[.847]	[.529]	[.409]	[.998]	[.798]	[.125]
Mean of Human-Generated and Human-Reported	3.107	2.929	2.893	2.583	3.083	3.071	3.25	3.012	2.94	2.667
(1) + (5): Effect of AI-Generated, Trust = 1	-0.312*	-0.203	-0.030	-0.702***	-0.231	-0.172	-0.179	0.207	0.268	-0.207
(2) + (6): Effect of AI-Reported, Trust = 1	-0.092	-0.433**	0.147	-0.145	0.062	-0.256	-0.173	-0.092	0.092	-0.355
(3) + (7): Effect of AI-Generated × AI-Reported, Trust = 1	0.464*	0.368	0.107	0.634**	0.141	0.196	0.244	0.127	-0.029	0.662**
(1) + (2) + (3): Total Effect of AI, Trust = 0	0.206	-0.117	-0.095	0.082	-0.280*	0.036	0.013	-0.168	-0.240	-0.164
(1) + (2) + (3) + (5) + (6) + (7): Total Effect of AI, Trust = 1	0.060	-0.268	0.224	-0.213	-0.027	-0.232	-0.109	0.242	0.331*	0.100
(5) + (6) + (7): Difference in Total Effect of AI, Trust = 0 vs. Trust = 1	-0.146	-0.151	0.319	-0.295	0.253	-0.268	-0.122	0.411	0.571*	0.263
Observations	366	366	366	366	366	366	366	366	366	366

Notes: * p<0.1, ** p<0.05, *** p<0.01. This table reports the heterogeneous effects of the treatments on perceptions of the blog's quality by whether the respondent trusts AI to generate high-quality blogs (indicator equal to 1) for each of the 10 quality attributes separately. We include this indicator and interact it with indicators for AI-generated blogs, AI-reported blogs, and their interaction (resulting in a triple interaction). Perceptions are measured using a 5-point Likert scale ranging from 'strongly disagree' that the blog is high-quality with respect to this attribute (0) to 'strongly agree' (4). An indicator variable is assigned a value of 1 if the response is 'agree' or 'strongly agree', while 'neutral', 'disagree', and 'strongly disagree' are assigned a value of 0. We control for respondent characteristics, wave, and strata (country, seniority, and gender) fixed effects. Standard errors are clustered at the individual level (the unit of randomization).

Table S18. Perceived Quality by Trust in AI blog writing, without Controls - Disaggregated

	(1)	(2)	(3)	(4)	(5)	(6)	(7)	(8)	(9)	(10)
	Title sparked curiosity	Appropriate level of detail	Clear rationale	Visual presentation	Appropriate tone	Adequate length	Easy to understand	Clear proposed recommendations	Relevant proposed recommendations	Sufficiently detailed recommendations
(1) AI-Generated	-0.129	-0.354	-0.410*	0.013	-0.335*	-0.364	0.057	-0.203	-0.117	0.028
	(0.226)	(0.264)	(0.240)	(0.262)	(0.200)	(0.254)	(0.199)	(0.240)	(0.238)	(0.271)
RI p-value	[.62]	[.168]	[.09]	[.952]	[.084]	[.142]	[.804]	[.388]	[.649]	[.899]
(2) AI-Reported	-0.020	0.211	-0.039	-0.195	-0.046	0.082	0.122	-0.048	-0.196	0.031
	(0.249)	(0.241)	(0.239)	(0.249)	(0.157)	(0.240)	(0.185)	(0.234)	(0.245)	(0.278)
RI p-value	[.938]	[.395]	[.871]	[.446]	[.759]	[.766]	[.526]	[.833]	[.413]	[.92]
(3) AI-Generated × AI-Reported	0.327	0.079	0.405	0.301	0.169	0.335	-0.077	0.136	0.040	-0.149
	(0.354)	(0.356)	(0.340)	(0.403)	(0.257)	(0.349)	(0.268)	(0.345)	(0.347)	(0.408)
RI p-value (Permuting AI-Generated)	[.435]	[.834]	[.259]	[.48]	[.529]	[.337]	[.78]	[.702]	[.914]	[.717]
RI p-value (Permuting AI-Reported)	[.364]	[.827]	[.234]	[.434]	[.495]	[.381]	[.788]	[.692]	[.91]	[.713]
(4) Trust in AI blog writing	0.177	0.552***	0.020	0.636***	0.013	0.166	0.327**	0.181	0.099	0.523**
	(0.172)	(0.209)	(0.205)	(0.238)	(0.159)	(0.215)	(0.162)	(0.204)	(0.198)	(0.234)
RI p-value	[.297]	[.012]	[.918]	[.011]	[.935]	[.42]	[.057]	[.399]	[.609]	[.022]
(5) AI-Generated × Trust in AI blog writing	-0.178	0.071	0.318	-0.743**	0.092	0.131	-0.379	0.286	0.324	-0.278
	(0.281)	(0.316)	(0.313)	(0.354)	(0.269)	(0.327)	(0.245)	(0.292)	(0.300)	(0.352)
RI p-value (Permuting AI-Generated)	[.561]	[.823]	[.307]	[.034]	[.732]	[.676]	[.111]	[.349]	[.286]	[.414]
RI p-value (Permuting Trust in AI blog writing)	[.532]	[.810]	[.284]	[.035]	[.741]	[.67]	[.101]	[.325]	[.207]	[.441]
(6) AI-Reported × Trust in AI blog writing	-0.132	-0.584**	0.191	0.006	0.049	-0.221	-0.336	-0.097	0.133	-0.410
	(0.307)	(0.295)	(0.292)	(0.347)	(0.222)	(0.305)	(0.239)	(0.295)	(0.315)	(0.353)
RI p-value (Permuting AI-Reported)	[.661]	[.064]	[.508]	[.985]	[.801]	[.493]	[.143]	[.754]	[.686]	[.248]
RI p-value (Permuting Trust in AI blog writing)	[.622]	[.052]	[.459]	[.989]	[.788]	[.405]	[.177]	[.774]	[.707]	[.229]
(7) AI-Generated × AI-Reported × Trust in AI blog writing	0.143	0.207	-0.333	0.311	-0.004	-0.195	0.375	0.029	0.024	0.784
	(0.442)	(0.438)	(0.424)	(0.517)	(0.343)	(0.443)	(0.343)	(0.429)	(0.439)	(0.510)
RI p-value (Permuting AI-Generated)	[.744]	[.652]	[.451]	[.558]	[.996]	[.656]	[.272]	[.943]	[.972]	[.126]
RI p-value (Permuting AI-Reported)	[.735]	[.643]	[.459]	[.55]	[.991]	[.662]	[.279]	[.948]	[.954]	[.132]
RI p-value (Permuting Trust in AI blog writing)	[.694]	[.607]	[.396]	[.533]	[.99]	[.669]	[.279]	[.95]	[.952]	[.106]
Mean of Human-Generated and Human-Reported	3.107	2.929	2.893	2.583	3.083	3.071	3.25	3.012	2.94	2.667
(1) + (5): Effect of AI-Generated, Trust = 1	-0.308**	-0.283*	-0.092	-0.730***	-0.243	-0.233	-0.322**	0.084	0.207	-0.250
(2) + (6): Effect of AI-Reported, Trust = 1	-0.152	-0.373**	0.152	-0.189	0.004	-0.139	-0.214	-0.145	-0.063	-0.379*
(3) + (7): Effect of AI-Generated × AI-Reported, Trust = 1	0.470**	0.286	0.072	0.611**	0.165	0.139	0.298	0.165	0.064	0.635**
(1) + (2) + (3): Total Effect of AI, Trust = 0	0.178	-0.064	-0.044	0.118	-0.212	0.053	0.102	-0.114	-0.272	-0.090
(1) + (2) + (3) + (5) + (6) + (7): Total Effect of AI, Trust = 1	0.010	-0.370**	0.131	-0.308	-0.074	-0.232	-0.238	0.104	0.209	0.006
(5) + (6) + (7): Difference in Total Effect of AI, Trust = 0 vs. Trust = 1	-0.167	-0.305	0.176	-0.426	0.138	-0.285	-0.340	0.218	0.482*	0.096
Observations	366	366	366	366	366	366	366	366	366	366

Notes: * p<0.1, ** p<0.05, *** p<0.01. This table reports the heterogeneous effects of the treatments on perceptions of the blog's quality by whether the respondent trusts AI to write high quality blogs (indicator equal to 1) for each of the 10 quality attributes separately. We include this indicator and interact it with indicators for AI-generated blogs, AI-reported blogs, and their interaction (resulting in a triple interaction). Perceptions are measured using a 5-point Likert scale ranging from 'strongly disagree' that the blog is high-quality with respect to this attribute (0) to 'strongly agree' (4). An indicator variable is assigned a value of 1 if the response is 'agree' or 'strongly agree', while 'neutral', 'disagree', and 'strongly disagree' are assigned a value of 0. We control for wave and strata (country, seniority, and gender) fixed effects. Standard errors are clustered at the individual level (the unit of randomization).

Table S19. Perceived Quality by Gender, with Controls

	(1)	(2)	(3)	(4)	(5)	(6)
	Unweighted Average Quality (0-4) (10 Attributes)	Weighted Average Quality (0-4) (9 Attributes)	Unweighted average quality (0-4) (10 Attributes)	Standardized First Principal Component (10 Attributes)	Number of 'agree' or 'strongly agree' statements (0-10)	Overall quality of brief rated 'high' or 'very high'
(1) AI-Generated	-0.211*	-0.203	-0.200	-0.298*	-1.125**	-0.174*
	(0.121)	(0.123)	(0.123)	(0.176)	(0.520)	(0.089)
RI p-value	[.082]	[.102]	[.11]	[.089]	[.037]	[.035]
(2) AI-Reported	0.003	0.008	-0.000	0.009	-0.342	-0.095
	(0.120)	(0.123)	(0.123)	(0.177)	(0.540)	(0.090)
RI p-value	[.985]	[.928]	[.996]	[.964]	[.52]	[.292]
(3) AI-Generated × AI-Reported	0.129	0.119	0.128	0.174	0.831	0.155
	(0.166)	(0.170)	(0.170)	(0.244)	(0.742)	(0.128)
RI p-value (Permuting AI-Generated)	[.45]	[.51]	[.47]	[.493]	[.271]	[.207]
RI p-value (Permuting AI-Reported)	[.474]	[.502]	[.484]	[.495]	[.289]	[.216]
(4) Female	-0.463	-0.541*	-0.486	-0.580	-2.437*	-0.031
	(0.302)	(0.317)	(0.314)	(0.441)	(1.445)	(0.237)
(5) AI-Generated × Female	0.120	0.150	0.123	0.230	0.564	0.049
	(0.228)	(0.236)	(0.232)	(0.339)	(0.951)	(0.168)
RI p-value (Permuting AI-Generated)	[.611]	[.535]	[.61]	[.509]	[.567]	[.763]
(6) AI-Reported × Female	-0.352*	-0.356*	-0.370*	-0.525*	-0.718	-0.093
	(0.199)	(0.205)	(0.206)	(0.291)	(0.931)	(0.161)
RI p-value (Permuting AI-Reported)	[.07]	[.072]	[.067]	[.069]	[.409]	[.572]
(7) AI-Generated × AI-Reported × Female	0.557*	0.580*	0.600*	0.715	1.922	0.325
	(0.307)	(0.319)	(0.315)	(0.453)	(1.338)	(0.232)
RI p-value (Permuting AI-Generated)	[.14]	[.141]	[.129]	[.195]	[.219]	[.214]
RI p-value (Permuting AI-Reported)	[.087]	[.089]	[.072]	[.129]	[.173]	[.21]
Mean of Human-Generated and Human-Reported	2.954	2.943	2.939	.141	7.75	.702
(1) + (5): Effect of AI-Generated, Female = 1	-0.091	-0.053	-0.077	-0.068	-0.561	-0.124
(2) + (6): Effect of AI-Reported, Female = 1	-0.349**	-0.348**	-0.370**	-0.515**	-1.060	-0.188
(3) + (7): Effect of AI-Generated × AI-Reported, Female = 1	0.686***	0.699***	0.728***	0.889**	2.753**	0.480**
(1) + (2) + (3): Total Effect of AI, Female = 0	-0.080	-0.075	-0.072	-0.114	-0.636	-0.115
(1) + (2) + (3) + (5) + (6) + (7): Total Effect of AI, Female = 1	0.246	0.298	0.281	0.306	1.132	0.167
(5) + (6) + (7): Difference in Total Effect of AI, Female = 0 vs. Female = 1	0.326	0.373	0.353	0.420	1.768*	0.282*
Observations	366	366	366	366	366	366

Notes: * p<0.1, ** p<0.05, *** p<0.01. This table reports the heterogeneous effects of the treatments on perceptions of the blog's quality by whether the respondent is female (indicator equal to 1). We include this indicator as well as interact it with indicators for AI-generated blogs, AI-reported blogs, and their interaction (resulting in a triple interaction). Perceptions are measured using a 5-point Likert scale ranging from 'strongly disagree' that the blog is high-quality with respect to this attribute (0) to 'strongly agree' (4). The 10 attributes are: blog had an appropriate amount of detail, a clear rationale, a catchy title, good visual presentation, an appropriate tone, was of adequate length, was easy to understand, and provided clear, relevant, and sufficiently detailed recommendations. The outcome in the first column is the unweighted average across the 10 attributes, and the outcome in the second column is the weighted average across nine attributes (weighted by respondents' reported relative importance of these attributes save appropriate tone). The outcome in the third column is the unweighted average of the nine statements, as a robustness check. The outcome in the fourth column is the standardized score of the first principal component, calculated based on the same 10 attributes using Polychoric PCA. The outcome in the fifth column is the total number of the 10 attributes rated as either 'high' or 'very high' from a separate survey question, while 'average', 'low', and 'very low' are coded as 0. The outcome in the sixth column is an indicator variable equal to 1 if the overall quality of the brief is rated either 'high' or 'very high' from a separate survey question, while 'average', 'low', and 'very low' are coded as 0. We control for respondent characteristics, wave, and strata (country, seniority, and gender) fixed effects. Note that gender cannot be permuted since it is a component of the strata. Standard errors are clustered at the individual level (the unit of randomization).

Table S20. Perceived Quality by Gender, without Controls

	(1)	(2)	(3)	(4)	(5)	(6)
	Unweighted Average Quality (0-4) (10 Attributes)	Weighted Average Quality (0-4) (9 Attributes)	Unweighted average quality (0-4) (10 Attributes)	Standardized First Principal Component (10 Attributes)	Number of 'agree' and 'strongly agree' statements (0-10)	Overall quality of brief rated 'high' or 'very high'
(1) AI-Generated	-0.245**	-0.237*	-0.239*	-0.340*	-1.153**	-0.187**
	(0.123)	(0.126)	(0.126)	(0.175)	(0.537)	(0.085)
RI p-value	[.043]	[.065]	[.049]	[.048]	[.04]	[.027]
(2) AI-Reported	-0.005	0.003	-0.008	0.007	-0.360	-0.101
	(0.119)	(0.122)	(0.123)	(0.176)	(0.522)	(0.090)
RI p-value	[.966]	[.982]	[.948]	[.962]	[.46]	[.226]
(3) AI-Generated × AI-Reported	0.128	0.113	0.127	0.156	0.777	0.162
	(0.167)	(0.172)	(0.172)	(0.245)	(0.739)	(0.126)
RI p-value (Permuting AI-Generated)	[.428]	[.501]	[.446]	[.51]	[.285]	[.192]
RI p-value (Permuting AI-Reported)	[.448]	[.511]	[.476]	[.539]	[.274]	[.207]
(4) Female	-0.517*	-0.581**	-0.539*	-0.685*	-2.540**	-0.105
	(0.271)	(0.290)	(0.285)	(0.395)	(1.272)	(0.207)
(5) AI-Generated × Female	0.205	0.229	0.218	0.336	0.827	0.079
	(0.209)	(0.217)	(0.214)	(0.312)	(0.896)	(0.157)
RI p-value (Permuting AI-Generated)	[.375]	[.346]	[.36]	[.363]	[.369]	[.615]
(6) AI-Reported × Female	-0.319	-0.327	-0.338*	-0.474*	-0.572	-0.035
	(0.195)	(0.202)	(0.203)	(0.288)	(0.926)	(0.158)
RI p-value (Permuting AI-Reported)	[.092]	[.114]	[.09]	[.096]	[.549]	[.817]
(7) AI-Generated × AI-Reported × Female	0.456	0.480	0.489*	0.589	1.376	0.230
	(0.287)	(0.299)	(0.297)	(0.426)	(1.245)	(0.221)
RI p-value (Permuting AI-Generated)	[.207]	[.223]	[.208]	[.31]	[.354]	[.338]
RI p-value (Permuting AI-Reported)	[.137]	[.109]	[.112]	[.178]	[.268]	[.353]
Mean of Human-Generated and Human-Reported	2.954	2.943	2.939	.141	7.75	.702
(1) + (5): Effect of AI-Generated, Female = 1	-0.041	-0.008	-0.021	-0.003	-0.326	-0.107
(2) + (6): Effect of AI-Reported, Female = 1	-0.324**	-0.324**	-0.346**	-0.468**	-0.932	-0.137
(3) + (7): Effect of AI-Generated × AI-Reported, Female = 1	0.584**	0.593**	0.617**	0.745**	2.154**	0.392**
(1) + (2) + (3): Total Effect of AI, Female = 0	-0.122	-0.121	-0.119	-0.177	-0.736	-0.126
(1) + (2) + (3) + (5) + (6) + (7): Total Effect of AI, Female = 1	0.219	0.261	0.249	0.274	0.895	0.148
(5) + (6) + (7): Difference in Total Effect of AI, Female = 0 vs. Female = 1	0.341	0.382*	0.369*	0.451	1.631*	0.274*
Observations	366	366	366	366	366	366

Notes: * p<0.1, ** p<0.05, *** p<0.01. This table reports the heterogeneous effects of the treatments on perceptions of the blog's quality by whether the respondent is female (indicator equal to 1). We include this indicator as well as interact it with indicators for AI-generated blogs, AI-reported blogs, and their interaction (resulting in a triple interaction). Perceptions are measured using a 5-point Likert scale ranging from 'strongly disagree' that the blog is high-quality with respect to this attribute (0) to 'strongly agree' (4). The 10 attributes are: blog had an appropriate amount of detail, a clear rationale, a catchy title, good visual presentation, an appropriate tone, was of adequate length, was easy to understand, and provided clear, relevant, and sufficiently detailed recommendations. The outcome in the first column is the unweighted average across the 10 attributes, and the outcome in the second column is the weighted average across nine attributes (weighted by respondents' reported relative importance of these attributes save appropriate tone). The outcome in the third column is the unweighted average of the nine statements, as a robustness check. The outcome in the fourth column is the standardized score of the first principal component, calculated based on the same 10 attributes using Polychoric PCA. The outcome in the fifth column is the total number of the 10 attributes rated as either 'agree' or 'strongly agree' while 'neutral', 'disagree', and 'strongly disagree' are coded as 0. The outcome in the sixth column is an indicator variable equal to 1 if the overall quality of the brief is rated either 'high' or 'very high' from a separate survey question, while 'average', 'low', and 'very low' are coded as 0. We control for wave and strata (country, seniority, and gender) fixed effects. Note that gender cannot be permuted since it is a component of the strata. Standard errors are clustered at the individual level (the unit of randomization).

Table S21. Perceived Quality by Gender, with Controls - Disaggregated

	(1)	(2)	(3)	(4)	(5)	(6)	(7)	(8)	(9)	(10)
	Title sparked curiosity	Appropriate level of detail	Clear rationale	Visual presentation	Appropriate tone	Adequate length	Easy to understand	Clear proposed recommendations	Relevant proposed recommendations	Sufficiently detailed recommendations
(1) AI-Generated	-0.285*	-0.261	-0.279*	-0.389*	-0.317**	-0.275	-0.110	-0.025	-0.021	-0.152
	(0.156)	(0.183)	(0.161)	(0.216)	(0.150)	(0.195)	(0.141)	(0.167)	(0.157)	(0.200)
RI p-value	[.07]	[.174]	[.1]	[.059]	[.039]	[.152]	[.419]	[.874]	[.885]	[.459]
(2) AI-Reported	-0.061	-0.119	0.136	-0.141	0.035	-0.012	0.077	0.110	0.072	-0.064
	(0.162)	(0.174)	(0.156)	(0.211)	(0.131)	(0.185)	(0.133)	(0.163)	(0.169)	(0.202)
RI p-value	[.719]	[.483]	[.376]	[.517]	[.792]	[.948]	[.555]	[.462]	[.653]	[.754]
(3) AI-Generated × AI-Reported	0.256	0.180	0.133	0.241	0.132	0.194	0.012	0.023	-0.077	0.191
	(0.232)	(0.254)	(0.221)	(0.292)	(0.189)	(0.257)	(0.188)	(0.229)	(0.223)	(0.275)
RI p-value (Permuting AI-Generated)	[.239]	[.5]	[.554]	[.377]	[.493]	[.487]	[.958]	[.934]	[.739]	[.507]
RI p-value (Permuting AI-Reported)	[.294]	[.53]	[.565]	[.41]	[.508]	[.456]	[.936]	[.916]	[.741]	[.488]
(4) Female	-0.593*	-0.498	-1.012**	-1.050**	-0.255	0.332	0.263	-0.461	-0.771*	-0.586
	(0.344)	(0.444)	(0.443)	(0.443)	(0.342)	(0.401)	(0.431)	(0.488)	(0.463)	(0.532)
(5) AI-Generated × Female	0.215	-0.073	0.161	0.038	0.100	-0.033	0.022	0.193	0.487*	0.095
	(0.269)	(0.368)	(0.356)	(0.388)	(0.299)	(0.343)	(0.288)	(0.324)	(0.290)	(0.399)
RI p-value (Permuting AI-Generated)	[.482]	[.863]	[.637]	[.934]	[.731]	[.917]	[.939]	[.568]	[.112]	[.813]
(6) AI-Reported × Female	-0.057	-0.167	-0.343	0.045	-0.192	-0.543*	-0.635**	-0.706**	-0.439	-0.482
	(0.313)	(0.296)	(0.301)	(0.337)	(0.246)	(0.313)	(0.281)	(0.325)	(0.328)	(0.368)
RI p-value (Permuting AI-Reported)	[.865]	[.583]	[.22]	[.897]	[.46]	[.105]	[.039]	[.035]	[.189]	[.163]
(7) AI-Generated × AI-Reported × Female	0.680	0.439	0.625	0.909*	0.176	0.484	0.533	0.503	0.492	0.732
	(0.422)	(0.481)	(0.454)	(0.514)	(0.378)	(0.471)	(0.360)	(0.447)	(0.432)	(0.564)
RI p-value (Permuting AI-Generated)	[.197]	[.465]	[.192]	[.172]	[.66]	[.393]	[.211]	[.301]	[.339]	[.265]
RI p-value (Permuting AI-Reported)	[.177]	[.388]	[.177]	[.14]	[.668]	[.357]	[.178]	[.249]	[.254]	[.216]
Mean of Human-Generated and Human-Reported	3.107	2.929	2.893	2.583	3.083	3.071	3.25	3.012	2.94	2.667
(1) + (5): Effect of AI-Generated, Female = 1	-0.070	-0.334	-0.119	-0.350	-0.217	-0.307	-0.088	0.168	0.465**	-0.057
(2) + (6): Effect of AI-Reported, Female = 1	-0.118	-0.286	-0.207	-0.096	-0.157	-0.555**	-0.558**	-0.595**	-0.367	-0.546*
(3) + (7): Effect of AI-Generated × AI-Reported, Female = 1	0.936***	0.619	0.759*	1.150***	0.309	0.678*	0.545*	0.526	0.414	0.923*
(1) + (2) + (3): Total Effect of AI, Female = 0	-0.090	-0.200	-0.010	-0.289	-0.150	-0.093	-0.021	0.108	-0.027	-0.025
(1) + (2) + (3) + (5) + (6) + (7): Total Effect of AI, Female = 1	0.748***	-0.002	0.433*	0.703**	-0.065	-0.185	-0.101	0.099	0.513**	0.320
(5) + (6) + (7): Difference in Total Effect of AI, Female = 0 vs. Female = 1	0.837***	0.199	0.443	0.993***	0.085	-0.092	-0.080	-0.010	0.539*	0.345
Observations	366	366	366	366	366	366	366	366	366	366

Notes: * p<0.1, ** p<0.05, *** p<0.01. This table reports the heterogeneous effects of the treatments on perceptions of the blog's quality by whether the respondent is female (indicator equal to 1) for each of the 10 quality attributes separately. We include this indicator and interact it with indicators for AI-generated blogs, AI-reported blogs, and their interaction (resulting in a triple interaction). Perceptions are measured using a 5-point Likert scale ranging from 'strongly disagree' that the blog is high-quality with respect to this attribute (0) to 'strongly agree' (4). An indicator variable is assigned a value of 1 if the response is either 'agree' or 'strongly agree' while 'neutral', 'disagree', and 'strongly' are coded as 0. We control for respondent characteristics, wave, and strata (country, seniority, and gender) fixed effects. Note that gender cannot be permuted since it is a component of the strata. Standard errors are clustered at the individual level (the unit of randomization).

Table S22. Perceived Quality by Gender, without Controls - Disaggregated

	(1)	(2)	(3)	(4)	(5)	(6)	(7)	(8)	(9)	(10)
	Title sparked curiosity	Appropriate level of detail	Clear rationale	Visual presentation	Appropriate tone	Adequate length	Easy to understand	Clear proposed recommendations	Relevant proposed recommendations	Sufficiently detailed recommendations
(1) AI-Generated	-0.334**	-0.307*	-0.283*	-0.466**	-0.303**	-0.298	-0.158	-0.082	-0.049	-0.171
	(0.156)	(0.184)	(0.164)	(0.207)	(0.148)	(0.195)	(0.138)	(0.165)	(0.162)	(0.195)
RI p-value	[.026]	[.083]	[.092]	[.017]	[.043]	[.109]	[.235]	[.599]	[.782]	[.383]
(2) AI-Reported	-0.104	-0.081	0.156	-0.146	0.022	0.060	0.073	0.062	-0.011	-0.082
	(0.154)	(0.178)	(0.152)	(0.212)	(0.127)	(0.179)	(0.131)	(0.161)	(0.168)	(0.201)
RI p-value	[.503]	[.642]	[.285]	[.459]	[.863]	[.713]	[.572]	[.692]	[.938]	[.693]
(3) AI-Generated × AI-Reported	0.306	0.114	0.086	0.274	0.135	0.140	0.002	0.040	-0.006	0.189
	(0.224)	(0.251)	(0.219)	(0.291)	(0.191)	(0.259)	(0.192)	(0.230)	(0.226)	(0.272)
RI p-value (Permuting AI-Generated)	[.159]	[.664]	[.699]	[.329]	[.493]	[.602]	[.991]	[.863]	[.977]	[.469]
RI p-value (Permuting AI-Reported)	[.19]	[.646]	[.687]	[.364]	[.496]	[.577]	[.991]	[.873]	[.975]	[.502]
(4) Female	-0.803**	-0.593	-1.013**	-1.187***	-0.319	0.235	0.158	-0.497	-0.576	-0.574
	(0.338)	(0.459)	(0.444)	(0.420)	(0.301)	(0.354)	(0.391)	(0.469)	(0.464)	(0.515)
(5) AI-Generated × Female	0.378	0.079	0.224	0.228	0.086	0.083	0.034	0.229	0.479*	0.225
	(0.262)	(0.342)	(0.341)	(0.352)	(0.278)	(0.297)	(0.253)	(0.304)	(0.281)	(0.367)
RI p-value (Permuting AI-Generated)	[.204]	[.84]	[.535]	[.585]	[.763]	[.821]	[.898]	[.484]	[.108]	[.555]
(6) AI-Reported × Female	-0.019	-0.146	-0.326	0.011	-0.148	-0.465	-0.599**	-0.664**	-0.402	-0.436
	(0.307)	(0.291)	(0.311)	(0.340)	(0.236)	(0.298)	(0.273)	(0.325)	(0.329)	(0.360)
RI p-value (Permuting AI-Reported)	[.951]	[.63]	[.273]	[.972]	[.512]	[.117]	[.035]	[.034]	[.207]	[.228]
(7) AI-Generated × AI-Reported × Female	0.497	0.339	0.523	0.725	0.151	0.321	0.581*	0.522	0.340	0.558
	(0.406)	(0.458)	(0.440)	(0.488)	(0.357)	(0.431)	(0.344)	(0.430)	(0.428)	(0.532)
RI p-value (Permuting AI-Generated)	[.322]	[.567]	[.313]	[.251]	[.686]	[.561]	[.149]	[.282]	[.525]	[.368]
RI p-value (Permuting AI-Reported)	[.322]	[.509]	[.264]	[.219]	[.71]	[.484]	[.12]	[.207]	[.447]	[.299]
Mean of Human-Generated and Human-Reported	3.107	2.929	2.893	2.583	3.083	3.071	3.25	3.012	2.94	2.667
(1) + (5): Effect of AI-Generated, Female = 1	0.044	-0.228	-0.058	-0.238	-0.217	-0.215	-0.124	0.147	0.430*	0.054
(2) + (6): Effect of AI-Reported, Female = 1	-0.123	-0.227	-0.170	-0.135	-0.126	-0.404*	-0.526**	-0.601**	-0.414	-0.517*
(3) + (7): Effect of AI-Generated × AI-Reported, Female = 1	0.803**	0.452	0.608	0.998**	0.286	0.461	0.583**	0.562	0.334	0.747
(1) + (2) + (3): Total Effect of AI, Female = 0	-0.132	-0.274	-0.041	-0.338*	-0.146	-0.097	-0.083	0.020	-0.066	-0.063
(1) + (2) + (3) + (5) + (6) + (7): Total Effect of AI, Female = 1	0.724***	-0.003	0.380	0.625**	-0.057	-0.158	-0.066	0.107	0.350	0.284
(5) + (6) + (7): Difference in Total Effect of AI, Female = 0 vs. Female = 1	0.856***	0.272	0.421	0.964***	0.089	-0.061	0.017	0.087	0.417	0.347
Observations	366	366	366	366	366	366	366	366	366	366

Notes: * p<0.1, ** p<0.05, *** p<0.01. This table reports the heterogeneous effects of the treatments on perceptions of the blog's quality by whether the respondent is female (indicator equal to 1) for each of the 10 quality attributes separately. We include this indicator and interact it with indicators for AI-generated blogs, AI-reported blogs, and their interaction (resulting in a triple interaction). Perceptions are measured using a 5-point Likert scale ranging from 'strongly disagree' that the blog is high-quality with respect to this attribute (0) to 'strongly agree' (4). An indicator variable is assigned a value of 1 if the response is either 'agree' or 'strongly agree' while 'neutral', 'disagree', and 'highly disagree' are coded as 0. We control for wave and strata (country, seniority, and gender) fixed effects. Note that gender cannot be permuted since it is a component of the strata. Standard errors are clustered at the individual level (the unit of randomization).

Table S23. Intended in Engagement with the Blog, with Controls

	(1)	(2)	(3)	(4)
	Average engagement rating (0-4)	Standardized First Principal Component	Number of 'likely' or 'very likely' statements (0-5)	Log time spent reading the blog (minutes)
(1) AI-Generated	-0.155	-0.157	-0.325	-0.027
	(0.128)	(0.151)	(0.252)	(0.217)
RI p-value	[.216]	[.308]	[.199]	[.885]
(2) AI-Reported	0.038	0.071	-0.016	0.194
	(0.134)	(0.158)	(0.270)	(0.248)
RI p-value	[.773]	[.675]	[.956]	[.424]
(3) AI-Generated × AI-Reported	0.048	0.024	0.113	-0.091
	0.182	(0.213)	(0.366)	(0.316)
RI p-value (Permuting AI-Generated)	[.786]	[.916]	[.775]	[.793]
RI p-value (Permuting AI-Reported)	[.806]	[.907]	[.784]	[.779]
Mean of Human-Generated and Human-Reported	2.729	.085	3.25	.52
(1) + (3): Total Effect of AI-Generated	-0.107	-0.133	-0.212	-0.118
(1) + (2) + (3): Total Effect of AI	-0.069	-0.062	-0.228	0.076
Observations	366	366	366	365

Notes: * p<0.1, ** p<0.05, *** p<0.01. This table reports the effects of the treatments on respondents' reported intended engagement with the blog across five actions. Each action is measured using a 5-point Likert scale ranging from 'very unlikely' that the respondent will take that action (0) to 'very likely' (4). The actions are: whether they would share the blog with others, re-read the blog, look up studies cited in the blog, look up related studies, and contact the authors. The outcome in the first column is the unweighted average across the five actions. The outcome in the second column is the standardized score of the first principal component, calculated based on the same five attributes using Polychoric PCA. The outcome in the third column is the total number of the five actions rated as either 'likely' or 'very likely' while 'neutral', 'unlikely', and 'very unlikely' are assigned a value of 0. The outcome in the fourth column is the log number of minutes the respondent spent reading the blog. We control for respondent characteristics, wave, and strata (country, seniority, and gender) fixed effects. Standard errors are clustered at the individual level (the unit of randomization).

Table S24. Intended Engagement with the Blog, without Controls

	(1)	(2)	(3)	(4)
	Average engagement rating (0-4)	Standardized First Principal Component	Number of 'likely' or 'very likely' statements (0-5)	Log time spent reading the blog (minutes)
(1) AI-Generated	-0.165	-0.175	-0.276	-0.124
	(0.126)	(0.148)	(0.254)	(0.212)
RI p-value	[.194]	[.214]	[.285]	[.560]
(2) AI-Reported	0.002	0.021	-0.013	0.096
	(0.125)	(0.147)	(0.261)	(0.238)
RI p-value	[.989]	[.877]	[.96]	[.698]
(3) AI-Generated × AI-Reported	0.057	0.045	0.039	0.066
	(0.177)	(0.207)	(0.362)	(0.301)
RI p-value (Permuting AI-Generated)	[.765]	[.815]	[.908]	[.83]
RI p-value (Permuting AI-Reported)	[.765]	[.827]	[.918]	[.83]
Mean of Human-Generated and Human-Reported	2.729	.085	3.25	.52
(1) + (3): Total Effect of AI-Generated	-0.108	-0.130	-0.237	-0.058
(1) + (2) + (3): Total Effect of AI	-0.106	-0.108	-0.250	0.038
Observations	366	366	366	365

Notes: * p<0.1, ** p<0.05, *** p<0.01. This table reports the effects of the treatments on respondents' reported intended engagement with the blog across five actions. Each action is measured using a 5-point Likert scale ranging from 'very unlikely' that the respondent will take that action (0) to 'very likely' (4). The actions are: whether they would share the blog with others, re-read the blog, look up studies cited in the blog, look up related studies, and contact the authors. The outcome in the first column is the unweighted average across the five actions. The outcome in the second column is the standardized score of the first principal component, calculated based on the same five attributes using Polychoric PCA. The outcome in the third column is the total number of the five actions rated as either 'likely' or 'very likely' while 'neutral', 'unlikely', and 'very unlikely' are assigned a value of 0. The outcome in the fourth column is the log number of minutes the respondent spent reading the blog. We control for wave and strata (country, seniority, and gender) fixed effects. Standard errors are clustered at the individual level (the unit of randomization).

Table S25. Intended Engagement with the Blog, with Controls - Disaggregated

	(1)	(2)	(3)	(4)	(5)
	Share the blog with others	Re-read the blog	Look up studies cited	Look up related studies	Contact the authors of the brief
(1) AI-Generated	-0.280	-0.240	-0.148	-0.047	-0.061
	(0.174)	(0.175)	(0.159)	(0.147)	(0.188)
RI p-value	[.122]	[.203]	[.352]	[.755]	[.742]
(2) AI-Reported	-0.155	0.012	0.224	0.059	0.052
	(0.176)	(0.180)	(0.167)	(0.157)	(0.197)
RI p-value	[.328]	[.944]	[.172]	[.696]	[.787]
(3) AI-Generated × AI-Reported	0.214	0.151	-0.129	0.075	-0.071
	(0.245)	(0.249)	(0.228)	(0.206)	(0.266)
RI p-value (Permuting AI-Generated)	[.364]	[.574]	[.569]	[.744]	[.808]
RI p-value (Permuting AI-Reported)	[.403]	[.577]	[.573]	[.731]	[.757]
Mean of Human-Generated and Human-Reported	2.929	2.833	2.81	2.798	2.274
(1) + (3): Total Effect of AI-Generated	-0.066	-0.089	-0.277*	0.028	-0.132
(1) + (2) + (3): Total Effect of AI	-0.221	-0.077	-0.052	0.087	-0.080
Observations	366	366	366	366	366

Notes: * p<0.1, ** p<0.05, *** p<0.01. This table reports the effects of the treatments on respondents' reported intended engagement with the blog across the five actions separately. Each action is measured using a 5-point Likert scale ranging from 'very unlikely' that the respondent will take that action (0) to 'very likely' (4). An indicator variable is assigned a value of 1 if the response is either 'likely' or 'very likely' while 'neutral', 'unlikely', and 'very unlikely' are assigned a value of 0. We control for respondent characteristics, wave, and strata (country, seniority, and gender) fixed effects. Standard errors are clustered at the individual level (the unit of randomization).

Table S26. Intended Engagement with the Blog, without Controls - Disaggregated

	(1)	(2)	(3)	(4)	(5)
	Share the blog with others	Re-read the blog	Look up studies cited	Look up related studies	Contact the authors of the brief
(1) AI-Generated	-0.304*	-0.200	-0.173	-0.069	-0.079
	(0.166)	(0.169)	(0.156)	(0.147)	(0.189)
RI p-value	[.077]	[.274]	[.26]	[.639]	[.689]
(2) AI-Reported	-0.120	0.059	0.144	0.027	-0.098
	(0.159)	(0.170)	(0.158)	(0.149)	(0.190)
RI p-value	[.479]	[.717]	[.368]	[.847]	[.586]
(3) AI-Generated × AI-Reported	0.169	0.064	-0.077	0.098	0.031
	(0.237)	(0.239)	(0.219)	(0.203)	(0.261)
RI p-value (Permuting AI-Generated)	[.49]	[.768]	[.729]	[.631]	[.904]
RI p-value (Permuting AI-Reported)	[.527]	[.789]	[.741]	[.628]	[.908]
Mean of Human-Generated and Human-Reported	2.929	2.833	2.81	2.798	2.274
(1) + (3): Total Effect of AI-Generated	-0.135	-0.136	-0.250	0.029	-0.047
(1) + (2) + (3): Total Effect of AI	-0.255	-0.078	-0.106	0.056	-0.145
Observations	366	366	366	366	366

Notes: * $p<0.1$, ** $p<0.05$, *** $p<0.01$. This table reports the effects of the treatments on respondents' reported intended engagement with the blog across the five actions separately. Each action is measured using a 5-point Likert scale ranging from 'very unlikely' that the respondent will take that action (0) to 'very likely' (4). An indicator variable is assigned a value of 1 if the response is either 'likely' or 'very likely' while 'neutral', 'unlikely', and 'very unlikely' are assigned a value of 0. We control for wave and strata (country, seniority, and gender) fixed effects. Standard errors are clustered at the individual level (the unit of randomization).

Table S27. Beliefs in Others Intended Engagement with the Blog, with Controls

	(1)	(2)	(3)	(4)
	Others' Average Engagement Rating (0-4)	Standardized First Principal Component	Number of 'likely' or 'very likely' statements (0-5)	Others' overall rating 'high' or 'very high'
(1) AI-Generated	0.019	0.034	-0.025	-0.106
	(0.124)	(0.149)	(0.280)	(0.079)
RI p-value	[.875]	[.8]	[.927]	[.196]
(2) AI-Reported	-0.000	0.000	0.060	-0.038
	(0.138)	(0.165)	(0.304)	(0.078)
RI p-value	[.997]	[1]	[.846]	[.628]
(3) AI-Generated × AI-Reported	-0.066	-0.087	-0.145	0.048
	(0.179)	(0.212)	(0.398)	(0.113)
RI p-value (Permuting AI-Generated)	[.71]	[.676]	[.735]	[.671]
RI p-value (Permuting AI-Reported)	[.738]	[.687]	[.73]	[.648]
Mean of Human-Generated and Human-Reported	2.586	.063	2.917	.679
(1) + (3): Total Effect of AI-Generated	-0.047	-0.053	-0.170	-0.058
(1) + (2) + (3): Total Effect of AI	-0.047	-0.053	-0.110	-0.096
Observations	366	366	366	366

Notes: * p<0.1, ** p<0.05, *** p<0.01. This table reports the effects of the treatments on respondents' beliefs about others' potential engagement with the blog across the five actions. Each action is measured using a 5-point Likert scale ranging from 'very unlikely' that others will take the action (0) to 'very likely' (4). The actions were: whether they would share the blog with others, re-read the blog, look up studies cited in the blog, look up related studies, and contact the authors. The outcome in the first column is the simple average across the five actions. The outcome in the second column is the standardized score of the first principal component, calculated based on the same five attributes using Polychoric PCA. The outcome in the third column is the total number of the five actions rated as either 'likely' or 'very likely' while 'neutral', 'unlikely', and 'very unlikely' are assigned a value of 0. The outcome in the fourth column is the belief about others' overall rating and is an indicator equal to one for either 'high' or 'very high', while 'average', 'low', and 'very low' are assigned a value of 0. We control for respondent characteristics, wave, and strata (country, seniority, and gender) fixed effects. Standard errors are clustered at the individual level (the unit of randomization).

Table S28. Beliefs in Others Intended Engagement with the Blog, without Controls

	(1)	(2)	(3)	(4)
	Others' Average Engagement Rating (0-4)	Standardized First Principal Component	Number of 'likely' or 'very likely' statements (0-5)	Others' overall rating 'high' or 'very high'
(1) AI-Generated	-0.011	-0.004	-0.037	-0.121
	(0.124)	(0.149)	(0.283)	(0.076)
RI p-value	[.925]	[.978]	[.9]	[.132]
(2) AI-Reported	-0.038	-0.046	-0.031	-0.032
	(0.128)	(0.152)	(0.293)	(0.076)
RI p-value	[.773]	[.756]	[.904]	[.648]
(3) AI-Generated × AI-Reported	-0.046	-0.063	-0.132	0.030
	(0.179)	(0.213)	(0.401)	(0.108)
RI p-value (Permuting AI-Generated)	[.789]	[.769]	[.747]	[.799]
RI p-value (Permuting AI-Reported)	[.818]	[.768]	[.752]	[.773]
Mean of Human-Generated and Human-Reported	2.586	.063	2.917	.679
(1) + (3): Total Effect of AI-Generated	-0.057	-0.067	-0.169	-0.091
(1) + (2) + (3): Total Effect of AI	-0.095	-0.113	-0.200	-0.123
Observations	366	366	366	366

Notes: * $p<0.1$, ** $p<0.05$, *** $p<0.01$. This table reports the effects of the treatments on respondents' beliefs about others' potential engagement with the blog across the five actions. Each action is measured using a 5-point Likert scale ranging from 'very unlikely' that others will take the action (0) to 'very likely' (4). The actions were: whether they would share the blog with others, re-read the blog, look up studies cited in the blog, look up related studies, and contact the authors. The outcome in the first column is the simple average across the five actions. The outcome in the second column is the standardized score of the first principal component, calculated based on the same five attributes using Polychoric PCA. The outcome in the third column is the total number of the five actions rated as either 'likely' or 'very likely' while 'neutral', 'unlikely', and 'very unlikely' are assigned a value of 0. The outcome in the fourth column is the belief about others' overall rating and is an indicator equal to one for either 'high' or 'very high', while 'average', 'low', and 'very low' are assigned a value of 0. We control for wave and strata (country, seniority, and gender) fixed effects. Standard errors are clustered at the individual level (the unit of randomization).

Table S29. Beliefs in Others' Intended Engagement with the Blog, with Controls - Disaggregated

	(1)	(2)	(3)	(4)	(5)
	Share the blog with others	Re-read the blog	Look up studies cited	Look up related studies	Contact the authors of the brief
(1) AI-Generated	-0.133	-0.074	-0.018	0.198	0.122
	(0.132)	(0.155)	(0.152)	(0.151)	(0.184)
RI p-value	[.306]	[.647]	[.898]	[.195]	[.482]
(2) AI-Reported	-0.162	-0.034	0.073	0.071	0.050
	(0.159)	(0.167)	(0.159)	(0.159)	(0.193)
RI p-value	[.25]	[.83]	[.623]	[.648]	[.799]
(3) AI-Generated × AI-Reported	0.037	0.033	-0.109	-0.175	-0.115
	(0.199)	(0.227)	(0.209)	(0.212)	(0.255)
RI p-value (Permuting AI-Generated)	[.869]	[.873]	[.61]	[.433]	[.649]
RI p-value (Permuting AI-Reported)	[.853]	[.878]	[.614]	[.408]	[.638]
Mean of Human-Generated and Human-Reported	2.893	2.667	2.595	2.548	2.226
(1) + (3): Total Effect of AI-Generated	-0.096	-0.041	-0.128	0.022	0.008
(1) + (2) + (3): Total Effect of AI	-0.258*	-0.075	-0.054	0.093	0.058
Observations	366	366	366	366	366

Notes: * $p<0.1$, ** $p<0.05$, *** $p<0.01$. This table reports the effects of the treatments on respondents' beliefs about others' potential engagement with the blog across the five actions separately. Each action is measured using a 5-point Likert scale ranging from 'very unlikely' that others will take the action (0) to 'very likely' (4). An indicator variable is assigned a value of 1 if the response is either 'likely' or 'very likely' to take this action while 'neutral', 'unlikely', and 'very unlikely' are assigned a value of 0. We control for respondent characteristics, wave, and strata (country, seniority, and gender) fixed effects. Standard errors are clustered at the individual level (the unit of randomization.

Table S30. Beliefs in Others' Intended Engagement with the Blog, without Controls - Disaggregated

	(1)	(2)	(3)	(4)	(5)
	Share the blog with others	Re-read the blog	Look up studies cited	Look up related studies	Contact the authors of the brief
(1) AI-Generated	-0.152	-0.072	-0.034	0.122	0.080
	(0.134)	(0.152)	(0.151)	(0.153)	(0.179)
RI p-value	[.274]	[.641]	[.829]	[.433]	[.667]
(2) AI-Reported	-0.173	-0.056	0.049	0.039	-0.050
	(0.147)	(0.158)	(0.150)	(0.153)	(0.181)
RI p-value	[.244]	[.712]	[.737]	[.801]	[.786]
(3) AI-Generated × AI-Reported	0.028	0.016	-0.127	-0.107	-0.039
	(0.199)	(0.223)	(0.209)	(0.212)	(0.246)
RI p-value (Permuting AI-Generated)	[.894]	[.943]	[.535]	[.596]	[.89]
RI p-value (Permuting AI-Reported)	[.895]	[.946]	[.565]	[.612]	[.856]
Mean of Human-Generated and Human-Reported	2.893	2.667	2.595	2.548	2.226
(1) + (3): Total Effect of AI-Generated	-0.124	-0.056	-0.161	0.016	0.041
(1) + (2) + (3): Total Effect of AI	-0.297**	-0.112	-0.111	0.055	-0.008
Observations	366	366	366	366	366

Notes: * $p<0.1$, ** $p<0.05$, *** $p<0.01$. This table reports the effects of the treatments on respondents' beliefs about others' potential engagement with the blog across the five actions separately. Each action is measured using a 5-point Likert scale ranging from 'very unlikely' that others will take the action (0) to 'very likely' (4). An indicator variable is assigned a value of 1 if the response is either 'likely' or 'very likely' while 'neutral', 'unlikely', and 'very unlikely' are assigned a value of 0. We control for wave and strata (country, seniority, and gender) fixed effects. Standard errors are clustered at the individual level (the unit of randomization.

Table S31. Intended Engagement by Senior position, with Controls

	(1)	(2)	(3)	(4)
	Average engagement rating (0-4)	Standardized First Principal Component	Number of 'likely' or 'very likely' statements (0-5)	Log time spent reading the blog (minutes)
(1) AI-Generated	-0.022	-0.023	-0.003	-0.107
	(0.194)	(0.230)	(0.337)	(0.360)
RI p-value	[.899]	[.917]	[.995]	[.76]
(2) AI-Reported	0.199	0.253	0.252	0.190
	(0.187)	(0.220)	(0.359)	(0.404)
RI p-value	[.311]	[.288]	[.5]	[.652]
(3) AI-Generated × AI-Reported	-0.167	-0.214	-0.378	-0.047
	(0.260)	(0.307)	(0.492)	(0.523)
RI p-value (Permuting AI-Generated)	[.515]	[.489]	[.463]	[.934]
RI p-value (Permuting AI-Reported)	[.561]	[.531]	[.476]	[.943]
(4) Senior position	0.186	0.216	0.503	0.537
	(0.363)	(0.411)	(0.727)	(0.504)
(5) AI-Generated × Senior position	-0.261	-0.263	-0.624	0.150
	(0.268)	(0.314)	(0.511)	(0.448)
RI p-value (Permuting AI-Generated)	[.315]	[.403]	[.233]	[.736]
(6) AI-Reported × Senior position	-0.321	-0.361	-0.538	0.007
	(0.265)	(0.311)	(0.534)	(0.499)
RI p-value (Permuting AI-Reported)	[.205]	[.216]	[.309]	[.993]
(7) AI-Generated × AI-Reported × Senior position	0.420	0.462	0.948	-0.085
	(0.373)	(0.438)	(0.725)	(0.646)
RI p-value (Permuting AI-Generated)	[.287]	[.327]	[.209]	[.896]
RI p-value (Permuting AI-Reported)	[.262]	[.289]	[.205]	[.918]
Mean of Human-Generated and Human-Reported	2.729	.085	3.25	.52
(1) + (5): Effect of AI-Generated, Senior position = 1	-0.283	-0.287	-0.626*	0.043
(2) + (6): Effect of AI-Reported, Senior position = 1	-0.121	-0.108	-0.286	0.198
(3) + (7): Effect of AI-Generated × AI-Reported, Senior position = 1	0.252	0.248	0.570	-0.131
(1) + (2) + (3): Total Effect of AI, Senior position = 0	0.010	0.016	-0.128	0.037
(1) + (2) + (3) + (5) + (6) + (7): Total Effect of AI, Senior position = 1	-0.152	-0.147	-0.341	0.110
(5) + (6) + (7): Difference in Total Effect of AI, Senior position = 0 vs. Senior position = 1	-0.162	-0.163	-0.213	0.073
Observations	366	366	366	365

Notes: * p<0.1, ** p<0.05, *** p<0.01. This table reports the heterogeneous effects of the treatments on respondents' reported intended engagement with the blog across five actions by whether the respondent senior position (indicator equal to 1). We include this indicator as well as interact it with indicators for AI-generated blogs, AI-reported blogs, and their interaction (resulting in a triple interaction). Each action is measured using a 5-point Likert scale ranging from 'very unlikely' that the respondent will take this action (0) to 'very likely' (4). The actions are: whether they would share the blog with others, re-read the blog, look up studies cited in the blog, look up related studies, and contact the authors. The outcome in the first column is the simple average across the five actions. The outcome in the second column is the standardized score of the first principal component, calculated based on the same five actions using Polychoric PCA. The outcome in the third column is the total number of the five actions rated as either 'very likely' or 'likely' while 'neutral', 'unlikely', and 'very unlikely' are assigned a value of 0. The outcome in the fourth column is the log number of minutes the respondent spent reading the blog. Note that senior position cannot be permuted since it forms a component of the strata. We control for respondent characteristics, wave, and strata (country, seniority, and gender) fixed effects. Standard errors are clustered at the individual level (the unit of randomization).

Table S32. Intended Engagement by Senior position, without Controls

	(1)	(2)	(3)	(4)
	Average engagement rating (0-4)	Standardized First Principal Component	Number of 'likely' or 'very likely' statements (0-5)	Log time spent reading the blog (minutes)
(1) AI-Generated	-0.061	-0.074	-0.051	-0.158
	(0.178)	(0.208)	(0.320)	(0.358)
RI p-value	[.748]	[.712]	[.885]	[.695]
(2) AI-Reported	0.121	0.163	0.116	0.104
	(0.182)	(0.215)	(0.341)	(0.407)
RI p-value	[.516]	[.462]	[.722]	[.806]
(3) AI-Generated × AI-Reported	-0.107	-0.142	-0.290	-0.030
	(0.247)	(0.290)	(0.468)	(0.501)
RI p-value (Permuting AI-Generated)	[.665]	[.623]	[.586]	[.952]
RI p-value (Permuting AI-Reported)	[.678]	[.66]	[.529]	[.959]
(4) Senior position	0.009	-0.001	-0.082	0.225
	(0.329)	(0.375)	(0.703)	(0.580)
(5) AI-Generated × Senior position	-0.202	-0.196	-0.433	0.066
	(0.253)	(0.296)	(0.509)	(0.438)
RI p-value (Permuting AI-Generated)	[.431]	[.52]	[.37]	[.891]
(6) AI-Reported × Senior position	-0.231	-0.275	-0.254	-0.014
	(0.252)	(0.296)	(0.519)	(0.487)
RI p-value (Permuting AI-Reported)	[.351]	[.336]	[.637]	[.969]
(7) AI-Generated × AI-Reported × Senior position	0.317	0.360	0.625	0.169
	(0.355)	(0.414)	(0.719)	(0.615)
RI p-value (Permuting AI-Generated)	[.366]	[.431]	[.41]	[.785]
RI p-value (Permuting AI-Reported)	[.389]	[.424]	[.403]	[.817]
Mean of Human-Generated and Human-Reported	2.729	.085	3.25	.52
(1) + (5): Effect of AI-Generated, Senior position = 1	-0.263	-0.270	-0.485	-0.092
(2) + (6): Effect of AI-Reported, Senior position = 1	-0.109	-0.111	-0.138	0.090
(3) + (7): Effect of AI-Generated × AI-Reported, Senior position = 1	0.210	0.217	0.336	0.139
(1) + (2) + (3): Total Effect of AI, Senior position = 0	-0.047	-0.053	-0.225	-0.084
(1) + (2) + (3) + (5) + (6) + (7): Total Effect of AI, Senior position = 1	-0.163	-0.164	-0.287	0.136
(5) + (6) + (7): Difference in Total Effect of AI, Senior position = 0 vs. Senior position = 1	-0.116	-0.111	-0.063	0.221
Observations	366	366	366	365

Notes: * p<0.1, ** p<0.05, *** p<0.01. This table reports the heterogeneous effects of the treatments on respondents' reported intended engagement with the blog across five actions by whether the respondent holds a senior position (indicator equal to 1). We include this indicator as well as interact it with indicators for AI-generated blogs, AI-reported blogs, and their interaction (resulting in a triple interaction). Each action is measured using a 5-point Likert scale ranging from 'very unlikely' that the respondent will take this action (0) to 'very likely' (4). The actions are: whether they would share the blog with others, re-read the blog, look up studies cited in the blog, look up related studies, and contact the authors. The outcome in the first column is the simple average across the five actions. The outcome in the second column is the standardized score of the first principal component, calculated based on the same five actions using Polychoric PCA. The outcome in the third column is the total number of the five actions rated as either 'very likely' or 'likely' while 'neutral', 'unlikely', and 'very unlikely' are assigned a value of 0. The outcome in the fourth column is the log number of minutes the respondent spent reading the blog. Note that senior position cannot be permuted since it is a component of the strata. We control for wave and strata (country, seniority, and gender) fixed effects. Standard errors are clustered at the individual level (the unit of randomization).

Table S33. Intended Engagement by Senior Position, with Controls - Disaggregated

	(1)	(2)	(3)	(4)	(5)
	Share the blog with others	Re-read the blog	Look up studies cited	Look up related studies	Contact the authors of the brief
(1) AI-Generated	-0.117	-0.038	0.030	0.062	-0.047
	(0.254)	(0.268)	(0.230)	(0.213)	(0.284)
RI p-value	[.631]	[.899]	[.89]	[.767]	[.874]
(2) AI-Reported	0.030	0.019	0.539**	0.201	0.208
	(0.243)	(0.262)	(0.224)	(0.222)	(0.298)
RI p-value	[.908]	[.943]	[.023]	[.418]	[.478]
(3) AI-Generated × AI-Reported	-0.072	-0.004	-0.520*	-0.077	-0.163
	(0.347)	(0.361)	(0.311)	(0.301)	(0.396)
RI p-value (Permuting AI-Generated)	[.86]	[.989]	[.139]	[.821]	[.687]
RI p-value (Permuting AI-Reported)	[.846]	[.996]	[.116]	[.843]	[.701]
(4) Senior position	0.106	-0.016	0.482	0.234	0.125
	(0.516)	(0.467)	(0.440)	(0.404)	(0.519)
(5) AI-Generated × Senior position	-0.319	-0.381	-0.353	-0.214	-0.037
	(0.370)	(0.371)	(0.334)	(0.299)	(0.381)
RI p-value (Permuting AI-Generated)	[.36]	[.28]	[.261]	[.502]	[.916]
(6) AI-Reported × Senior position	-0.369	-0.017	-0.626*	-0.283	-0.308
	(0.346)	(0.366)	(0.326)	(0.301)	(0.399)
RI p-value (Permuting AI-Reported)	[.24]	[.974]	[.047]	[.372]	[.406]
(7) AI-Generated × AI-Reported × Senior position	0.554	0.297	0.759*	0.301	0.187
	(0.515)	(0.511)	(0.450)	(0.424)	(0.546)
RI p-value (Permuting AI-Generated)	[.276]	[.599]	[.119]	[.516]	[.718]
RI p-value (Permuting AI-Reported)	[.259]	[.563]	[.1]	[.532]	[.718]
Mean of Human-Generated and Human-Reported	2.929	2.833	2.81	2.798	2.274
(1) + (5): Effect of AI-Generated, Senior = 1	-0.435*	-0.419*	-0.324	-0.152	-0.084
(2) + (6): Effect of AI-Reported, Senior = 1	-0.339	0.002	-0.087	-0.082	-0.100
(3) + (7): Effect of AI-Generated × AI-Reported, Senior = 1	0.482	0.293	0.240	0.224	0.024
(1) + (2) + (3): Total Effect of AI, Senior = 0	-0.159	-0.024	0.049	0.186	-0.002
(1) + (2) + (3) + (5) + (6) + (7): Total Effect of AI, Senior = 1	-0.292	-0.125	-0.170	-0.011	-0.160
(5) + (6) + (7): Difference in Total Effect of AI, Senior = 0 vs. Senior = 1	-0.133	-0.101	-0.220	-0.197	-0.157
Observations	366	366	366	366	366

Notes: * p<0.1, ** p<0.05, *** p<0.01. This table reports the heterogeneous effects of the treatments on respondents' reported intended engagement with the blog across the five actions separately by whether the respondent holds a senior position (indicator equal to 1). We include this indicator as well as interact it with indicators for AI-generated blogs, AI-reported blogs, and their interaction (resulting in a triple interaction). Each action is measured using a 5-point Likert scale ranging from 'very unlikely' that the respondent will take this action (0) to 'very likely' (4). An indicator variable is assigned a value of 1 if the response is either 'very likely' or 'likely' while 'neutral', 'unlikely', and 'very unlikely' are assigned a value of 0. Note that senior position cannot be permuted since it is a component of the strata. We control for respondent characteristics, wave, and strata (country, seniority, and gender) fixed effects. Standard errors are clustered at the individual level (the unit of randomization).

Table S34. Intended Engagement by Senior position, without Controls - Disaggregated

	(1)	(2)	(3)	(4)	(5)
	Share the blog with others	Re-read the blog	Look up studies cited	Look up related studies	Contact the authors of the brief
(1) AI-Generated	-0.121	-0.074	-0.053	0.004	-0.061
	(0.235)	(0.250)	(0.208)	(0.199)	(0.274)
RI p-value	[.618]	[.759]	[.819]	[.98]	[.839]
(2) AI-Reported	0.013	0.010	0.418*	0.165	0.000
	(0.235)	(0.254)	(0.222)	(0.219)	(0.289)
RI p-value	[.955]	[.971]	[.057]	[.501]	[1]
(3) AI-Generated × AI-Reported	-0.072	-0.017	-0.383	-0.037	-0.028
	(0.335)	(0.343)	(0.295)	(0.285)	(0.374)
RI p-value (Permuting AI-Generated)	[.841]	[.968]	[.246]	[.896]	[.940]
RI p-value (Permuting AI-Reported)	[.846]	[.958]	[.21]	[.92]	[.942]
(4) Senior position	0.380	-0.432	0.019	0.017	0.059
	(0.426)	(0.389)	(0.417)	(0.384)	(0.507)
(5) AI-Generated × Senior position	-0.354	-0.241	-0.237	-0.144	-0.036
	(0.337)	(0.343)	(0.311)	(0.292)	(0.381)
RI p-value (Permuting AI-Generated)	[.299]	[.481]	[.445]	[.662]	[.929]
(6) AI-Reported × Senior position	-0.261	0.089	-0.528*	-0.265	-0.188
	(0.316)	(0.343)	(0.312)	(0.298)	(0.383)
RI p-value (Permuting AI-Reported)	[.359]	[.8]	[.079]	[.389]	[.595]
(7) AI-Generated × AI-Reported × Senior position	0.464	0.155	0.587	0.262	0.118
	(0.476)	(0.483)	(0.434)	(0.406)	(0.521)
RI p-value (Permuting AI-Generated)	[.34]	[.763]	[.209]	[.589]	[.839]
RI p-value (Permuting AI-Reported)	[.337]	[.757]	[.192]	[.565]	[.804]
Mean of Human-Generated and Human-Reported	2.929	2.833	2.81	2.798	2.274
(1) + (5): Effect of AI-Generated, Senior position = 1	-0.475**	-0.315	-0.290	-0.140	-0.097
(2) + (6): Effect of AI-Reported, Senior position = 1	-0.248	0.099	-0.110	-0.100	-0.188
(3) + (7): Effect of AI-Generated × AI-Reported, Senior position = 1	0.392	0.138	0.204	0.225	0.089
(1) + (2) + (3): Total Effect of AI, Senior position = 0	-0.179	-0.082	-0.017	0.133	-0.089
(1) + (2) + (3) + (5) + (6) + (7): Total Effect of AI, Senior position = 1	-0.330	-0.078	-0.195	-0.015	-0.195
(5) + (6) + (7): Difference in Total Effect of AI, Senior position = 0 vs. Senior position = 1	-0.151	0.004	-0.178	-0.148	-0.106
Observations	366	366	366	366	366

Notes: * p<0.1, ** p<0.05, *** p<0.01. This table reports the heterogeneous effects of the treatments on respondents' planned engagement with the blog across the five actions separately by whether the respondent holds a senior position (indicator equal to 1). We include this indicator as well as interact it with indicators for AI-generated blogs, AI-reported blogs, and their interaction (resulting in a triple interaction). Each action is measured using a 5-point Likert scale ranging from 'very unlikely' that the respondent will take this action (0) to 'very likely' (4). An indicator variable is assigned a value of 1 if the response is either 'very likely' or 'likely' while 'neutral', 'unlikely', and 'very unlikely' are assigned a value of 0. Note that senior position cannot be permuted since it is a component of the strata. We control for wave and strata (country, seniority, and gender) fixed effects. Standard errors are clustered at the individual level (the unit of randomization).

Table S35. Intended Engagement by High Influence in Policy, with Controls

	(1)	(2)	(3)	(4)
	Average engagement rating (0-4)	Standardized First Principal Component	Number of 'likely' or 'very likely' statements (0-5)	Log time spent reading the blog (minutes)
(1) AI-Generated	-0.235	-0.271	-0.455	0.023
	(0.155)	(0.181)	(0.309)	(0.272)
RI p-value	[0.136]	[0.136]	[0.141]	[0.935]
(2) AI-Reported	0.003	0.026	-0.039	0.214
	(0.168)	(0.198)	(0.336)	(0.314)
RI p-value	[0.987]	[0.905]	[0.911]	[0.485]
(3) AI-Generated × AI-Reported	0.155	0.165	0.276	0.075
	(0.224)	(0.262)	(0.462)	(0.413)
RI p-value (Permuting AI-Generated)	[0.512]	[0.545]	[0.538]	[0.865]
RI p-value (Permuting AI-Reported)	[0.54]	[0.57]	[0.574]	[0.856]
(4) High influence in policy	0.062	0.048	0.353	0.226
	(0.189)	(0.220)	(0.393)	(0.355)
RI p-value	[0.784]	[0.852]	[0.41]	[0.494]
(5) AI-Generated × High influence in policy	0.215	0.309	0.346	-0.175
	(0.278)	(0.325)	(0.552)	(0.479)
RI p-value (Permuting AI-Generated)	[0.463]	[0.369]	[0.535]	[0.727]
RI p-value (Permuting High influence in policy)	[0.454]	[0.363]	[0.537]	[0.689]
(6) AI-Reported × High influence in policy	0.091	0.116	0.044	-0.052
	(0.276)	(0.322)	(0.574)	(0.503)
RI p-value (Permuting AI-Reported)	[0.727]	[0.708]	[0.936]	[0.922]
RI p-value (Permuting High influence in policy)	[0.765]	[0.727]	[0.952]	[0.902]
(7) AI-Generated × AI-Reported × High influence in policy	-0.293	-0.389	-0.437	-0.439
	(0.400)	(0.466)	(0.803)	(0.676)
RI p-value (Permuting AI-Generated)	[0.482]	[0.407]	[0.579]	[0.537]
RI p-value (Permuting AI-Reported)	[0.455]	[0.405]	[0.601]	[0.531]
RI p-value (Permuting High influence in policy)	[0.472]	[0.397]	[0.583]	[0.513]
Mean of Human-Generated and Human-Reported	2.729	.085	3.25	.52
(1) + (5): Effect of AI-Generated, Influence = 1	-0.020	0.038	-0.108	-0.151
(2) + (6): Effect of AI-Reported, Influence = 1	0.094	0.142	0.006	0.161
(3) + (7): Effect of AI-Generated × AI-Reported, Influence = 1	-0.139	-0.224	-0.161	-0.364
(1) + (2) + (3): Total Effect of AI, Influence = 0	-0.077	-0.080	-0.217	0.312
(1) + (2) + (3) + (5) + (6) + (7): Total Effect of AI, Influence = 1	-0.064	-0.045	-0.264	-0.354
(5) + (6) + (7): Difference in Total Effect of AI, Influence = 0 vs. Influence = 1	0.013	0.035	-0.047	-0.666
Observations	366	366	366	365

Notes: * p<0.1, ** p<0.05, *** p<0.01. This table reports the heterogeneous effects of the treatments on respondents' reported intended engagement with the blog across five actions by whether the respondent has a high degree of influence on policy (indicator equal to 1). We include this indicator as well as interact it with indicators for AI-generated blogs, AI-reported blogs, and their interaction (resulting in a triple interaction). Each action is measured using a 5-point Likert scale ranging from 'very unlikely' that the respondent will take this action (0) to 'very likely' (4). The actions are: whether they would share the blog with others, re-read the blog, look up studies cited in the blog, look up related studies, and contact the authors. The outcome in the first column is the simple average across the five actions. The outcome in the second column is the standardized score of the first principal component, calculated based on the same five actions using Polychoric PCA. The outcome in the third column is the total number of the five actions rated as either 'very likely' or 'likely' while 'neutral', 'unlikely', and 'very unlikely' are assigned a value of 0. The outcome in the fourth column is the log number of minutes the respondent spent reading the blog. We control for respondent characteristics, wave, and strata (country, seniority, and gender) fixed effects. Standard errors are clustered at the individual level (the unit of randomization).

Table S36. Intended Engagement by High Influence in Policy, without Controls

	(1)	(2)	(3)	(4)
	Average engagement rating (0-4)	Standardized First Principal Component	Number of 'likely' or 'very likely' statements (0-5)	Log time spent reading the blog (minutes)
(1) AI-Generated	-0.264*	-0.309*	-0.416	-0.050
	(0.150)	(0.175)	(0.301)	(0.271)
RI p-value	[.094]	[.083]	[.164]	[.839]
(2) AI-Reported	-0.011	0.005	-0.007	0.063
	(0.157)	(0.186)	(0.312)	(0.304)
RI p-value	[.951]	[.986]	[.986]	[.837]
(3) AI-Generated × AI-Reported	0.190	0.214	0.240	0.152
	(0.214)	(0.250)	(0.434)	(0.395)
RI p-value (Permuting AI-Generated)	[.407]	[.401]	[.593]	[.693]
RI p-value (Permuting AI-Reported)	[.406]	[.427]	[.601]	[.73]
(4) High influence in policy	0.153	0.152	0.575	0.142
	(0.186)	(0.216)	(0.394)	(0.366)
RI p-value	[.482]	[.523]	[.124]	[.696]
(5) AI-Generated × High influence in policy	0.271	0.371	0.381	-0.219
	(0.274)	(0.319)	(0.556)	(0.476)
RI p-value (Permuting AI-Generated)	[.309]	[.262]	[.516]	[.622]
RI p-value (Permuting High influence in policy)	[.341]	[.27]	[.473]	[.625]
(6) AI-Reported × High influence in policy	0.044	0.054	0.013	0.099
	(0.262)	(0.308)	(0.557)	(0.504)
RI p-value (Permuting AI-Reported)	[.88]	[.857]	[.976]	[.855]
RI p-value (Permuting High influence in policy)	[.861]	[.876]	[.978]	[.845]
(7) AI-Generated × AI-Reported × High influence in policy	-0.359	-0.459	-0.534	-0.240
	(0.380)	(0.442)	(0.779)	(0.670)
RI p-value (Permuting AI-Generated)	[.329]	[.306]	[.482]	[.726]
RI p-value (Permuting AI-Reported)	[.358]	[.331]	[.529]	[.726]
RI p-value (Permuting High influence in policy)	[.342]	[.285]	[.475]	[.695]
Mean of Human-Generated and Human-Reported	2.729	.085	3.25	.52
(1) + (5): Effect of AI-Generated, Influence = 1	0.008	0.062	-0.035	-0.269
(2) + (6): Effect of AI-Reported, Influence = 1	0.034	0.058	0.006	0.162
(3) + (7): Effect of AI-Generated × AI-Reported, Influence = 1	-0.170	-0.245	-0.294	-0.087
(1) + (2) + (3): Total Effect of AI, Influence = 0	-0.085	-0.091	-0.183	0.166
(1) + (2) + (3) + (5) + (6) + (7): Total Effect of AI, Influence = 1	-0.128	-0.124	-0.322	-0.194
(5) + (6) + (7): Difference in Total Effect of AI, Influence = 0 vs. Influence = 1	-0.044	-0.034	-0.139	-0.359
Observations	366	366	366	365

Notes: * p<0.1, ** p<0.05, *** p<0.01. This table reports the heterogeneous effects of the treatments on respondents' reported intended engagement with the blog across five actions by whether the respondent has a high degree of influence on policy (indicator equal to 1). We include this indicator as well as interact it with indicators for AI-generated blogs, AI-reported blogs, and their interaction (resulting in a triple interaction). Each action is measured using a 5-point Likert scale ranging from 'very unlikely' that the respondent will take this action (0) to 'very likely' (4). The actions are: whether they would share the blog with others, re-read the blog, look up studies cited in the blog, look up related studies, and contact the authors. The outcome in the first column is the simple average across the five actions. The outcome in the second column is the standardized score of the first principal component, calculated based on the same five actions using Polychoric PCA. The outcome in the third column is the total number of the five actions rated as either 'very likely' or 'likely' while 'neutral', 'unlikely', and 'very unlikely' are assigned a value of 0. The outcome in the fourth column is the log number of minutes the respondent spent reading the blog. We control for wave and strata (country, seniority, and gender) fixed effects. Standard errors are clustered at the individual level (the unit of randomization).

Table S37. Intended Engagement by High Influence in Policy, with Controls - Disaggregated

	(1)	(2)	(3)	(4)	(5)
	Share the blog with others	Re-read the blog	Look up studies cited	Look up related studies	Contact the authors of the brief
(1) AI-Generated	-0.213	-0.293	-0.233	-0.173	-0.263
	(0.218)	(0.217)	(0.185)	(0.186)	(0.230)
RI p-value	[.323]	[.199]	[.193]	[.373]	[.253]
(2) AI-Reported	-0.127	-0.092	0.190	0.098	-0.052
	(0.220)	(0.221)	(0.213)	(0.197)	(0.248)
RI p-value	[.568]	[.698]	[.38]	[.635]	[.844]
(3) AI-Generated × AI-Reported	0.097	0.384	-0.063	0.169	0.185
	(0.303)	(0.304)	(0.277)	(0.263)	(0.328)
RI p-value (Permuting AI-Generated)	[.748]	[.213]	[.825]	[.584]	[.551]
RI p-value (Permuting AI-Reported)	[.768]	[.231]	[.835]	[.559]	[.576]
(4) High influence in policy	0.269	-0.001	0.043	0.090	-0.091
	(0.261)	(0.253)	(0.236)	(0.226)	(0.285)
RI p-value	[.338]	[.996]	[.872]	[.763]	[.726]
(5) AI-Generated × High influence in policy	-0.175	0.126	0.238	0.340	0.548
	(0.380)	(0.366)	(0.356)	(0.304)	(0.418)
RI p-value (Permuting AI-Generated)	[.67]	[.757]	[.5]	[.287]	[.204]
RI p-value (Permuting High influence in policy)	[.645]	[.757]	[.523]	[.321]	[.156]
(6) AI-Reported × High influence in policy	-0.072	0.302	0.089	-0.141	0.276
	(0.365)	(0.376)	(0.345)	(0.318)	(0.419)
RI p-value (Permuting AI-Reported)	[.821]	[.462]	[.772]	[.66]	[.461]
RI p-value (Permuting High influence in policy)	[.849]	[.449]	[.804]	[.71]	[.48]
(7) AI-Generated × AI-Reported × High influence in policy	0.319	-0.656	-0.185	-0.233	-0.712
	(0.533)	(0.540)	(0.521)	(0.426)	(0.572)
RI p-value (Permuting AI-Generated)	[.542]	[.209]	[.703]	[.608]	[.205]
RI p-value (Permuting AI-Reported)	[.537]	[.218]	[.698]	[.595]	[.184]
RI p-value (Permuting High influence in policy)	[.541]	[.24]	[.698]	[.585]	[.2]
Mean of Human-Generated and Human-Reported	2.929	2.833	2.81	2.798	2.274
(1) + (5): Effect of AI-Generated, Influence = 1	-0.388	-0.167	0.005	0.167	0.285
(2) + (6): Effect of AI-Reported, Influence = 1	-0.199	0.210	0.279	-0.043	0.224
(3) + (7): Effect of AI-Generated × AI-Reported, Influence = 1	0.416	-0.273	-0.248	-0.063	-0.527
(1) + (2) + (3): Total Effect of AI, Influence = 0	-0.243	-0.001	-0.106	0.094	-0.129
(1) + (2) + (3) + (5) + (6) + (7): Total Effect of AI, Influence = 1	-0.171	-0.229	0.036	0.060	-0.018
(5) + (6) + (7): Difference in Total Effect of AI, Influence = 0 vs. Influence = 1	0.072	-0.228	0.142	-0.034	0.112
Observations	366	366	366	366	366

Notes: * p<0.1, ** p<0.05, *** p<0.01. This table reports the heterogeneous effects of the treatments on respondents' reported intended engagement with the blog across the five actions separately by whether the respondent has a high degree of influence in policy (indicator equal to 1). We include this indicator as well as interact it with indicators for AI-generated blogs, AI-reported blogs, and their interaction has a high degree of influence on policy (resulting in a triple interaction). Each action is measured using a 5-point Likert scale ranging from 'very unlikely' that the respondent will take this action (0) to 'very likely' (4). An indicator variable is assigned a value of 1 if the response is either 'very likely' or 'likely' while 'neutral', 'unlikely', and 'very unlikely' are assigned a value of 0. We control for respondent characteristics, wave, and strata (country, seniority, and gender) fixed effects. Standard errors are clustered at the individual level (the unit of randomization).

Table S38. Intended Engagement by High Influence in Policy, without Controls - Disaggregated

	(1)	(2)	(3)	(4)	(5)
	Share the blog with others	Re-read the blog	Look up studies cited	Look up related studies	Contact the authors of the brief
(1) AI-Generated	-0.248	-0.268	-0.283	-0.193	-0.328
	(0.205)	(0.211)	(0.183)	(0.184)	(0.231)
RI p-value	[.235]	[.213]	[.1]	[.304]	[.167]
(2) AI-Reported	-0.095	0.008	0.128	0.092	-0.187
	(0.206)	(0.213)	(0.205)	(0.185)	(0.243)
RI p-value	[.659]	[.969]	[.523]	[.632]	[.455]
(3) AI-Generated × AI-Reported	0.092	0.289	0.031	0.198	0.339
	(0.293)	(0.293)	(0.268)	(0.255)	(0.327)
RI p-value (Permuting AI-Generated)	[.758]	[.341]	[.907]	[.49]	[.288]
RI p-value (Permuting AI-Reported)	[.777]	[.352]	[.898]	[.461]	[.304]
(4) High influence in policy	0.320	0.135	0.114	0.220	-0.026
	(0.235)	(0.240)	(0.230)	(0.228)	(0.289)
RI p-value	[.218]	[.608]	[.671]	[.411]	[.914]
(5) AI-Generated × High influence in policy	-0.156	0.176	0.306	0.341	0.690*
	(0.373)	(0.358)	(0.353)	(0.310)	(0.413)
RI p-value (Permuting AI-Generated)	[.677]	[.621]	[.378]	[.294]	[.119]
RI p-value (Permuting High influence in policy)	[.68]	[.644]	[.399]	[.298]	[.088]
(6) AI-Reported × High influence in policy	-0.050	0.149	0.052	-0.182	0.253
	(0.321)	(0.363)	(0.325)	(0.319)	(0.397)
RI p-value (Permuting AI-Reported)	[.887]	[.696]	[.861]	[.566]	[.524]
RI p-value (Permuting High influence in policy)	[.905]	[.674]	[.88]	[.621]	[.492]
(7) AI-Generated × AI-Reported × High influence in policy	0.220	-0.619	-0.291	-0.257	-0.850
	(0.515)	(0.519)	(0.493)	(0.428)	(0.551)
RI p-value (Permuting AI-Generated)	[.664]	[.225]	[.551]	[.572]	[.12]
RI p-value (Permuting AI-Reported)	[.648]	[.251]	[.545]	[.575]	[.116]
RI p-value (Permuting High influence in policy)	[.656]	[.238]	[.525]	[.538]	[.117]
Mean of Human-Generated and Human-Reported	2.929	2.833	2.81	2.798	2.274
(1) + (5): Effect of AI-Generated, Influence = 1	-0.404	-0.092	0.023	0.149	0.363
(2) + (6): Effect of AI-Reported, Influence = 1	-0.145	0.157	0.180	-0.090	0.066
(3) + (7): Effect of AI-Generated × AI-Reported, Influence = 1	0.312	-0.330	-0.260	-0.058	-0.511
(1) + (2) + (3): Total Effect of AI, Influence = 0	-0.251	0.029	-0.124	0.097	-0.175
(1) + (2) + (3) + (5) + (6) + (7): Total Effect of AI, Influence = 1	-0.237	-0.266	-0.058	0.000	-0.082
(5) + (6) + (7): Difference in Total Effect of AI, Influence = 0 vs. Influence = 1	0.014	-0.295	0.067	-0.097	0.093
Observations	366	366	366	366	366

Notes: * p<0.1, ** p<0.05, *** p<0.01. This table reports the heterogeneous effects of the treatments on respondents' planned engagement with the blog across the five actions separately by whether the respondent has a high degree of influence on policy (indicator equal to 1). We include this indicator as well as interact it with indicators for AI-generated blogs, AI-reported blogs, and their interaction (resulting in a triple interaction). Each action is measured using a 5-point Likert scale ranging from 'very unlikely' that the respondent will take this action (0) to 'very likely' (4). An indicator variable is assigned a value of 1 if the response is either 'very likely' or 'likely' while 'neutral', 'unlikely', and 'very unlikely' are assigned a value of 0. We control for wave and strata (country, seniority, and gender) fixed effects. Standard errors are clustered at the individual level (the unit of randomization).

Table S39. Intended Engagement by Overall Quality of Blog Rated 'High' or 'Very High', with Controls

	(1)	(2)	(3)	(4)
	Average engagement rating (0-4)	Standardized First Principal Component	Number of 'likely' or 'very likely' statements (0-5)	Log time spent reading the blog (minutes)
(1) AI-Generated	-0.285	-0.290	-0.260	-0.491
	(0.227)	(0.252)	(0.448)	(0.351)
RI p-value	[.183]	[.209]	[.518]	[.137]
(2) AI-Reported	-0.144	-0.119	-0.144	0.195
	(0.230)	(0.261)	(0.449)	(0.401)
RI p-value	[.501]	[.606]	[.705]	[.642]
(3) AI-Generated × AI-Reported	0.139	0.107	0.068	0.070
	(0.299)	(0.333)	(0.600)	(0.523)
RI p-value (Permuting AI-Generated)	[.643]	[.742]	[.906]	[.892]
RI p-value (Permuting AI-Reported)	[.625]	[.738]	[.902]	[.888]
(4) Overall quality of brief rated 'high' or 'very high'	0.424*	0.503*	1.172***	-0.383
	(0.222)	(0.256)	(0.440)	(0.350)
RI p-value	[.031]	[.024]	[0]	[.217]
(5) AI-Generated × Overall quality of brief rated 'high' or 'very high'	0.355	0.382	0.210	0.747*
	(0.274)	(0.316)	(0.548)	(0.449)
RI p-value (Permuting AI-Generated)	[.175]	[.215]	[.687]	[.096]
RI p-value (Permuting Overall quality of brief rated 'high' or 'very high')	[.252]	[.269]	[.661]	[.077]
(6) AI-Reported × Overall quality of brief rated 'high' or 'very high'	0.399	0.428	0.453	-0.081
	(0.269)	(0.314)	(0.542)	(0.516)
RI p-value (Permuting AI-Reported)	[.127]	[.151]	[.337]	[.857]
RI p-value (Permuting Overall quality of brief rated 'high' or 'very high')	[.119]	[.169]	[.368]	[.846]
(7) AI-Generated × AI-Reported × Overall quality of brief rated 'high' or 'very high'	-0.386	-0.409	-0.424	-0.218
	(0.366)	(0.422)	(0.744)	(0.682)
RI p-value (Permuting AI-Generated)	[.311]	[.346]	[.572]	[.752]
RI p-value (Permuting AI-Reported)	[.262]	[.31]	[.544]	[.725]
RI p-value (Permuting Overall quality of brief rated 'high' or 'very high')	[.317]	[.357]	[.576]	[.765]
Mean of Human-Generated and Human-Reported	2.729	.085	3.25	.52
(1) + (5): Effect of AI-Generated, High Quality = 1	0.070	0.092	-0.050	0.257
(2) + (6): Effect of AI-Reported, High Quality = 1	0.255*	0.309*	0.309	0.114
(3) + (7): Effect of AI-Generated × AI-Reported, High Quality = 1	-0.247	-0.302	-0.356	-0.148
(1) + (2) + (3): Total Effect of AI, High Quality = 0	-0.290	-0.301	-0.337	-0.226
(1) + (2) + (3) + (5) + (6) + (7): Total Effect of AI, High Quality = 1	0.078	0.100	-0.098	0.223
(5) + (6) + (7): Difference in Total Effect of AI, High Quality = 0 vs. High Quality = 1	0.368	0.401	0.239	0.448
Observations	366	366	366	365

Notes: * p<0.1, ** p<0.05, *** p<0.01. This table reports the heterogeneous effects of the treatments on respondents' reported intended engagement with the blog across five actions by whether the respondent rated the quality of the blog as being 'high' or 'very high' (indicator equal to 1). We include this indicator as well as interact it with indicators for AI-generated blogs, AI-reported blogs, and their interaction (resulting in a triple interaction). Each action is measured using a 5-point Likert scale ranging from 'very unlikely' that the respondent will take this action (0) to 'very likely' (4). The actions are: whether they would share the blog with others, re-read the blog, look up studies cited in the blog, look up related studies, and contact the authors. The outcome in the first column is the simple average across the five actions. The outcome in the second column is the standardized score of the first principal component, calculated based on the same five actions using Polychoric PCA. The outcome in the third column is the total number of the five actions rated as either 'very likely' or 'likely' while 'neutral', 'unlikely', and 'very unlikely' are assigned a value of 0. The outcome in the fourth column is the log number of minutes the respondent spent reading the blog. We control for respondent characteristics, wave, and strata (country, seniority, and gender) fixed effects. Standard errors are clustered at the individual level (the unit of randomization).

Table S40. Intended Engagement by Overall Quality of Blog Rated 'High' or 'Very High', without Controls

	(1)	(2)	(3)	(4)
	Average engagement rating (0-4)	Standardized First Principal Component	Number of 'likely' or 'very likely' statements (0-5)	Log time spent reading the blog (minutes)
(1) AI-Generated	-0.256	-0.260	-0.238	-0.667**
	(0.221)	(0.245)	(0.430)	(0.338)
RI p-value	[.219]	[.244]	[.565]	[.039]
(2) AI-Reported	-0.147	-0.138	-0.132	0.015
	(0.221)	(0.251)	(0.436)	(0.377)
RI p-value	[.465]	[.566]	[.771]	[.969]
(3) AI-Generated × AI-Reported	0.103	0.077	-0.075	0.275
	(0.294)	(0.326)	(0.585)	(0.483)
RI p-value (Permuting AI-Generated)	[.701]	[.810]	[.906]	[.554]
RI p-value (Permuting AI-Reported)	[.711]	[.82]	[.908]	[.557]
(4) Overall quality of brief rated 'high' or 'very high'	0.469**	0.551**	1.145***	-0.525
	(0.216)	(0.248)	(0.427)	(0.360)
RI p-value	[.027]	[.017]	[.002]	[.113]
(5) AI-Generated × Overall quality of brief rated 'high' or 'very high'	0.318	0.334	0.287	0.863*
	(0.266)	(0.306)	(0.532)	(0.451)
RI p-value (Permuting AI-Generated)	[.229]	[.27]	[.585]	[.052]
RI p-value (Permuting Overall quality of brief rated 'high' or 'very high')	[.347]	[.395]	[.623]	[.026]
(6) AI-Reported × Overall quality of brief rated 'high' or 'very high'	0.341	0.373	0.410	0.050
	(0.261)	(0.303)	(0.533)	(0.501)
RI p-value (Permuting AI-Reported)	[.176]	[.197]	[.426]	[.917]
RI p-value (Permuting Overall quality of brief rated 'high' or 'very high')	[.187]	[.226]	[.384]	[.916]
(7) AI-Generated × AI-Reported × Overall quality of brief rated 'high' or 'very high'	-0.312	-0.321	-0.284	-0.304
	(0.362)	(0.416)	(0.738)	(0.661)
RI p-value (Permuting AI-Generated)	[.429]	[.456]	[.687]	[.658]
RI p-value (Permuting AI-Reported)	[.359]	[.419]	[.695]	[.63]
RI p-value (Permuting Overall quality of brief rated 'high' or 'very high')	[.421]	[.478]	[.7]	[.617]
Mean of Human-Generated and Human-Reported	2.729	.085	3.25	.52
(1) + (5): Effect of AI-Generated, High Quality = 1	0.063	0.074	0.048	0.196
(2) + (6): Effect of AI-Reported, High Quality = 1	0.194	0.235	0.278	0.065
(3) + (7): Effect of AI-Generated × AI-Reported, High Quality = 1	-0.209	-0.245	-0.360	-0.029
(1) + (2) + (3): Total Effect of AI, High Quality = 0	-0.300	-0.321	-0.445	-0.376
(1) + (2) + (3) + (5) + (6) + (7): Total Effect of AI, High Quality = 1	0.048	0.064	-0.034	0.232
(5) + (6) + (7): Difference in Total Effect of AI, High Quality = 0 vs. High Quality = 1	0.348	0.385	0.412	0.608
Observations	366	366	366	365

Notes: * p<0.1, ** p<0.05, *** p<0.01. This table reports the heterogeneous effects of the treatments on respondents' reported intended engagement with the blog across five actions by whether the respondent rated the quality of the blog as being 'high' or 'very high' (indicator equal to 1). We include this indicator as well as interact it with indicators for AI-generated blogs, AI-reported blogs, and their interaction (resulting in a triple interaction). Each action is measured using a 5-point Likert scale ranging from 'very unlikely' that the respondent will take this action (0) to 'very likely' (4). The actions are: whether they would share the blog with others, re-read the blog, look up studies cited in the blog, look up related studies, and contact the authors. The outcome in the first column is the simple average across the five actions. The outcome in the second column is the standardized score of the first principal component, calculated based on the same five actions using Polychoric PCA. The outcome in the third column is the total number of the five actions rated as either 'very likely' or 'likely' while 'neutral', 'unlikely', and 'very unlikely' are assigned a value of 0. The outcome in the fourth column is the log number of minutes the respondent spent reading the blog. We control for wave and strata (country, seniority, and gender) fixed effects. Standard errors are clustered at the individual level (the unit of randomization).

Table S41. Intended Engagement by Overall Quality of Blog Rated 'High' or 'Very High', with Controls - Disaggregated

	(1) Share the blog with others	(2) Re-read the blog	(3) Look up studies cited	(4) Look up related studies	(5) Contact the authors of the brief
(1) AI-Generated	-0.539*	-0.606*	-0.208	0.009	-0.080
	(0.297)	(0.311)	(0.272)	(0.264)	(0.321)
RI p-value	[.077]	[.068]	[.419]	[.963]	[.79]
(2) AI-Reported	-0.413	-0.190	0.242	0.001	-0.358
	(0.298)	(0.318)	(0.307)	(0.292)	(0.309)
RI p-value	[.164]	[.543]	[.426]	[.996]	[.221]
(3) AI-Generated × AI-Reported	0.357	0.093	-0.151	0.034	0.362
	(0.394)	(0.427)	(0.392)	(0.359)	(0.414)
RI p-value (Permuting AI-Generated)	[.381]	[.838]	[.7]	[.921]	[.376]
RI p-value (Permuting AI-Reported)	[.386]	[.814]	[.689]	[.929]	[.388]
(4) Overall quality of brief rated 'high' or 'very high'	0.606**	0.225	0.565**	0.352	0.371
	(0.292)	(0.295)	(0.264)	(0.264)	(0.319)
RI p-value	[.025]	[.394]	[.01]	[.084]	[.27]
(5) AI-Generated × Overall quality of brief rated 'high' or 'very high'	0.642*	0.733**	0.272	-0.005	0.130
	(0.356)	(0.371)	(0.330)	(0.328)	(0.401)
RI p-value (Permuting AI-Generated)	[.064]	[.061]	[.388]	[.985]	[.734]
RI p-value (Permuting Overall quality of brief rated high or very high)	[.106]	[.056]	[.39]	[.977]	[.782]
(6) AI-Reported × Overall quality of brief rated 'high' or 'very high'	0.565	0.392	0.080	0.169	0.788**
	(0.347)	(0.385)	(0.358)	(0.347)	(0.385)
RI p-value (Permuting AI-Reported)	[.088]	[.303]	[.822]	[.602]	[.027]
RI p-value (Permuting Overall quality of brief rated 'high' or 'very high')	[.088]	[.22]	[.810]	[.551]	[.042]
(7) AI-Generated × AI-Reported × Overall quality of brief rated 'high' or 'very high'	-0.591	-0.118	-0.216	-0.076	-0.927*
	(0.489)	(0.523)	(0.479)	(0.453)	(0.539)
RI p-value (Permuting AI-Generated)	[.23]	[.831]	[.647]	[.851]	[.076]
RI p-value (Permuting AI-Reported)	[.202]	[.817]	[.638]	[.859]	[.074]
RI p-value (Permuting Overall quality of brief rated 'high' or 'very high')	[.243]	[.82]	[.65]	[.846]	[.081]
Mean of Human-Generated and Human-Reported	2.929	2.833	2.81	2.798	2.274
(1) + (5): Effect of AI-Generated, High Quality = 1	0.104	0.127	0.064	0.004	0.050
(2) + (6): Effect of AI-Reported, High Quality = 1	0.152	0.201	0.321*	0.170	0.430*
(3) + (7): Effect of AI-Generated × AI-Reported, High Quality = 1	-0.233	-0.025	-0.367	-0.042	-0.566
(1) + (2) + (3): Total Effect of AI, High Quality = 0	-0.594*	-0.704**	-0.118	0.045	-0.077
(1) + (2) + (3) + (5) + (6) + (7): Total Effect of AI, High Quality = 1	0.022	0.303*	0.018	0.133	-0.085
(5) + (6) + (7): Difference in Total Effect of AI, High Quality = 0 vs. High Quality = 1	0.616	1.007***	0.136	0.088	-0.008
Observations	366	366	366	366	366

Notes: * p<0.1, ** p<0.05, *** p<0.01. This table reports the heterogeneous effects of the treatments on respondents' reported intended engagement with the blog across the five actions separately by whether the respondent rated the quality of the blog as being 'high' or 'very high' (indicator equal to 1). We include this indicator as well as interact it with indicators for AI-generated blogs, AI-reported blogs, and their interaction (resulting in a triple interaction). Each action is measured using a 5-point Likert scale ranging from 'very unlikely' that the respondent will take this action (0) to 'very likely' (4). An indicator variable is assigned a value of 1 if the response is either 'very likely' or 'likely' while 'neutral', 'unlikely', and 'very unlikely' are assigned a value of 0. We control for respondent characteristics, wave, and strata (country, seniority, and gender) fixed effects. Standard errors are clustered at the individual level (the unit of randomization).

Table S42. Intended Engagement by Overall Quality of Blog Rated 'High' or 'Very High', without Controls - Disaggregated

	(1)	(2)	(3)	(4)	(5)
	Share the blog with others	Re-read the blog	Look up studies cited	Look up related studies	Contact the authors of the brief
(1) AI-Generated	-0.497*	-0.486	-0.179	-0.039	-0.077
	(0.284)	(0.314)	(0.279)	(0.257)	(0.316)
RI p-value	[.082]	[.139]	[.514]	[.872]	[.810]
(2) AI-Reported	-0.306	-0.096	0.201	-0.079	-0.456
	(0.287)	(0.326)	(0.306)	(0.277)	(0.289)
RI p-value	[.299]	[.748]	[.483]	[.762]	[.102]
(3) AI-Generated × AI-Reported	0.279	-0.023	-0.194	0.056	0.398
	(0.388)	(0.437)	(0.388)	(0.352)	(0.405)
RI p-value (Permuting AI-Generated)	[.487]	[.963]	[.625]	[.878]	[.327]
RI p-value (Permuting AI-Reported)	[.478]	[.962]	[.62]	[.88]	[.325]
(4) Overall quality of brief rated 'high' or 'very high'	0.681**	0.332	0.596**	0.305	0.433
	(0.274)	(0.290)	(0.267)	(0.258)	(0.311)
RI p-value	[.006]	[.201]	[.007]	[.176]	[.217]
(5) AI-Generated × Overall quality of brief rated 'high' or 'very high'	0.577*	0.648*	0.195	0.040	0.132
	(0.345)	(0.368)	(0.333)	(0.322)	(0.401)
RI p-value (Permuting AI-Generated)	[.075]	[.095]	[.525]	[.899]	[.762]
RI p-value (Permuting Overall quality of brief rated 'high' or 'very high')	[.114]	[.118]	[.554]	[.906]	[.831]
(6) AI-Reported × Overall quality of brief rated 'high' or 'very high'	0.441	0.330	0.009	0.237	0.690*
	(0.336)	(0.383)	(0.357)	(0.335)	(0.374)
RI p-value (Permuting AI-Reported)	[.171]	[.379]	[.981]	[.442]	[.046]
RI p-value (Permuting Overall quality of brief rated 'high' or 'very high')	[.154]	[.295]	[.979]	[.423]	[.072]
(7) AI-Generated × AI-Reported × Overall quality of brief rated 'high' or 'very high'	-0.536	-0.104	-0.046	-0.061	-0.812
	(0.488)	(0.525)	(0.473)	(0.444)	(0.543)
RI p-value (Permuting AI-Generated)	[.272]	[.851]	[.937]	[.884]	[.125]
RI p-value (Permuting AI-Reported)	[.229]	[.831]	[.91]	[.886]	[.122]
RI p-value (Permuting Overall quality of brief rated 'high' or 'very high')	[.283]	[.842]	[.917]	[.877]	[.146]
Mean of Human-Generated and Human-Reported	2.929	2.833	2.81	2.798	2.274
(1) + (5): Effect of AI-Generated, High Quality = 1	0.080	0.161	0.016	0.001	0.055
(2) + (6): Effect of AI-Reported, High Quality = 1	0.136	0.233	0.210	0.158	0.234
(3) + (7): Effect of AI-Generated × AI-Reported, High Quality = 1	-0.257	-0.127	-0.240	-0.005	-0.414
(1) + (2) + (3): Total Effect of AI, High Quality = 0	-0.523	-0.606*	-0.172	-0.062	-0.135
(1) + (2) + (3) + (5) + (6) + (7): Total Effect of AI, High Quality = 1	-0.042	0.268	-0.014	0.154	-0.125
(5) + (6) + (7): Difference in Total Effect of AI, High Quality = 0 vs. High Quality = 1	0.482	0.874**	0.159	0.216	0.011
Observations	366	366	366	366	366

Notes: * p<0.1, ** p<0.05, *** p<0.01. This table reports the heterogeneous effects of the treatments on respondents' planned engagement with the blog across the five actions separately by whether the respondent rated the quality of the blog as being 'high' or 'very high' (indicator equal to 1). We include this indicator as well as interact it with indicators for AI-generated blogs, AI-reported blogs, and their interaction (resulting in a triple interaction). Each action is measured using a 5-point Likert scale ranging from 'very unlikely' that the respondent will take this action (0) to 'very likely' (4). An indicator variable is assigned a value of 1 if the response is either 'very likely' or 'likely' while 'neutral', 'unlikely', and 'very unlikely' are assigned a value of 0. We control for wave and strata (country, seniority, and gender) fixed effects. Standard errors are clustered at the individual level (the unit of randomization).

Table S43. Beliefs about Others' Engagement by Senior Position, with Controls

	(1)	(2)	(3)	(4)
	Others' Average Engagement Rating (0-4)	Standardized First Principal Component	Number of 'likely' or 'very likely' statements (0-5)	Others' overall rating 'high' or 'very high'
(1) AI-Generated	0.096	0.131	0.146	-0.020
	(0.182)	(0.219)	(0.408)	(0.112)
RI p-value	[.576]	[.53]	[.713]	[.86]
(2) AI-Reported	-0.118	-0.149	-0.308	-0.028
	(0.200)	(0.239)	(0.426)	(0.103)
RI p-value	[.542]	[.522]	[.459]	[.767]
(3) AI-Generated × AI-Reported	0.091	0.088	0.277	0.059
	(0.258)	(0.310)	(0.578)	(0.155)
RI p-value (Permuting AI-Generated)	[.752]	[.808]	[.64]	[.714]
RI p-value (Permuting AI-Reported)	[.727]	[.791]	[.608]	[.695]
(4) Senior position	-0.137	-0.171	-0.415	-0.032
	(0.353)	(0.419)	(0.774)	(0.209)
(5) AI-Generated × Senior position	-0.141	-0.179	-0.304	-0.164
	(0.255)	(0.305)	(0.564)	(0.166)
RI p-value (Permuting AI-Generated)	[.574]	[.55]	[.581]	[.297]
(6) AI-Reported × Senior position	0.233	0.291	0.727	-0.020
	(0.273)	(0.325)	(0.599)	(0.153)
RI p-value (Permuting AI-Reported)	[.392]	[.365]	[.223]	[.887]
(7) AI-Generated × AI-Reported × Senior position	-0.295	-0.328	-0.801	-0.015
	(0.370)	(0.443)	(0.810)	(0.230)
RI p-value (Permuting AI-Generated)	[.399]	[.441]	[.338]	[.944]
RI p-value (Permuting AI-Reported)	[.375]	[.399]	[.301]	[.934]
Mean of Human-Generated and Human-Reported	2.586	.064	2.917	.679
(1) + (5): Effect of AI-Generated, Senior position = 1	-0.044	-0.048	-0.158	-0.184
(2) + (6): Effect of AI-Reported, Senior position = 1	0.115	0.142	0.419	-0.048
(3) + (7): Effect of AI-Generated × AI-Reported, Senior position = 1	-0.204	-0.240	-0.524	0.045
(1) + (2) + (3): Total Effect of AI, Senior position = 0	0.069	0.070	0.114	0.012
(1) + (2) + (3) + (5) + (6) + (7): Total Effect of AI, Senior position = 1	-0.134	-0.145	-0.264	-0.187*
(5) + (6) + (7): Difference in Total Effect of AI, Senior position = 0 vs. Senior position = 1	-0.203	-0.215	-0.378	-0.199
Observations	366	366	366	366

Notes: * p<0.1, ** p<0.05, *** p<0.01. This table reports the heterogeneous effects of the treatments on respondents' beliefs about others' potential engagement with the blog across the five actions by whether the respondent holds a senior position (indicator equal to 1). We include this indicator as well as interact it with indicators for AI-generated blogs, AI-reported blogs, and their interaction (resulting in a triple interaction). Each action is measured using a 5-point Likert scale ranging from 'very unlikely' that others will take this action (0) to 'very likely' (4). The actions were: whether they would share the blog with others, re-read the blog, look up studies cited in the blog, look up related studies, and contact the authors. The outcome in the first column is the simple average across the five actions. The outcome in the second column is the standardized score of the first principal component, calculated based on the same five actions using Polychoric PCA. The outcome in the third column is the total number of the five actions rated as either 'very likely' or 'likely' while 'neutral', 'unlikely', and 'very unlikely' are assigned a value of 0. The outcome in the fourth column is an indicator equal to one if the respondent believes others' potential engagement is rated as either 'high' or 'very high', while 'neutral', 'low', and 'very low' are assigned a value of 0. Note that senior position cannot be permuted since it is a component of the strata. We control for respondent characteristics, wave, and strata (country, seniority, and gender) fixed effects. Standard errors are clustered at the individual level (the unit of randomization).

Table S44. Beliefs about Others' Engagement by Senior Position, without Controls

	(1)	(2)	(3)	(4)
	Others' Average Engagement Rating (0-4)	Standardized First Principal Component	Number of 'likely' or 'very likely' statements (0-5)	Others' overall rating 'high' or 'very high'
(1) AI-Generated	0.019	0.031	0.102	-0.043
	(0.175)	(0.213)	(0.386)	(0.107)
RI p-value	[.911]	[.894]	[.809]	[.699]
(2) AI-Reported	-0.170	-0.208	-0.464	-0.035
	(0.188)	(0.228)	(0.405)	(0.104)
RI p-value	[.395]	[.394]	[.238]	[.745]
(3) AI-Generated × AI-Reported	0.145	0.158	0.336	0.041
	(0.247)	(0.300)	(0.547)	(0.151)
RI p-value (Permuting AI-Generated)	[.564]	[.62]	[.560]	[.8]
RI p-value (Permuting AI-Reported)	[.569]	[.624]	[.529]	[.809]
(4) Senior position	-0.216	-0.301	-0.509	0.123
	(0.307)	(0.363)	(0.719)	(0.193)
(5) AI-Generated × Senior position	-0.055	-0.066	-0.257	-0.151
	(0.249)	(0.300)	(0.567)	(0.152)
RI p-value (Permuting AI-Generated)	[.816]	[.811]	[.657]	[.346]
(6) AI-Reported × Senior position	0.251	0.306	0.822	0.003
	(0.258)	(0.309)	(0.580)	(0.151)
RI p-value (Permuting AI-Reported)	[.333]	[.316]	[.143]	[.978]
(7) AI-Generated × AI-Reported × Senior position	-0.350	-0.405	-0.861	-0.010
	(0.356)	(0.424)	(0.795)	(0.215)
RI p-value (Permuting AI-Generated)	[.315]	[.348]	[.286]	[.966]
RI p-value (Permuting AI-Reported)	[.331]	[.328]	[.274]	[.964]
Mean of Human-Generated and Human-Reported	2.586	.064	2.917	.679
(1) + (5): Effect of AI-Generated, Senior position = 1	-0.036	-0.035	-0.156	-0.194*
(2) + (6): Effect of AI-Reported, Senior position = 1	0.081	0.098	0.357	-0.032
(3) + (7): Effect of AI-Generated × AI-Reported, Senior position = 1	-0.205	-0.247	-0.525	0.031
(1) + (2) + (3): Total Effect of AI, Senior position = 0	-0.006	-0.019	-0.027	-0.037
(1) + (2) + (3) + (5) + (6) + (7): Total Effect of AI, Senior position = 1	-0.160	-0.184	-0.323	-0.195*
(5) + (6) + (7): Difference in Total Effect of AI, Senior position = 0 vs. Senior position = 1	-0.155	-0.165	-0.296	-0.158
Observations	366	366	366	366

Notes: * p<0.1, ** p<0.05, *** p<0.01. This table reports the heterogeneous effects of the treatments on respondents' beliefs about others' potential engagement with the blog across the five actions by whether the respondent holds a senior position (indicator equal to 1). We include this indicator as well as interact it with indicators for AI-generated blogs, AI-reported blogs, and their interaction (resulting in a triple interaction). Each action is measured using a 5-point Likert scale ranging from 'very unlikely' that others will take this action (0) to 'very likely' (4). The actions were: whether they would share the blog with others, re-read the blog, look up studies cited in the blog, look up related studies, and contact the authors. The outcome in the first column is the simple average across the five actions. The outcome in the second column is the standardized score of the first principal component, calculated based on the same five actions using Polychoric PCA. The outcome in the third column is the total number of the five actions rated as either 'likely' or 'very likely' while 'neutral', 'unlikely', and 'very unlikely' are assigned a value of 0. The outcome in the fourth column is an indicator equal to one if the respondent believes others' potential engagement is rated as either 'high' or 'very high', while 'neutral', 'low', and 'very low' are assigned a value of 0. Note that senior position cannot be permuted since it is a component of the strata. We control for wave and strata (country, seniority, and gender) fixed effects. Standard errors are clustered at the individual level (the unit of randomization).

Table S45. Beliefs about Others' Engagement by Senior Position, with Controls - Disaggregated

	(1)	(2)	(3)	(4)	(5)
	Share the blog with others	Re-read the blog	Look up studies cited	Look up related studies	Contact the authors of the brief
(1) AI-Generated	-0.141	-0.061	0.094	0.296	0.294
	(0.185)	(0.229)	(0.222)	(0.219)	(0.281)
RI p-value	[.429]	[.76]	[.686]	[.179]	[.303]
(2) AI-Reported	-0.259	-0.204	-0.061	-0.024	-0.043
	(0.239)	(0.247)	(0.209)	(0.218)	(0.302)
RI p-value	[.257]	[.394]	[.769]	[.901]	[.893]
(3) AI-Generated × AI-Reported	0.171	0.209	0.017	-0.016	0.076
	(0.301)	(0.332)	(0.293)	(0.294)	(0.381)
RI p-value (Permuting AI-Generated)	[.593]	[.551]	[.954]	[.961]	[.871]
RI p-value (Permuting AI-Reported)	[.546]	[.556]	[.954]	[.946]	[.85]
(4) Senior position	-0.108	-0.416	-0.107	-0.190	0.137
	(0.389)	(0.485)	(0.420)	(0.415)	(0.469)
(5) AI-Generated × Senior position	0.020	-0.015	-0.205	-0.182	-0.322
	(0.268)	(0.334)	(0.316)	(0.311)	(0.385)
RI p-value (Permuting AI-Generated)	[.935]	[.966]	[.508]	[.546]	[.393]
(6) AI-Reported × Senior position	0.192	0.337	0.264	0.187	0.183
	(0.319)	(0.338)	(0.308)	(0.316)	(0.392)
RI p-value (Permuting AI-Reported)	[.541]	[.309]	[.407]	[.543]	[.636]
(7) AI-Generated × AI-Reported × Senior position	-0.255	-0.339	-0.239	-0.296	-0.348
	(0.421)	(0.478)	(0.424)	(0.441)	(0.520)
RI p-value (Permuting AI-Generated)	[.548]	[.463]	[.55]	[.49]	[.502]
RI p-value (Permuting AI-Reported)	[.513]	[.441]	[.556]	[.478]	[.502]
Mean of Human-Generated and Human-Reported	2.893	2.667	2.595	2.548	2.226
(1) + (5): Effect of AI-Generated, Senior position = 1	-0.121	-0.076	-0.111	0.114	-0.028
(2) + (6): Effect of AI-Reported, Senior position = 1	-0.067	0.133	0.203	0.163	0.141
(3) + (7): Effect of AI-Generated × AI-Reported, Senior position = 1	-0.084	-0.130	-0.222	-0.312	-0.272
(1) + (2) + (3): Total Effect of AI, Senior position = 0	-0.229	-0.057	0.050	0.256	0.327
(1) + (2) + (3) + (5) + (6) + (7): Total Effect of AI, Senior position = 1	-0.272	-0.073	-0.130	-0.035	-0.159
(5) + (6) + (7): Difference in Total Effect of AI, Senior position = 0 vs. Senior position = 1	-0.043	-0.016	-0.179	-0.291	-0.486
Observations	366	366	366	366	366

Notes: * p<0.1, ** p<0.05, *** p<0.01. This table reports the heterogeneous effects of the treatments on respondents' beliefs about others' potential engagement with the blog across the five actions separately by whether the respondent holds a senior position (indicator equal to 1). We include this indicator as well as interact it with indicators for AI-generated blogs, AI-reported blogs, and their interaction (resulting in a triple interaction). Each action is measured using a 5-point Likert scale ranging from 'very unlikely' that others will take this action (0) to 'very likely' (4). An indicator variable is assigned a value of 1 if the response is either 'very likely' or 'likely' while 'neutral', 'unlikely', and 'very unlikely' are assigned a value of 0. We control for respondent characteristics, wave, and strata (country, seniority, and gender) fixed effects. Note that senior position cannot be permuted since it forms part of the strata. Standard errors are clustered at the individual level (the unit of randomization.

Table S46. Beliefs about Others' Engagement by Senior Position, without Controls - Disaggregated

	(1)	(2)	(3)	(4)	(5)
	Share the blog with others	Re-read the blog	Look up studies cited	Look up related studies	Contact the authors of the brief
(1) AI-Generated	-0.146	-0.105	-0.027	0.173	0.201
	(0.188)	(0.209)	(0.214)	(0.217)	(0.266)
RI p-value	[.448]	[.64]	[.909]	[.471]	[.466]
(2) AI-Reported	-0.242	-0.225	-0.124	-0.110	-0.149
	(0.232)	(0.238)	(0.208)	(0.211)	(0.279)
RI p-value	[.3]	[.332]	[.573]	[.623]	[.618]
(3) AI-Generated × AI-Reported	0.134	0.189	0.121	0.107	0.174
	(0.294)	(0.320)	(0.286)	(0.283)	(0.364)
RI p-value (Permuting AI-Generated)	[.655]	[.555]	[.685]	[.702]	[.668]
RI p-value (Permuting AI-Reported)	[.636]	[.564]	[.694]	[.707]	[.648]
(4) Senior position	-0.082	-0.516	-0.415	-0.526	0.460
	(0.366)	(0.373)	(0.363)	(0.396)	(0.415)
(5) AI-Generated × Senior position	-0.009	0.066	-0.010	-0.094	-0.229
	(0.272)	(0.303)	(0.301)	(0.306)	(0.360)
RI p-value (Permuting AI-Generated)	[.97]	[.83]	[.974]	[.754]	[.518]
(6) AI-Reported × Senior position	0.131	0.324	0.331	0.283	0.185
	(0.300)	(0.318)	(0.298)	(0.306)	(0.367)
RI p-value (Permuting AI-Reported)	[.692]	[.28]	[.244]	[.352]	[.572]
(7) AI-Generated × AI-Reported × Senior position	-0.196	-0.329	-0.460	-0.389	-0.376
	(0.406)	(0.447)	(0.414)	(0.420)	(0.491)
RI p-value (Permuting AI-Generated)	[.618]	[.446]	[.293]	[.358]	[.478]
RI p-value (Permuting AI-Reported)	[.64]	[.461]	[.242]	[.331]	[.45]
Mean of Human-Generated and Human-Reported	2.893	2.667	2.595	2.548	2.226
(1) + (5): Effect of AI-Generated, Senior position = 1	-0.155	-0.039	-0.037	0.079	-0.028
(2) + (6): Effect of AI-Reported, Senior position = 1	-0.111	0.099	0.207	0.173	0.036
(3) + (7): Effect of AI-Generated × AI-Reported, Senior position = 1	-0.062	-0.140	-0.338	-0.283	-0.202
(1) + (2) + (3): Total Effect of AI, Senior position = 0	-0.254	-0.140	-0.029	0.169	0.225
(1) + (2) + (3) + (5) + (6) + (7): Total Effect of AI, Senior position = 1	-0.328*	-0.080	-0.169	-0.031	-0.194
(5) + (6) + (7): Difference in Total Effect of AI, Senior position = 0 vs. Senior position = 1	-0.074	0.061	-0.139	-0.200	-0.420
Observations	366	366	366	366	366

Notes: * p<0.1, ** p<0.05, *** p<0.01. This table reports the heterogeneous effects of the treatments on respondents' beliefs about others' potential engagement with the blog across the five actions separately by whether the respondent holds a senior position (indicator equal to 1). We include this indicator as well as interact it with indicators for AI-generated blogs, AI-reported blogs, and their interaction (resulting in a triple interaction). Each action is measured using a 5-point Likert scale ranging from 'very unlikely' that others will take this action (0) to 'very likely' (4). An indicator variable is assigned a value of 1 if the response is either 'very likely' or 'likely' while 'neutral', 'unlikely', and 'very unlikely' are assigned a value of 0. We control for wave and strata (country, seniority, and gender) fixed effects. Note that senior position cannot be permuted since it forms part of the strata. Standard errors are clustered at the individual level (the unit of randomization.

Table S47. Beliefs about Others' Engagement by High Influence in Policy, with Controls

	(1)	(2)	(3)	(4)
	Others' Average Engagement Rating (0-4)	Standardized First Principal Component	Number of 'likely' or 'very likely' statements (0-5)	Others' overall rating 'high' or 'very high'
(1) AI-Generated	-0.117	-0.125	-0.187	-0.085
	(0.156)	(0.185)	(0.356)	(0.104)
RI p-value	[.429]	[.486]	[.57]	[.459]
(2) AI-Reported	-0.040	-0.050	0.002	-0.055
	(0.166)	(0.197)	(0.377)	(0.100)
RI p-value	[.816]	[.809]	[.997]	[.608]
(3) AI-Generated × AI-Reported	0.042	0.056	-0.043	0.043
	(0.227)	(0.268)	(0.521)	(0.141)
RI p-value (Permuting AI-Generated)	[.852]	[.833]	[.925]	[.755]
RI p-value (Permuting AI-Reported)	[.852]	[.83]	[.921]	[.755]
(4) High influence in policy	0.005	0.035	0.251	0.083
	(0.189)	(0.227)	(0.441)	(0.108)
RI p-value	[.978]	[.887]	[.558]	[.424]
(5) AI-Generated × High influence in policy	0.376	0.434	0.455	-0.056
	(0.267)	(0.322)	(0.594)	(0.164)
RI p-value (Permuting AI-Generated)	[.147]	[.166]	[.454]	[.727]
RI p-value (Permuting High influence in policy)	[.156]	[.18]	[.434]	[.729]
(6) AI-Reported × High influence in policy	0.094	0.119	0.146	0.056
	(0.289)	(0.346)	(0.653)	(0.160)
RI p-value (Permuting AI-Reported)	[.738]	[.734]	[.813]	[.74]
RI p-value (Permuting High influence in policy)	[.729]	[.72]	[.809]	[.733]
(7) AI-Generated × AI-Reported × High influence in policy	-0.296	-0.390	-0.286	0.006
	(0.392)	(0.468)	(0.884)	(0.233)
RI p-value (Permuting AI-Generated)	[.401]	[.357]	[.719]	[.98]
RI p-value (Permuting AI-Reported)	[.458]	[.419]	[.752]	[.975]
RI p-value (Permuting High influence in policy)	[.387]	[.343]	[.714]	[.979]
Mean of Human-Generated and Human-Reported	2.586	.064	2.917	.679
(1) + (5): Effect of AI-Generated, Influence = 1	0.259	0.309	0.268	-0.141
(2) + (6): Effect of AI-Reported, Influence = 1	0.055	0.069	0.148	0.001
(3) + (7): Effect of AI-Generated × AI-Reported, Influence = 1	-0.254	-0.334	-0.329	0.049
(1) + (2) + (3): Total Effect of AI, Influence = 0	-0.114	-0.119	-0.228	-0.097
(1) + (2) + (3) + (5) + (6) + (7): Total Effect of AI, Influence = 1	0.060	0.044	0.087	-0.091
(5) + (6) + (7): Difference in Total Effect of AI, Influence = 0 vs. Influence = 1	0.174	0.163	0.315	0.006
Observations	366	366	366	366

Notes: * p<0.1, ** p<0.05, *** p<0.01. This table reports the heterogeneous effects of the treatments on respondents' beliefs about others' potential engagement with the blog across the five actions by whether the respondent has 'high' or 'very high' influence in policy-making (indicator equal to 1). We include this indicator as well as interact it with indicators for AI-generated blogs, AI-reported blogs, and their interaction (resulting in a triple interaction). Each action is measured using a 5-point Likert scale ranging from 'very unlikely' that others will take this action (0) to 'very likely' (4). The actions were: whether they would share the blog with others, re-read the blog, look up studies cited in the blog, look up related studies, and contact the authors. The outcome in the first column is the simple average across the five actions. The outcome in the second column is the standardized score of the first principal component, calculated based on the same five actions using Polychoric PCA. The outcome in the third column is the total number of the five actions rated as either 'very likely' or 'likely' while 'neutral', 'unlikely', and 'very unlikely' are assigned a value of 0. The outcome in the fourth column is an indicator variable equal to one if the respondent believes others would rate the blog's quality as 'high' or 'very high'. We control for respondent characteristics, wave, and strata (country, seniority, and gender) fixed effects. Standard errors are clustered at the individual level (the unit of randomization).

Table S48. Beliefs about Others' Engagement by High Influence in Policy, without Controls

	(1)	(2)	(3)	(4)
	Others' Average Engagement Rating (0-4)	Standardized First Principal Component	Number of 'likely' or 'very likely' statements (0-5)	Others' overall rating 'high' or 'very high'
(1) AI-Generated	-0.135	-0.148	-0.205	-0.126
	(0.150)	(0.178)	(0.353)	(0.100)
RI p-value	[.342]	[.444]	[.536]	[.254]
(2) AI-Reported	-0.016	-0.024	0.005	-0.041
	(0.149)	(0.178)	(0.359)	(0.096)
RI p-value	[.926]	[.908]	[.992]	[.702]
(3) AI-Generated × AI-Reported	0.057	0.078	0.002	0.041
	(0.219)	(0.259)	(0.505)	(0.137)
RI p-value (Permuting AI-Generated)	[.792]	[.762]	[.998]	[.781]
RI p-value (Permuting AI-Reported)	[.772]	[.757]	[.997]	[.757]
(4) High influence in policy	0.156	0.210	0.521	0.077
	(0.191)	(0.232)	(0.449)	(0.110)
RI p-value	[.443]	[.367]	[.243]	[.48]
(5) AI-Generated × High influence in policy	0.346	0.397	0.466	0.012
	(0.271)	(0.327)	(0.612)	(0.162)
RI p-value (Permuting AI-Generated)	[.2]	[.226]	[.456]	[.942]
RI p-value (Permuting High influence in policy)	[.22]	[.214]	[.463]	[.946]
(6) AI-Reported × High influence in policy	-0.059	-0.061	-0.076	0.031
	(0.284)	(0.340)	(0.630)	(0.158)
RI p-value (Permuting AI-Reported)	[.835]	[.864]	[.912]	[.827]
RI p-value (Permuting High influence in policy)	[.83]	[.847]	[.895]	[.825]
(7) AI-Generated × AI-Reported × High influence in policy	-0.272	-0.371	-0.347	-0.031
	(0.390)	(0.465)	(0.865)	(0.229)
RI p-value (Permuting AI-Generated)	[.481]	[.39]	[.652]	[.889]
RI p-value (Permuting AI-Reported)	[.473]	[.453]	[.678]	[.876]
RI p-value (Permuting High influence in policy)	[.485]	[.38]	[.675]	[.887]
Mean of Human-Generated and Human-Reported	2.586	.064	2.917	.679
(1) + (5): Effect of AI-Generated, Influence = 1	0.210	0.249	0.261	-0.114
(2) + (6): Effect of AI-Reported, Influence = 1	-0.075	-0.085	-0.070	-0.009
(3) + (7): Effect of AI-Generated × AI-Reported, Influence = 1	-0.215	-0.293	-0.345	0.011
(1) + (2) + (3): Total Effect of AI, Influence = 0	-0.094	-0.094	-0.198	-0.125
(1) + (2) + (3) + (5) + (6) + (7): Total Effect of AI, Influence = 1	-0.079	-0.129	-0.154	-0.112
(5) + (6) + (7): Difference in Total Effect of AI, Influence = 0 vs. Influence = 1	0.015	-0.034	0.043	0.013
Observations	366	366	366	366

Notes: * p<0.1, ** p<0.05, *** p<0.01. This table reports the heterogeneous effects of the treatments on respondents' beliefs about others' potential engagement with the blog across the five actions by whether the respondent has 'high' or 'very high' influence (indicator equal to 1). We include this indicator as well as interact it with indicators for AI-generated blogs, AI-reported blogs, and their interaction (resulting in a triple interaction). Each action is measured using a 5-point Likert scale ranging from very unlikely that others will take this action (0) to very likely (4). The actions were: whether they would share the blog with others, re-read the blog, look up studies cited in the blog, look up related studies, and contact the authors. The outcome in the first column is the simple average across the five actions. The outcome in the second column is the standardized score of the first principal component, calculated based on the same five actions using Polychoric PCA. The outcome in the third column is the total number of the five actions rated as either 'likely' or 'very likely'. The outcome in the fourth column is the overall rating of others' potential engagement rated as either 'very likely' or 'likely' while 'neutral', 'unlikely', and 'very unlikely' are assigned a value of 0. The outcome in the fourth column is an indicator variable equal to one if the respondent believes others would rate the blog's quality as 'high' or 'very high'. We control for wave and strata (country, seniority, and gender) fixed effects. Standard errors are clustered at the individual level (the unit of randomization).

Table S49. Beliefs about Others' Engagement by High Influence in Policy, with Controls - Disaggregated

	(1)	(2)	(3)	(4)	(5)
	Share the blog with others	Re-read the blog	Look up studies cited	Look up related studies	Contact the authors of the brief
(1) AI-Generated	-0.285*	-0.152	-0.087	0.059	-0.118
	(0.168)	(0.205)	(0.186)	(0.189)	(0.228)
RI p-value	[.105]	[.43]	[.628]	[.769]	[.591]
(2) AI-Reported	-0.181	-0.040	-0.027	0.005	0.045
	(0.193)	(0.207)	(0.187)	(0.190)	(0.238)
RI p-value	[.353]	[.845]	[.879]	[.979]	[.86]
(3) AI-Generated × AI-Reported	0.138	0.120	-0.013	-0.059	0.024
	(0.253)	(0.295)	(0.261)	(0.266)	(0.322)
RI p-value (Permuting AI-Generated)	[.598]	[.682]	[.966]	[.838]	[.927]
RI p-value (Permuting AI-Reported)	[.635]	[.677]	[.957]	[.84]	[.949]
(4) High influence in policy	-0.025	0.095	0.092	-0.032	-0.103
	(0.204)	(0.215)	(0.234)	(0.233)	(0.297)
RI p-value	[.904]	[.651]	[.692]	[.905]	[.721]
(5) AI-Generated × High influence in policy	0.421	0.210	0.194	0.388	0.666*
	(0.297)	(0.323)	(0.321)	(0.334)	(0.398)
RI p-value (Permuting AI-Generated)	[.155]	[.522]	[.534]	[.253]	[.115]
RI p-value (Permuting High influence in policy)	[.128]	[.517]	[.559]	[.29]	[.078]
(6) AI-Reported × High influence in policy	0.029	0.005	0.290	0.173	-0.025
	(0.319)	(0.339)	(0.343)	(0.354)	(0.418)
RI p-value (Permuting AI-Reported)	[.917]	[.987]	[.415]	[.616]	[.962]
RI p-value (Permuting High influence in policy)	[.922]	[.99]	[.352]	[.611]	[.953]
(7) AI-Generated × AI-Reported × High influence in policy	-0.269	-0.230	-0.290	-0.327	-0.364
	(0.443)	(0.483)	(0.449)	(0.474)	(0.547)
RI p-value (Permuting AI-Generated)	[.538]	[.587]	[.488]	[.482]	[.462]
RI p-value (Permuting AI-Reported)	[.541]	[.619]	[.54]	[.51]	[.515]
RI p-value (Permuting High influence in policy)	[.506]	[.597]	[.502]	[.45]	[.496]
Mean of Human-Generated and Human-Reported	2.893	2.667	2.595	2.548	2.226
(1) + (5): Effect of AI-Generated, Influence = 1	0.136	0.058	0.107	0.447*	0.548*
(2) + (6): Effect of AI-Reported, Influence = 1	-0.152	-0.035	0.262	0.178	0.020
(3) + (7): Effect of AI-Generated × AI-Reported, Influence = 1	-0.131	-0.111	-0.303	-0.386	-0.340
(1) + (2) + (3): Total Effect of AI, Influence = 0	-0.328*	-0.073	-0.127	0.004	-0.049
(1) + (2) + (3) + (5) + (6) + (7): Total Effect of AI, Influence = 1	-0.147	-0.088	0.067	0.238	0.227
(5) + (6) + (7): Difference in Total Effect of AI, Influence = 0 vs. Influence = 1	0.181	-0.015	0.194	0.234	0.276
Observations	366	366	366	366	366

Notes: * p<0.1, ** p<0.05, *** p<0.01. This table reports the heterogeneous effects of the treatments on respondents' beliefs about others' potential engagement with the blog across the five actions separately by whether the respondent has 'high' or 'very high' influence in policy-making (indicator equal to 1). We include this indicator as well as interact it with indicators for AI-generated blogs, AI-reported blogs, and their interaction (resulting in a triple interaction). Each action is measured using a 5-point Likert scale ranging from 'very unlikely' that others will take this action (0) to 'very likely' (4). An indicator variable is assigned a value of 1 if the response is either 'very likely' or 'likely' while 'neutral', 'unlikely', and 'very unlikely' are assigned a value of 0. We control for respondent characteristics, wave, and strata (country, seniority, and gender) fixed effects. Standard errors are clustered at the individual level (the unit of randomization.

Table S50. Beliefs about Others' Engagement by High Influence in Policy, without Controls - Disaggregated

	(1)	(2)	(3)	(4)	(5)
	Share the blog with others	Re-read the blog	Look up studies cited	Look up related studies	Contact the authors of the brief
(1) AI-Generated	-0.277*	-0.142	-0.093	-0.001	-0.164
	(0.168)	(0.195)	(0.179)	(0.187)	(0.210)
RI p-value	[.104]	[.467]	[.585]	[.993]	[.413]
(2) AI-Reported	-0.140	0.008	0.025	0.021	0.007
	(0.188)	(0.191)	(0.173)	(0.176)	(0.217)
RI p-value	[.454]	[.976]	[.903]	[.913]	[.982]
(3) AI-Generated × AI-Reported	0.119	0.080	-0.018	0.010	0.096
	(0.251)	(0.283)	(0.249)	(0.258)	(0.302)
RI p-value (Permuting AI-Generated)	[.61]	[.775]	[.938]	[.974]	[.731]
RI p-value (Permuting AI-Reported)	[.663]	[.792]	[.945]	[.97]	[.755]
(4) High influence in policy	0.112	0.266	0.273	0.125	0.007
	(0.200)	(0.213)	(0.237)	(0.239)	(0.299)
RI p-value	[.612]	[.256]	[.241]	[.627]	[.979]
(5) AI-Generated × High influence in policy	0.351	0.190	0.161	0.343	0.684*
	(0.290)	(0.319)	(0.335)	(0.343)	(0.396)
(0.305)					
RI p-value (Permuting AI-Generated)	[.219]	[.561]	[.62]	[.296]	[.066]
RI p-value (Permuting High influence in policy)	[.209]	[.546]	[.607]	[.354]	[.071]
(6) AI-Reported × High influence in policy	-0.095	-0.176	0.088	0.059	-0.172
	(0.307)	(0.335)	(0.341)	(0.354)	(0.403)
RI p-value (Permuting AI-Reported)	[.736]	[.616]	[.791]	[.86]	[.635]
RI p-value (Permuting High influence in policy)	[.757]	[.576]	[.761]	[.85]	[.615]
(7) AI-Generated × AI-Reported × High influence in policy	-0.238	-0.158	-0.296	-0.315	-0.352
	(0.428)	(0.474)	(0.458)	(0.475)	(0.534)
RI p-value (Permuting AI-Generated)	[.563]	[.742]	[.498]	[.489]	[.494]
RI p-value (Permuting AI-Reported)	[.574]	[.762]	[.516]	[.508]	[.501]
RI p-value (Permuting High influence in policy)	[.546]	[.714]	[.506]	[.467]	[.463]
Mean of Human-Generated and Human-Reported	2.893	2.667	2.595	2.548	2.226
(1) + (5): Effect of AI-Generated, Influence = 1	0.073	0.048	0.068	0.342	0.521
(2) + (6): Effect of AI-Reported, Influence = 1	-0.235	-0.168	0.113	0.080	-0.165
(3) + (7): Effect of AI-Generated × AI-Reported, Influence = 1	-0.119	-0.078	-0.314	-0.306	-0.256
(1) + (2) + (3): Total Effect of AI, Influence = 0	-0.299	-0.054	-0.087	0.030	-0.061
(1) + (2) + (3) + (5) + (6) + (7): Total Effect of AI, Influence = 1	-0.281	-0.198	-0.133	0.117	0.100
(5) + (6) + (7): Difference in Total Effect of AI, Influence = 0 vs. Influence = 1	0.018	-0.144	-0.046	0.087	0.160
Observations	366	366	366	366	366

Notes: * p<0.1, ** p<0.05, *** p<0.01. This table reports the heterogeneous effects of the treatments on respondents' beliefs about others' potential engagement with the blog across the five actions separately by whether the respondent has 'high' or 'very high' influence in policy-making (indicator equal to 1). We include this indicator as well as interact it with indicators for AI-generated blogs, AI-reported blogs, and their interaction (resulting in a triple interaction). Each action is measured using a 5-point Likert scale ranging from 'very unlikely' that others will take this action (0) to 'very likely' (4). An indicator variable is assigned a value of 1 if the response is either 'very likely' or 'likely' while 'neutral', 'unlikely', and 'very unlikely' are assigned a value of 0. We control for wave and strata (country, seniority, and gender) fixed effects. Standard errors are clustered at the individual level (the unit of randomization).

Table S51. Beliefs about Others' Engagement by Overall Quality of Blog Rated 'High' or 'Very High', with Controls

	(1)	(2)	(3)	(4)
	Others' Average Engagement Rating (0-4)	Standardized First Principal Component	Number of 'likely' or 'very likely' statements (0-5)	Others' overall rating 'high' or 'very high'
(1) AI-Generated	-0.115	-0.129	-0.328	-0.030
	(0.194)	(0.225)	(0.501)	(0.102)
RI p-value	[.534]	[.565]	[.48]	[.788]
(2) AI-Reported	-0.259	-0.305	-0.467	0.006
	(0.214)	(0.247)	(0.523)	(0.106)
RI p-value	[.228]	[.205]	[.354]	[.966]
(3) AI-Generated × AI-Reported	0.087	0.099	0.196	-0.036
	(0.272)	(0.311)	(0.669)	(0.143)
RI p-value (Permuting AI-Generated)	[.751]	[.753]	[.759]	[.802]
RI p-value (Permuting AI-Reported)	[.757]	[.761]	[.758]	[.82]
(4) Overall quality of brief rated 'high' or 'very high'	0.144	0.181	0.356	0.639***
	(0.200)	(0.236)	(0.501)	(0.100)
RI p-value	[.477]	[.46]	[.415]	[0]
(5) AI-Generated × Overall quality of brief rated 'high' or 'very high'	0.282	0.342	0.646	0.042
	(0.243)	(0.288)	(0.608)	(0.127)
RI p-value (Permuting AI-Generated)	[.262]	[.252]	[.244]	[.803]
RI p-value (Permuting Overall quality of brief rated 'high' or 'very high')	[.315]	[.32]	[.274]	[.773]
(6) AI-Reported × Overall quality of brief rated 'high' or 'very high'	0.478*	0.563*	0.984	0.050
	(0.263)	(0.311)	(0.634)	(0.133)
RI p-value (Permuting AI-Reported)	[.069]	[.059]	[.105]	[.718]
RI p-value (Permuting Overall quality of brief rated 'high' or 'very high')	[.063]	[.068]	[.093]	[.748]
(7) AI-Generated × AI-Reported × Overall quality of brief rated 'high' or 'very high'	-0.381	-0.461	-0.857	-0.109
	(0.351)	(0.413)	(0.827)	(0.181)
RI p-value (Permuting AI-Generated)	[.289]	[.278]	[.294]	[.595]
RI p-value (Permuting AI-Reported)	[.281]	[.273]	[.304]	[.569]
RI p-value (Permuting Overall quality of brief rated 'high' or 'very high')	[.275]	[.265]	[.289]	[.589]
Mean of Human-Generated and Human-Reported	2.586	.064	2.917	.679
(1) + (5): Effect of AI-Generated, High Quality = 1	0.167	0.213	0.318	0.012
(2) + (6): Effect of AI-Reported, High Quality = 1	0.219	0.258	0.517	0.056
(3) + (7): Effect of AI-Generated × AI-Reported, High Quality = 1	-0.294	-0.362	-0.661	-0.145
(1) + (2) + (3): Total Effect of AI, High Quality = 0	-0.287	-0.335	-0.598	-0.060
(1) + (2) + (3) + (5) + (6) + (7): Total Effect of AI, High Quality = 1	0.092	0.108	0.174	-0.077
(5) + (6) + (7): Difference in Total Effect of AI, High Quality = 0 vs. High Quality = 1	0.378	0.443	0.773	-0.017
Observations	366	366	366	366

Notes: * p<0.1, ** p<0.05, *** p<0.01. This table reports the heterogeneous effects of the treatments on respondents' beliefs about others' potential engagement with the blog across the five actions by whether the respondent rated the quality of the blog as 'high' or 'very high' (indicator equal to 1). We include this indicator as well as interact it with indicators for AI-generated blogs, AI-reported blogs, and their interaction (resulting in a triple interaction). Each action is measured using a 5-point Likert scale ranging from 'very unlikely' that others will take this action (0) to 'very likely' (4). The actions were: whether they would share the blog with others, re-read the blog, look up studies cited in the blog, look up related studies, and contact the authors. The outcome in the first column is the simple average across the five actions. The outcome in the second column is the standardized score of the first principal component, calculated based on the same five actions using Polychoric PCA. The outcome in the third column is the total number of the five actions rated as either 'likely' or 'very likely' while 'neutral', 'unlike', and 'very unlikely' are assigned a value of 0. The outcome in the fourth column is an indicator variable equal to one if the respondent believes others would rate the blog's quality as 'high' or 'very high', while 'average', 'low', and 'very low' are assigned a value of 0. We control for respondent characteristics, wave, and strata (country, seniority, and gender) fixed effects. Standard errors are clustered at the individual level (the unit of randomization).

Table S52. Beliefs about Others' Engagement by Overall Quality of Blog Rated 'High' or 'Very High', without Controls

	(1)	(2)	(3)	(4)
	Others' Average Engagement Rating (0-4)	Standardized First Principal Component	Number of 'likely' or 'very likely' statements (0-5)	Others' overall rating 'high' or 'very high'
(1) AI-Generated	-0.102	-0.107	-0.236	-0.018
	(0.208)	(0.242)	(0.531)	(0.100)
RI p-value	[.588]	[.662]	[.642]	[.871]
(2) AI-Reported	-0.321	-0.370	-0.519	0.015
	(0.216)	(0.248)	(0.536)	(0.102)
RI p-value	[.146]	[.15]	[.327]	[.897]
(3) AI-Generated × AI-Reported	0.077	0.074	0.128	-0.031
	(0.287)	(0.328)	(0.688)	(0.132)
RI p-value (Permuting AI-Generated)	[.798]	[.844]	[.821]	[.814]
RI p-value (Permuting AI-Reported)	[.797]	[.835]	[.85]	[.824]
(4) Overall quality of brief rated 'high' or 'very high'	0.175	0.227	0.507	0.686***
	(0.202)	(0.238)	(0.508)	(0.095)
RI p-value	[.428]	[.383]	[.267]	[0]
(5) AI-Generated × Overall quality of brief rated 'high' or 'very high'	0.231	0.269	0.539	0.015
	(0.261)	(0.310)	(0.635)	(0.123)
RI p-value (Permuting AI-Generated)	[.36]	[.391]	[.372]	[.929]
RI p-value (Permuting Overall quality of brief rated 'high' or 'very high')	[.492]	[.514]	[.444]	[.918]
(6) AI-Reported × Overall quality of brief rated 'high' or 'very high'	0.516*	0.595*	0.929	0.043
	(0.265)	(0.312)	(0.636)	(0.126)
RI p-value (Permuting AI-Reported)	[.045]	[.042]	[.119]	[.74]
RI p-value (Permuting Overall quality of brief rated 'high' or 'very high')	[.062]	[.059]	[.119]	[.777]
(7) AI-Generated × AI-Reported × Overall quality of brief rated 'high' or 'very high'	-0.334	-0.385	-0.751	-0.141
	(0.369)	(0.434)	(0.858)	(0.172)
RI p-value (Permuting AI-Generated)	[.362]	[.388]	[.344]	[.496]
RI p-value (Permuting AI-Reported)	[.332]	[.357]	[.35]	[.428]
RI p-value (Permuting Overall quality of brief rated 'high' or 'very high')	[.407]	[.42]	[.381]	[.505]
Mean of Human-Generated and Human-Reported	2.586	.064	2.917	.679
(1) + (5): Effect of AI-Generated, High Quality = 1	0.129	0.162	0.303	-0.002
(2) + (6): Effect of AI-Reported, High Quality = 1	0.196	0.225	0.410	0.057
(3) + (7): Effect of AI-Generated × AI-Reported, High Quality = 1	-0.258	-0.312	-0.622	-0.172
(1) + (2) + (3): Total Effect of AI, High Quality = 0	-0.346*	-0.404*	-0.627	-0.034
(1) + (2) + (3) + (5) + (6) + (7): Total Effect of AI, High Quality = 1	0.067	0.075	0.091	-0.117
(5) + (6) + (7): Difference in Total Effect of AI, High Quality = 0 vs. High Quality = 1	0.413	0.479	0.718	-0.083
Observations	366	366	366	366

Notes: * p<0.1, ** p<0.05, *** p<0.01. This table reports the heterogeneous effects of the treatments on respondents' beliefs about others' potential engagement with the blog across the five actions by whether the respondent rated the quality of the blog as being 'high" or 'very high' (indicator equal to 1). We include this indicator as well as interact it with indicators for AI-generated blogs, AI-reported blogs, and their interaction (resulting in a triple interaction). Each action is measured using a 5-point Likert scale ranging from 'very unlikely' that others will take this action (0) to 'very likely' (4). The actions were: whether they would share the blog with others, re-read the blog, look up studies cited in the blog, look up related studies, and contact the authors. The outcome in the first column is the simple average across the five actions. The outcome in the second column is the standardized score of the first principal component, calculated based on the same five actions using Polychoric PCA. The outcome in the third column is the total number of the five actions rated as either 'likely' or 'very likely'. The outcome in the fourth column is an indicator variable equal to one if the respondent believes others would rate the blog's quality as 'high' or 'very high', while 'average', 'low', and 'very low' are assigned a value of zero. We control for wave and strata (country, seniority, and gender) fixed effects. Standard errors are clustered at the individual level (the unit of randomization).

Table S53. Beliefs about Others' Engagement by Overall Quality of Blog Rated 'High' or 'Very High' - Disaggregated, with Controls

	(1)	(2)	(3)	(4)	(5)
	Share the blog with others	Re-read the blog	Look up studies cited	Look up related studies	Contact the authors of the brief
(1) AI-Generated	-0.319	-0.286	-0.191	0.128	0.092
	(0.233)	(0.267)	(0.235)	(0.244)	(0.307)
RI p-value	[.191]	[.288]	[.426]	[.627]	[.749]
(2) AI-Reported	-0.481*	-0.219	-0.078	-0.087	-0.431
	(0.259)	(0.278)	(0.270)	(0.256)	(0.300)
RI p-value	[.068]	[.408]	[.785]	[.718]	[.165]
(3) AI-Generated × AI-Reported	0.097	0.042	0.076	0.050	0.172
	(0.327)	(0.377)	(0.329)	(0.333)	(0.397)
RI p-value (Permuting AI-Generated)	[.763]	[.909]	[.818]	[.882]	[.644]
RI p-value (Permuting AI-Reported)	[.757]	[.901]	[.826]	[.894]	[.694]
(4) Overall quality of brief rated 'high' or 'very high'	0.169	0.141	0.196	0.200	0.016
	(0.226)	(0.259)	(0.237)	(0.240)	(0.317)
RI p-value	[.529]	[.58]	[.401]	[.322]	[.958]
(5) AI-Generated × Overall quality of brief rated 'high' or 'very high'	0.384	0.425	0.370	0.181	0.049
	(0.275)	(0.316)	(0.294)	(0.306)	(0.385)
RI p-value (Permuting AI-Generated)	[.166]	[.217]	[.218]	[.594]	[.89]
RI p-value (Permuting Overall quality of brief rated 'high' or 'very high')	[.265]	[.288]	[.267]	[.533]	[.881]
(6) AI-Reported × Overall quality of brief rated 'high' or 'very high'	0.585*	0.348	0.301	0.314	0.841**
	(0.305)	(0.342)	(0.318)	(0.327)	(0.386)
RI p-value (Permuting AI-Reported)	[.056]	[.288]	[.361]	[.292]	[.041]
RI p-value (Permuting Overall quality of brief rated 'high' or 'very high')	[.099]	[.231]	[.292]	[.276]	[.05]
(7) AI-Generated × AI-Reported × Overall quality of brief rated 'high' or 'very high'	-0.259	-0.151	-0.450	-0.494	-0.551
	(0.417)	(0.459)	(0.416)	(0.441)	(0.523)
RI p-value (Permuting AI-Generated)	[.512]	[.779]	[.261]	[.258]	[.273]
RI p-value (Permuting AI-Reported)	[.53]	[.739]	[.332]	[.277]	[.292]
RI p-value (Permuting Overall quality of brief rated 'high' or 'very high')	[.578]	[.727]	[.233]	[.207]	[.288]
Mean of Human-Generated and Human-Reported	2.893	2.667	2.595	2.548	2.226
(1) + (5): Effect of AI-Generated, High Quality = 1	0.064	0.139	0.179	0.309	0.141
(2) + (6): Effect of AI-Reported, High Quality = 1	0.104	0.129	0.223	0.227	0.411*
(3) + (7): Effect of AI-Generated × AI-Reported, High Quality = 1	-0.162	-0.110	-0.374	-0.443	-0.379
(1) + (2) + (3): Total Effect of AI, High Quality = 0	-0.704***	-0.463*	-0.193	0.092	-0.167
(1) + (2) + (3) + (5) + (6) + (7): Total Effect of AI, High Quality = 1	0.007	0.158	0.027	0.093	0.173
(5) + (6) + (7): Difference in Total Effect of AI, High Quality = 0 vs. High Quality = 1	0.710**	0.621*	0.220	0.001	0.339
Observations	366	366	366	366	366

Notes: * p<0.1, ** p<0.05, *** p<0.01. This table reports the heterogeneous effects of the treatments on respondents' beliefs about others' potential engagement with the blog across the five actions separately by whether the respondent rated the quality of the blog as 'high' or 'very high' (indicator equal to 1). We include this indicator as well as interact it with indicators for AI-generated blogs, AI-reported blogs, and their interaction (resulting in a triple interaction). Each action is measured using a 5-point Likert scale ranging from 'very unlikely' that others will take this action (0) to 'very likely' (4). An indicator variable is assigned a value of 1 if the response is either 'very likely' or 'likely' while 'neutral', 'unlikely', and 'very unlikely' are assigned a value of 0. We control for respondent characteristics, wave, and strata (country, seniority, and gender) fixed effects. Standard errors are clustered at the individual level (the unit of randomization.

Table S54. Beliefs about Others' Engagement by Overall Quality of Blog Rated 'High' or 'Very High', without Controls - Disaggregated

	(1)	(2)	(3)	(4)	(5)
	Share the blog with others	Re-read the blog	Look up studies cited	Look up related studies	Contact the authors of the brief
(1) AI-Generated	-0.259	-0.209	-0.135	0.024	0.067
	(0.243)	(0.253)	(0.252)	(0.264)	(0.307)
RI p-value	[.275]	[.447]	[.588]	[.923]	[.82]
(2) AI-Reported	-0.475*	-0.246	-0.146	-0.191	-0.545*
	(0.258)	(0.269)	(0.273)	(0.261)	(0.283)
RI p-value	[.067]	[.322]	[.587]	[.47]	[.049]
(3) AI-Generated × AI-Reported	0.056	-0.010	-0.030	0.124	0.244
	(0.341)	(0.371)	(0.343)	(0.343)	(0.389)
RI p-value (Permuting AI-Generated)	[.861]	[.981]	[.941]	[.713]	[.525]
RI p-value (Permuting AI-Reported)	[.865]	[.981]	[.935]	[.717]	[.527]
(4) Overall quality of brief rated 'high' or 'very high'	0.244	0.228	0.204	0.153	0.045
	(0.230)	(0.247)	(0.243)	(0.246)	(0.299)
RI p-value	[.339]	[.393]	[.396]	[.479]	[.893]
(5) AI-Generated × Overall quality of brief rated 'high' or 'very high'	0.285	0.335	0.256	0.234	0.047
	(0.291)	(0.314)	(0.315)	(0.325)	(0.388)
RI p-value (Permuting AI-Generated)	[.309]	[.265]	[.419]	[.459]	[.911]
RI p-value (Permuting Overall quality of brief rated 'high' or 'very high')	[.397]	[.45]	[.452]	[.456]	[.92]
(6) AI-Reported × Overall quality of brief rated 'high' or 'very high'	0.562*	0.369	0.372	0.422	0.857**
	(0.315)	(0.335)	(0.326)	(0.326)	(0.370)
RI p-value (Permuting AI-Reported)	[.06]	[.265]	[.248]	[.182]	[.02]
RI p-value (Permuting Overall quality of brief rated 'high' or 'very high')	[.144]	[.232]	[.176]	[.146]	[.055]
(7) AI-Generated × AI-Reported × Overall quality of brief rated 'high' or 'very high'	-0.218	-0.119	-0.296	-0.494	-0.546
	(0.429)	(0.471)	(0.437)	(0.447)	(0.519)
RI p-value (Permuting AI-Generated)	[.584]	[.807]	[.498]	[.254]	[.28]
RI p-value (Permuting AI-Reported)	[.594]	[.811]	[.519]	[.255]	[.283]
RI p-value (Permuting Overall quality of brief rated 'high' or 'very high')	[.677]	[.805]	[.445]	[.246]	[.342]
Mean of Human-Generated and Human-Reported	2.893	2.667	2.595	2.548	2.226
(1) + (5): Effect of AI-Generated, High Quality = 1	0.026	0.125	0.122	0.258	0.114
(2) + (6): Effect of AI-Reported, High Quality = 1	0.087	0.123	0.226	0.231	0.312
(3) + (7): Effect of AI-Generated × AI-Reported, High Quality = 1	-0.162	-0.129	-0.326	-0.370	-0.301
(1) + (2) + (3): Total Effect of AI, High Quality = 0	-0.678***	-0.465*	-0.311	-0.043	-0.234
(1) + (2) + (3) + (5) + (6) + (7): Total Effect of AI, High Quality = 1	-0.048	0.120	0.022	0.119	0.125
(5) + (6) + (7): Difference in Total Effect of AI, High Quality = 0 vs. High Quality = 1	0.629**	0.585*	0.333	0.161	0.358
Observations	366	366	366	366	366

Notes: * p<0.1, ** p<0.05, *** p<0.01. This table reports the heterogeneous effects of the treatments on respondents' beliefs about others' potential engagement with the blog across the five actions separately by whether the respondent rated the quality of the blog as being 'high' or 'very high' (indicator equal to 1). We include this indicator as well as interact it with indicators for AI-generated blogs, AI-reported blogs, and their interaction (resulting in a triple interaction). Each action is measured using a 5-point Likert scale ranging from 'very unlikely' that others will take this action (0) to 'very likely' (4). An indicator variable is assigned a value of 1 if the response is either 'very likely' or 'likely' while 'neutral', 'unlikely', and 'very unlikely' are assigned a value of 0. We control for wave and strata (country, seniority, and gender) fixed effects. Standard errors are clustered at the individual level (the unit of randomization).

Table S55. Intended Engagement by Gender, with Controls

	(1)	(2)	(3)	(4)
	Average engagement rating (0-4)	Standardized First Principal Component	Number of 'likely' or 'very likely' statements (0-5)	Log time spent reading the blog (minutes)
(1) AI-Generated	-0.041	-0.025	-0.214	-0.039
	(0.152)	(0.179)	(0.311)	(0.269)
RI p-value	[.774]	[.883]	[.478]	[.887]
(2) AI-Reported	0.175	0.237	0.114	0.197
	(0.150)	(0.176)	(0.324)	(0.279)
RI p-value	[.238]	[.193]	[.714]	[.432]
(3) AI-Generated × AI-Reported	-0.196	-0.268	-0.182	0.057
	(0.207)	(0.243)	(0.443)	(0.363)
RI p-value (Permuting AI-Generated)	[.342]	[.276]	[.673]	[.894]
RI p-value (Permuting AI-Reported)	[.358]	[.29]	[.668]	[.858]
(4) Female	-0.870**	-0.949**	-1.800**	0.352
	(0.386)	(0.438)	(0.717)	(0.725)
(5) AI-Generated × Female	-0.422	-0.490	-0.425	0.064
	(0.277)	(0.327)	(0.540)	(0.494)
RI p-value (Permuting AI-Generated)	[.158]	[.156]	[.426]	[.898]
(6) AI-Reported × Female	-0.536*	-0.654*	-0.515	-0.017
	(0.298)	(0.351)	(0.550)	(0.614)
RI p-value (Permuting AI-Reported)	[.085]	[.078]	[.38]	[.975]
(7) AI-Generated × AI-Reported × Female	0.994**	1.191**	1.220	-0.622
	(0.416)	(0.485)	(0.769)	(0.738)
RI p-value (Permuting AI-Generated)	[.028]	[.021]	[.132]	[.456]
RI p-value (Permuting AI-Reported)	[.03]	[.029]	[.146]	[.45]
Mean of Human-Generated and Human-Reported	2.729	.085	3.25	.52
(1) + (5): Effect of AI-Generated, Female = 1	-0.464**	-0.515*	-0.639	0.026
(2) + (6): Effect of AI-Reported, Female = 1	-0.361	-0.417	-0.401	0.181
(3) + (7): Effect of AI-Generated × AI-Reported, Female = 1	0.799**	0.924**	1.038*	-0.565
(1) + (2) + (3): Total Effect of AI, Female = 0	-0.062	-0.056	-0.282	0.216
(1) + (2) + (3) + (5) + (6) + (7): Total Effect of AI, Female = 1	-0.026	-0.009	-0.002	-0.359
(5) + (6) + (7): Difference in Total Effect of AI, Female = 0 vs. Female = 1	0.036	0.047	0.281	-0.575
Observations	366	366	366	365

Notes: * $p<0.1$, ** $p<0.05$, *** $p<0.01$. This table reports the heterogeneous effects of the treatments on respondents' reported intended engagement with the blog across five actions by whether the respondent is female (indicator equal to 1). We include this indicator as well as interact it with indicators for AI-generated blogs, AI-reported blogs, and their interaction (resulting in a triple interaction). Each action is measured using a 5-point Likert scale ranging from 'very unlikely' that the respondent will take this action (0) to 'very likely' (4). The actions are: whether they would share the blog with others, re-read the blog, look up studies cited in the blog, look up related studies, and contact the authors. The outcome in the first column is the simple average across the five actions. The outcome in the second column is the standardized score of the first principal component, calculated based on the same five actions using Polychoric PCA. The outcome in the third column is the total number of the five actions rated as either 'likely' or 'very likely' while 'neutral', 'unlikely', and 'very unlikely' are assigned a value of 0. The outcome in the fourth column is the log number of minutes the respondent spent reading the blog. We control for respondent characteristics, wave, and strata (country, seniority, and gender) fixed effects. Note that gender cannot be permuted since it is a component of the strata. Standard errors are clustered at the individual level (the unit of randomization).

Table S56. Intended Engagement by Gender, without Controls

	(1)	(2)	(3)	(4)
	Average engagement rating (0-4)	Standardized First Principal Component	Number of 'likely' or 'very likely' statements (0-5)	Log time spent reading the blog (minutes)
(1) AI-Generated	-0.072	-0.068	-0.158	-0.132
	(0.152)	(0.177)	(0.314)	(0.258)
RI p-value	[.644]	[.705]	[.609]	[.62]
(2) AI-Reported	0.117	0.161	0.071	0.100
	(0.147)	(0.173)	(0.320)	(0.266)
RI p-value	[.399]	[.359]	[.815]	[.712]
(3) AI-Generated × AI-Reported	-0.160	-0.213	-0.233	0.249
	(0.208)	(0.243)	(0.444)	(0.346)
RI p-value (Permuting AI-Generated)	[.439]	[.389]	[.597]	[.474]
RI p-value (Permuting AI-Reported)	[.477]	[.361]	[.586]	[.473]
(4) Female	-0.898**	-0.988**	-1.848***	0.414
	(0.379)	(0.425)	(0.665)	(0.627)
(5) AI-Generated × Female	-0.337	-0.387	-0.440	0.043
	(0.276)	(0.323)	(0.535)	(0.460)
RI p-value (Permuting AI-Generated)	[.244]	[.287]	[.452]	[.934]
(6) AI-Reported × Female	-0.465	-0.567*	-0.336	0.016
	(0.288)	(0.339)	(0.535)	(0.608)
RI p-value (Permuting AI-Reported)	[.109]	[.103]	[.549]	[.981]
(7) AI-Generated × AI-Reported × Female	0.868**	1.033**	1.081	-0.770
	(0.401)	(0.466)	(0.735)	(0.716)
RI p-value (Permuting AI-Generated)	[.067]	[.061]	[.192]	[.333]
RI p-value (Permuting AI-Reported)	[.04]	[.035]	[.203]	[.322]
Mean of Human-Generated and Human-Reported	2.729	.085	3.25	.52
(1) + (5): Effect of AI-Generated, Female = 1	-0.409*	-0.455*	-0.598	-0.089
(2) + (6): Effect of AI-Reported, Female = 1	-0.348	-0.407	-0.265	0.116
(3) + (7): Effect of AI-Generated × AI-Reported, Female = 1	0.708**	0.820**	0.849	-0.521
(1) + (2) + (3): Total Effect of AI, Female = 0	-0.116	-0.121	-0.321	0.217
(1) + (2) + (3) + (5) + (6) + (7): Total Effect of AI, Female = 1	-0.050	-0.041	-0.014	-0.493
(5) + (6) + (7): Difference in Total Effect of AI, Female = 0 vs. Female = 1	0.066	0.080	0.306	-0.711
Observations	366	366	366	365

Notes: * p<0.1, ** p<0.05, *** p<0.01. This table reports the heterogeneous effects of the treatments on respondents' reported intended engagement with the blog across five actions by whether the respondent is female (indicator equal to 1). We include this indicator as well as interact it with indicators for AI-generated blogs, AI-reported blogs, and their interaction (resulting in a triple interaction). Each action is measured using a 5-point Likert scale ranging from 'very unlikely' that the respondent will take this action (0) to 'very likely' (4). The actions are: whether they would share the blog with others, re-read the blog, look up studies cited in the blog, look up related studies, and contact the authors. The outcome in the first column is the simple average across the five actions. The outcome in the second column is the standardized score of the first principal component, calculated based on the same five actions using Polychoric PCA. The outcome in the third column is the total number of the five actions rated as either 'likely' or 'very likely' while 'neutral', 'unlikely', and 'very unlikely' are assigned a value of 0. The outcome in the fourth column is the log number of minutes the respondent spent reading the blog. We control for wave and strata (country, seniority, and gender) fixed effects. Note that gender cannot be permuted since it is a component of the strata. Standard errors are clustered at the individual level (the unit of randomization).

Table S57. Intended Engagement by Gender, with Controls - Disaggregated

	(1)	(2)	(3)	(4)	(5)
	Share the blog with others	Re-read the blog	Look up studies cited	Look up related studies	Contact the authors of the brief
(1) AI-Generated	-0.125	-0.030	0.010	0.020	-0.081
	(0.209)	(0.206)	(0.186)	(0.175)	(0.227)
RI p-value	[.553]	[.909]	[.938]	[.913]	[.73]
(2) AI-Reported	-0.058	0.270	0.457**	0.188	0.016
	(0.197)	(0.199)	(0.180)	(0.169)	(0.230)
RI p-value	[.758]	[.175]	[.01]	[.254]	[.935]
(3) AI-Generated × AI-Reported	-0.051	-0.161	-0.470*	-0.150	-0.146
	(0.285)	(0.280)	(0.253)	(0.234)	(0.311)
RI p-value (Permuting AI-Generated)	[.852]	[.578]	[.058]	[.545]	[.619]
RI p-value (Permuting AI-Reported)	[.87]	[.582]	[.085]	[.533]	[.661]
(4) Female	-0.961*	-0.350	-0.985**	-0.961*	-1.095**
	(0.514)	(0.518)	(0.491)	(0.513)	(0.498)
(5) AI-Generated × Female	-0.570	-0.751**	-0.579*	-0.258	0.046
	(0.379)	(0.376)	(0.347)	(0.334)	(0.409)
RI p-value (Permuting AI-Generated)	[.182]	[.047]	[.114]	[.44]	[.911]
(6) AI-Reported × Female	-0.342	-1.006**	-0.933**	-0.536	0.138
	(0.383)	(0.409)	(0.397)	(0.390)	(0.406)
RI p-value (Permuting AI-Reported)	[.408]	[.013]	[.019]	[.215]	[.751]
(7) AI-Generated × AI-Reported × Female	1.051*	1.238**	1.399***	0.949*	0.335
	(0.549)	(0.572)	(0.524)	(0.496)	(0.583)
RI p-value (Permuting AI-Generated)	[.098]	[.057]	[.019]	[.069]	[.593]
RI p-value (Permuting AI-Reported)	[.105]	[.043]	[.018]	[.095]	[.546]
Mean of Human-Generated and Human-Reported	2.929	2.833	2.81	2.798	2.274
(1) + (5): Effect of AI-Generated, Female = 1	-0.695**	-0.780**	-0.570*	-0.238	-0.035
(2) + (6): Effect of AI-Reported, Female = 1	-0.400	-0.736**	-0.476	-0.348	0.155
(3) + (7): Effect of AI-Generated × AI-Reported, Female = 1	0.999**	1.077**	0.929**	0.799*	0.189
(1) + (2) + (3): Total Effect of AI, Female = 0	-0.235	0.079	-0.003	0.058	-0.211
(1) + (2) + (3) + (5) + (6) + (7): Total Effect of AI, Female = 1	-0.096	-0.439	-0.117	0.213	0.309
(5) + (6) + (7): Difference in Total Effect of AI, Female = 0 vs. Female = 1	0.138	-0.518	-0.113	0.155	0.520
Observations	366	366	366	366	366

Notes: * p<0.1, ** p<0.05, *** p<0.01. This table reports the heterogeneous effects of the treatments on respondents' reported intended engagement with the blog across the five actions separately by whether the respondent is female (indicator equal to 1). We include this indicator as well as interact it with indicators for AI-generated blogs, AI-reported blogs, and their interaction (resulting in a triple interaction). Each action is measured using a 5-point Likert scale ranging from 'very unlikely' that the respondent will take this action (0) to 'very likely' (4).An indicator variable is assigned a value of 1 if the response is either 'likely' or 'very likely' while 'neutral', 'unlikely', and 'very unlikely' are assigned a value of 0. We control for respondent characteristics, wave, and strata (country, seniority, and gender) fixed effects. Note that gender cannot be permuted since it is a component of the strata. Standard errors are clustered at the individual level (the unit of randomization).

Table S58. Intended Engagement by Gender, without Controls - Disaggregated

	(1)	(2)	(3)	(4)	(5)
	Share the blog with others	Re-read the blog	Look up studies cited	Look up related studies	Contact the authors of the brief
(1) AI-Generated	-0.206	-0.003	-0.032	0.012	-0.133
	(0.196)	(0.198)	(0.185)	(0.178)	(0.232)
RI p-value	[.31]	[.982]	[.845]	[.948]	[.575]
(2) AI-Reported	-0.055	0.307	0.357**	0.139	-0.164
	(0.187)	(0.192)	(0.175)	(0.169)	(0.231)
RI p-value	[.759]	[.105]	[.041]	[.399]	[.466]
(3) AI-Generated × AI-Reported	-0.042	-0.233	-0.392	-0.145	0.010
	(0.278)	(0.273)	(0.249)	(0.238)	(0.316)
RI p-value (Permuting AI-Generated)	[.872]	[.396]	[.101]	[.541]	[.958]
RI p-value (Permuting AI-Reported)	[.892]	[.406]	[.133]	[.531]	[.975]
(4) Gender	-0.985*	-0.356	-0.952**	-1.069**	-1.128**
	(0.536)	(0.523)	(0.479)	(0.477)	(0.458)
(5) AI-Generated × Female	-0.364	-0.700*	-0.503	-0.300	0.183
	(0.374)	(0.370)	(0.348)	(0.322)	(0.397)
RI p-value (Permuting AI-Generated)	[.376]	[.085]	[.187]	[.363]	[.66]
(6) AI-Reported × Female	-0.258	-0.973**	-0.864**	-0.467	0.235
	(0.358)	(0.402)	(0.392)	(0.368)	(0.393)
RI p-value (Permuting AI-Reported)	[.504]	[.013]	[.032]	[.25]	[.563]
(7) AI-Generated × AI-Reported × Gender	0.836	1.137**	1.257**	0.983**	0.127
	(0.532)	(0.557)	(0.518)	(0.463)	(0.549)
RI p-value (Permuting AI-Generated)	[.165]	[.08]	[.033]	[.058]	[.846]
RI p-value (Permuting AI-Reported)	[.195]	[.046]	[.025]	[.061]	[.804]
Mean of Human-Generated and Human-Reported	2.929	2.833	2.81	2.798	2.274
(1) + (5): Effect of AI-Generated, Female = 1	-0.570*	-0.703**	-0.536*	-0.288	0.050
(2) + (6): Effect of AI-Reported, Female = 1	-0.313	-0.666*	-0.507	-0.327	0.071
(3) + (7): Effect of AI-Generated × AI-Reported, Female = 1	0.794*	0.904*	0.865*	0.839**	0.137
(1) + (2) + (3): Total Effect of AI, Female = 0	-0.303	0.071	-0.068	0.007	-0.286
(1) + (2) + (3) + (5) + (6) + (7): Total Effect of AI, Female = 1	-0.090	-0.465	-0.178	0.224	0.258
(5) + (6) + (7): Difference in Total Effect of AI, Female = 0 vs. Female = 1	0.214	-0.536	-0.110	0.217	0.544
Observations	366	366	366	366	366

Notes: * p<0.1, ** p<0.05, *** p<0.01. This table reports the heterogeneous effects of the treatments on respondents' reported intended engagement with the blog across the five actions separately by whether the respondent is female (indicator equal to 1). We include this indicator as well as interact it with indicators for AI-generated blogs, AI-reported blogs, and their interaction (resulting in a triple interaction). Each action is measured using a 5-point Likert scale ranging from 'very unlikely' that the respondent will take this action (0) to 'very likely' (4). An indicator variable is assigned a value of 1 if the response is either 'likely' or 'very likely' while 'neutral', 'unlikely', and 'very unlikely' are assigned a value of 0. We control for wave and strata (country, seniority, and gender) fixed effects. Note that gender cannot be permuted since it is a component of the strata. Standard errors are clustered at the individual level (the unit of randomization).

Table S59. Beliefs about Others' Engagement by Gender, with Controls

	(1)	(2)	(3)	(4)
	Others' Average Engagement Rating (0-4)	Standardized First Principal Component	Number of 'likely' or 'very likely' statements (0-5)	Others' overall rating 'high' or 'very high'
(1) AI-Generated	0.066	0.091	-0.090	-0.114
	(0.148)	(0.176)	(0.332)	(0.092)
RI p-value	[.643]	[.597]	[.778]	[.224]
(2) AI-Reported	0.141	0.176	0.211	-0.034
	(0.147)	(0.177)	(0.348)	(0.093)
RI p-value	[.321]	[.307]	[.525]	[.723]
(3) AI-Generated × AI-Reported	-0.319	-0.394*	-0.524	-0.019
	(0.199)	(0.238)	(0.461)	(0.132)
RI p-value (Permuting AI-Generated)	[.117]	[.102]	[.261]	[.895]
RI p-value (Permuting AI-Reported)	[.092]	[.082]	[.235]	[.882]
(4) Female	-0.388	-0.511	-1.102	-0.113
	(0.366)	(0.422)	(0.852)	(0.245)
(5) AI-Generated × Female	-0.197	-0.243	0.160	0.013
	(0.266)	(0.321)	(0.613)	(0.181)
RI p-value (Permuting AI-Generated)	[.473]	[.468]	[.813]	[.949]
(6) AI-Reported × Female	-0.598*	-0.753**	-0.720	-0.027
	(0.327)	(0.379)	(0.686)	(0.167)
RI p-value (Permuting AI-Reported)	[.085]	[.053]	[.295]	[.866]
(7) AI-Generated × AI-Reported × Female	1.087**	1.319***	1.717*	0.298
	(0.430)	(0.507)	(0.934)	(0.249)
RI p-value (Permuting AI-Generated)	[.034]	[.033]	[.099]	[.331]
RI p-value (Permuting AI-Reported)	[.018]	[.016]	[.087]	[.27]
Mean of Human-Generated and Human-Reported	2.586	.064	2.917	.679
(1) + (5): Effect of AI-Generated, Female = 1	-0.130	-0.151	0.070	-0.101
(2) + (6): Effect of AI-Reported, Female = 1	-0.458	-0.577*	-0.510	-0.061
(3) + (7): Effect of AI-Generated × AI-Reported, Female = 1	0.768**	0.926**	1.193	0.279
(1) + (2) + (3): Total Effect of AI, Female = 0	-0.113	-0.126	-0.403	-0.167*
(1) + (2) + (3) + (5) + (6) + (7): Total Effect of AI, Female = 1	0.180	0.198	0.753	0.117
(5) + (6) + (7): Difference in Total Effect of AI, Female = 0 vs. Female = 1	0.292	0.324	1.156*	0.284
Observations	366	366	366	366

Notes: * p<0.1, ** p<0.05, *** p<0.01. This table reports the heterogeneous effects of the treatments on respondents' beliefs about others' potential engagement with the blog across the five actions by whether the respondent is female (indicator equal to 1). We include this indicator as well as interact it with indicators for AI-generated blogs, AI-reported blogs, and their interaction (resulting in a triple interaction). Each action is measured using a 5-point Likert scale ranging from 'very unlikely' that others will take this action (0) to 'very likely' (4). The actions were: whether others would share the blog with others, re-read the blog, look up studies cited in the blog, look up related studies, and contact the authors. The outcome in the first column is the simple average across the five actions. The outcome in the second column is the standardized score of the first principal component, calculated based on the same five actions using Polychoric PCA. The outcome in the third column is the total number of the five actions rated as either 'likely' or 'very likely' while 'neutral', 'unlikely', and 'very unlikely' are assigned a value of 0. The outcome in the fourth column is an indicator variable equal to 1 if the respondent thinks others would rate the blog's quality as 'high' or 'very high'. We control for respondent characteristics, wave, and strata (country, seniority, and gender) fixed effects. Note that gender cannot be permuted since it is a component of the strata. Standard errors are clustered at the individual level (the unit of randomization).

Table S60. Beliefs about Others' Engagement by Gender, without Controls

	(1)	(2)	(3)	(4)
	Others' Average Engagement Rating (0-4)	Standardized First Principal Component	Number of 'likely' or 'very likely' statements (0-5)	Others' overall rating 'high' or 'very high'
(1) AI-Generated	0.025	0.041	-0.134	-0.156*
	(0.151)	(0.181)	(0.348)	(0.090)
RI p-value	[.86]	[.82]	[.714]	[.096]
(2) AI-Reported	0.104	0.130	0.087	-0.046
	(0.144)	(0.174)	(0.347)	(0.090)
RI p-value	[.469]	[.464]	[.8]	[.59]
(3) AI-Generated × AI-Reported	-0.289	-0.354	-0.427	0.009
	(0.204)	(0.244)	(0.474)	(0.126)
RI p-value (Permuting AI-Generated)	[.185]	[.168]	[.368]	[.955]
RI p-value (Permuting AI-Reported)	[.154]	[.113]	[.331]	[.95]
(4) Female	-0.352	-0.459	-1.011	-0.186
	(0.358)	(0.409)	(0.804)	(0.222)
(5) AI-Generated × Female	-0.130	-0.163	0.343	0.122
	(0.263)	(0.318)	(0.595)	(0.171)
RI p-value (Permuting AI-Generated)	[.678]	[.688]	[.602]	[.524]
(6) AI-Reported × Female	-0.615**	-0.763**	-0.601	0.032
	(0.299)	(0.347)	(0.642)	(0.167)
RI p-value (Permuting AI-Reported)	[.039]	[.032]	[.33]	[.842]
(7) AI-Generated × AI-Reported × Female	1.018**	1.221**	1.338	0.118
	(0.412)	(0.484)	(0.879)	(0.242)
RI p-value (Permuting AI-Generated)	[.048]	[.056]	[.194]	[.707]
RI p-value (Permuting AI-Reported)	[.013]	[.017]	[.144]	[.659]
Mean of Human-Generated and Human-Reported	2.586	.064	2.917	.679
(1) + (5): Effect of AI-Generated, Female = 1	-0.105	-0.122	0.209	-0.035
(2) + (6): Effect of AI-Reported, Female = 1	-0.511*	-0.633**	-0.514	-0.014
(3) + (7): Effect of AI-Generated × AI-Reported, Female = 1	0.730**	0.867**	0.911	0.126
(1) + (2) + (3): Total Effect of AI, Female = 0	-0.159	-0.183	-0.474	-0.193**
(1) + (2) + (3) + (5) + (6) + (7): Total Effect of AI, Female = 1	0.114	0.112	0.606	0.078
(5) + (6) + (7): Difference in Total Effect of AI, Female = 0 vs. Female = 1	0.274	0.294	1.080*	0.271
Observations	366	366	366	366

Notes: * p<0.1, ** p<0.05, *** p<0.01. This table reports the heterogeneous effects of the treatments on respondents' beliefs about others' potential engagement with the blog across the five actions by whether the respondent is female (indicator equal to 1). We include this indicator as well as interact it with indicators for AI-generated blogs, AI-reported blogs, and their interaction (resulting in a triple interaction). Each action is measured using a 5-point Likert scale ranging from 'very unlikely' that others will take this action (0) to 'very likely' (4). The actions were: whether others would share the blog with others, re-read the blog, look up studies cited in the blog, look up related studies, and contact the authors. The outcome in the first column is the simple average across the five actions. The outcome in the second column is the standardized score of the first principal component, calculated based on the same five actions using Polychoric PCA. The outcome in the third column is the total number of the five actions rated as either 'likely' or 'very likely' while 'neutral', 'unlikely', and 'very unlikely' are assigned a value of 0. The outcome in the fourth column is an indicator variable equal to 1 if the respondent thinks others would rate the blog's quality as 'high' or 'very high'. We control for wave and strata (country, seniority, and gender) fixed effects. Note that gender cannot be permuted since it is a component of the strata. Standard errors are clustered at the individual level (the unit of randomization).

Table S61. Beliefs about Others' Engagement by Gender, with Controls - Disaggregated

	(1)	(2)	(3)	(4)	(5)
	Share the blog with others	Re-read the blog	Look up studies cited	Look up related studies	Contact the authors of the brief
(1) AI-Generated	-0.008	0.026	0.084	0.221	0.007
	(0.147)	(0.182)	(0.178)	(0.179)	(0.223)
RI p-value	[.961]	[.897]	[.611]	[.236]	[.981]
(2) AI-Reported	0.043	0.175	0.278	0.198	0.009
	(0.161)	(0.178)	(0.174)	(0.177)	(0.219)
RI p-value	[.775]	[.311]	[.104]	[.267]	[.963]
(3) AI-Generated × AI-Reported	-0.278	-0.337	-0.389*	-0.431*	-0.161
	(0.211)	(0.252)	(0.236)	(0.244)	(0.296)
RI p-value (Permuting AI-Generated)	[.212]	[.158]	[.1]	[.111]	[.597]
RI p-value (Permuting AI-Reported)	[.179]	[.169]	[.08]	[.081]	[.584]
(4) Female	-0.175	-0.132	-0.546	-0.797*	-0.292
	(0.439)	(0.439)	(0.425)	(0.406)	(0.516)
(5) AI-Generated × Female	-0.467	-0.394	-0.382	-0.119	0.378
	(0.341)	(0.339)	(0.319)	(0.329)	(0.395)
RI p-value (Permuting AI-Generated)	[.194]	[.34]	[.281]	[.725]	[.357]
(6) AI-Reported × Female	-0.832**	-0.866**	-0.844**	-0.552	0.103
	(0.394)	(0.395)	(0.358)	(0.357)	(0.451)
RI p-value (Permuting AI-Reported)	[.049]	[.034]	[.031]	[.14]	[.825]
(7) AI-Generated × AI-Reported × Female	1.309**	1.563***	1.171**	1.114**	0.280
	(0.529)	(0.527)	(0.481)	(0.467)	(0.583)
RI p-value (Permuting AI-Generated)	[.02]	[.019]	[.033]	[.05]	[.652]
RI p-value (Permuting AI-Reported)	[.026]	[.01]	[.027]	[.03]	[.617]
Mean of Human-Generated and Human-Reported	2.893	2.667	2.595	2.548	2.226
(1) + (5): Effect of AI-Generated, Female = 1	-0.475	-0.368	-0.298	0.102	0.386
(2) + (6): Effect of AI-Reported, Female = 1	-0.789**	-0.691*	-0.565*	-0.355	0.112
(3) + (7): Effect of AI-Generated × AI-Reported, Female = 1	1.030**	1.226***	0.782*	0.682*	0.119
(1) + (2) + (3): Total Effect of AI, Female = 0	-0.244	-0.136	-0.027	-0.012	-0.145
(1) + (2) + (3) + (5) + (6) + (7): Total Effect of AI, Female = 1	-0.233	0.167	-0.082	0.430*	0.616*
(5) + (6) + (7): Difference in Total Effect of AI, Female = 0 vs. Female = 1	0.011	0.303	-0.054	0.442	0.761*
Observations	366	366	366	366	366

Notes: * p<0.1, ** p<0.05, *** p<0.01. This table reports the heterogeneous effects of the treatments on respondents' beliefs about others' potential engagement with the blog across the five actions separately by whether the respondent is female (indicator equal to 1). We include this indicator as well as interact it with indicators for AI-generated blogs, AI-reported blogs, and their interaction (resulting in a triple interaction). Each action is measured using a 5-point Likert scale ranging from 'very unlikely' that others will take this action (0) to 'very likely' (4). An indicator variable is assigned a value of 1 if the response is either 'likely' or 'very likely' while 'neutral', 'unlikely', and 'very unlikely' are assigned a value of 0. We control for respondent characteristics, wave, and strata (country, seniority, and gender) fixed effects. Note that gender cannot be permuted since it is a component of the strata. Standard errors are clustered at the individual level (the unit of randomization).

Table S62. Beliefs about Others' Engagement by Gender, without Controls - Disaggregated

	(1)	(2)	(3)	(4)	(5)
	Share the blog with others	Re-read the blog	Look up studies cited	Look up related studies	Contact the authors of the brief
(1) AI-Generated	-0.064	0.030	0.044	0.152	-0.034
	(0.151)	(0.177)	(0.177)	(0.188)	(0.221)
RI p-value	[.663]	[.872]	[.803]	[.427]	[.869]
(2) AI-Reported	0.010	0.166	0.256	0.176	-0.088
	(0.159)	(0.173)	(0.169)	(0.178)	(0.214)
RI p-value	[.945]	[.337]	[.113]	[.318]	[.666]
(3) AI-Generated × AI-Reported	-0.247	-0.359	-0.393	-0.371	-0.074
	(0.215)	(0.248)	(0.238)	(0.252)	(0.291)
RI p-value (Permuting AI-Generated)	[.269]	[.143]	[.103]	[.158]	[.811]
RI p-value (Permuting AI-Reported)	[.236]	[.124]	[.096]	[.138]	[.804]
(4) Female	-0.178	-0.147	-0.531	-0.674*	-0.232
	(0.419)	(0.417)	(0.408)	(0.396)	(0.514)
(5) AI-Generated × Female	-0.312	-0.367	-0.269	-0.108	0.406
0.113					
	(0.323)	(0.342)	(0.327)	(0.316)	(0.381)
RI p-value (Permuting AI-Generated)	[.395]	[.407]	[.479]	[.769]	[.298]
(6) AI-Reported × Female	-0.761**	-0.933**	-0.862**	-0.602*	0.085
	(0.372)	(0.377)	(0.340)	(0.334)	(0.421)
RI p-value (Permuting AI-Reported)	[.043]	[.013]	[.018]	[.07]	[.851]
(7) AI-Generated × AI-Reported × Female	1.117**	1.540***	1.087**	1.114**	0.233
	(0.508)	(0.523)	(0.469)	(0.446)	(0.552)
RI p-value (Permuting AI-Generated)	[.045]	[.035]	[.057]	[.056]	[.73]
RI p-value (Permuting AI-Reported)	[.059]	[.005]	[.024]	[.017]	[.686]
Mean of Human-Generated and Human-Reported	2.893	2.667	2.595	2.548	2.226
(1) + (5): Effect of AI-Generated, Female = 1	-0.376	-0.337	-0.225	0.044	0.372
(2) + (6): Effect of AI-Reported, Female = 1	-0.752**	-0.767**	-0.606**	-0.426	-0.003
(3) + (7): Effect of AI-Generated × AI-Reported, Female = 1	0.871*	1.181**	0.694*	0.743**	0.159
(1) + (2) + (3): Total Effect of AI, Female = 0	-0.301**	-0.163	-0.093	-0.043	-0.197
(1) + (2) + (3) + (5) + (6) + (7): Total Effect of AI, Female = 1	-0.257	0.077	-0.137	0.361	0.528*
(5) + (6) + (7): Difference in Total Effect of AI, Female = 0 vs. Female = 1	0.044	0.240	-0.044	0.404	0.724**
Observations	366	366	366	366	366

Notes: * p<0.1, ** p<0.05, *** p<0.01. This table reports the heterogeneous effects of the treatments on respondents' beliefs about others' potential engagement with the blog across the five actions separately by whether the respondent is female (indicator equal to 1). We include this indicator as well as interact it with indicators for AI-generated blogs, AI-reported blogs, and their interaction (resulting in a triple interaction). Each action is measured using a 5-point Likert scale ranging from 'very unlikely' that others will take this action (0) to 'very likely' (4). An indicator variable is assigned a value of 1 if the response is either 'likely' or 'very likely' while 'neutral', 'unlikely', and 'very unlikely' are assigned a value of 0. We control for wave and strata (country, seniority, and gender) fixed effects. Note that gender cannot be permuted since it is a component of the strata. Standard errors are clustered at the individual level (the unit of randomization).

B AI Disclosure in Scientific Writing

Below is Elsevier's requirements for reporting the use of AI in writing:

Declaration of generative AI in scientific writing

The below guidance only refers to the writing process, and not to the use of AI tools to analyse and draw insights from data as part of the research process.

Where authors use generative artificial intelligence (AI) and AI-assisted technologies in the writing process, authors should only use these technologies to improve readability and language. Applying the technology should be done with human oversight and control, and authors should carefully review and edit the result, as AI can generate authoritative-sounding output that can be incorrect, incomplete or biased. AI and AI-assisted technologies should not be listed as an author or co-author, or be cited as an author. Authorship implies responsibilities and tasks that can only be attributed to and performed by humans, as outlined in Elsevier's AI policy for authors.

Authors should disclose in their manuscript the use of AI and AI-assisted technologies in the writing process by following the instructions below. A statement will appear in the published work. Please note that authors are ultimately responsible and accountable for the contents of the work.

Disclosure instructions

Authors must disclose the use of generative AI and AI-assisted technologies in the writing process by adding a statement at the end of their manuscript in the core manuscript file, before the References list. The statement should be placed in a new section entitled 'Declaration of Generative AI and AI-assisted technologies in the writing process'.

Statement: During the preparation of this work the author(s) used [NAME TOOL / SERVICE] in order to [REASON]. After using this tool/service, the author(s) reviewed and edited the content as needed and take(s) full responsibility for the content of the publication.

This declaration does not apply to the use of basic tools for checking grammar, spelling, references etc. If there is nothing to disclose, there is no need to add a statement.

C Creation of AI-generated Blogs

AI-generated policy briefs are generated through a uniform, iterative process using the following prompts:

You are a food policy researcher working at the International Food Policy Research Institute. You will write a blog post to summarize a research paper you published recently with co-authors. The main content of the blog will be a story of the research. Your target audience is thinkers and donors, and an informed general public interested in Sustainable Development Goals and food policy-related issues in developing countries. Write less formally, but not conversationally. Avoid jargon. Use the tone as if you are explaining research to a colleague from a different background. Don't repeat the same thought. Start the blog with a headline, which is descriptive and suggestive of the relevant findings for readers get a sense of what they are getting into. Outline the question this document addresses, and the approach pursued. Frame the main point quickly, in paragraph or two. Include noteworthy details, such as challenges faced in the field. Explain the findings and their significance. Use bullet points to present policy recommendations. Conclude by reflecting on what your research shows about the broader issue, or directions for future research. Avoid repeating points already made. Don't use subheadings. Use the active voice. Use a less formal but still professional tone. Avoid using adverbs or adjectives. If you understood this requirement, answer "Understood". You don't have to draft the blog at this time.

Let's start with the headline. Can you give me a good headline for the blog?

Good! Now, write me the introduction. Without using a section heading, the introduction should set up the rest of the blog and clearly convey the argument of the document. In two paragraphs, clearly describe the challenges faced. Define why you are writing the blog and express the urgency and importance of the topic. This in-

troduction should contain all of the information relevant to the title of the document and the main objectives of the research, based on the key questions. Do not use "the study," "the research," or "in conclusion" and make this section as a standalone text that can be read and understood assuming readers do not have access to the whole PDF document.

Looks good! Let's move on to the Research Overview section. Without using a section heading, analyze the uploaded PDF document and draft the section to provide a summary of the research that describe the issues, contexts, data, and research methods in two paragraphs. In the first paragraph, explain the purpose of the research, how the study was conducted, how the data was collected and used, and any other relevant background information. In the second paragraph, explain the research results first and moving on to the specific research findings. Do not use "the study," "the research," or "in conclusion" and make this section as a standalone text that can be read and understood assuming readers do not have access to the whole PDF document. Use a less formal but still professional tone.

Looks good! Let's move on to the Key Findings section. Without using a section heading, this section should interpret the data in a way that is accessible and clearly connected to the policy recommendations. The goal is to be convincing, but ensure that the analysis is balanced and defensible. Explain the key research findings clearly and comprehensively. Use the active voice primarily. Provide a high-level highlight of the key findings in one sentence at the beginning, followed by presenting key findings one by one. Do not use "the study," "the research," or "in conclusion" and make this section as a standalone text that can be read and understood assuming readers do not have access to the whole PDF document. Use a less formal but still professional tone.

Looks good! Let's move on to the Policy Recommendations section. Detail the actions recommended by the research findings. Draw the link between the Research Findings and the Policy Recommendations. Use persuasive language to present each of the recommendations. Try to completely convince readers that the presented Policy Recommendations are the best advice. Describe the potential consequences of implementing particular policies. Include the consequences of inaction as well if found in the PDF document. Recommendations should act as a call to action by stating precise, relevant, credible, and feasible next steps. Provide a high-level highlight of the key findings in one sentence at the beginning, followed by presenting key findings one by one in about five bullet points. Do not use "the study," "the research," or "in conclusion" and make this section as a standalone text that can be read and understood assuming readers do not have access to the whole PDF document. Use a less formal but still professional tone.

Looks good! Finally, let's add a Conclusion section. In one short paragraph of text without using bullet points, briefly summarize the Policy Recommendations recommended by the Key Research Findings. Explain the reasons behind the Policy Recommendations. The conclusion section should link the research findings to the policy recommendations. End the section with a concluding statement that reiterates the key message and suggests the significance of the proposed Policy Recommendations. Do not use and make this section as a standalone text that can be read and understood assuming readers do not have access to the whole PDF document. Use a professional tone at the same level used in the PDF document. Use the passive voice primarily.

D Text of Emails and Social Media Posts

D.1 Email Invitation to Survey

SUBJECT LINE: Invitation: Help improve research-based policy recommendations for [country].

Greetings from IFPRI.

As part of our ongoing efforts to provide research-based policy solutions in [country], we invite you to participate in an important survey. We expect that your participation in the survey will make a significant contribution to improve the role of science in policy making The survey will take about 20 minutes to complete and you will have

a chance to win 100 USD by taking the survey. To respect your privacy and adhere to General Data Protection Regulation (GDPR) guidelines, we assure you that your participation is entirely voluntary, and your responses will be anonymous. By clicking the survey link below, you will be guided to the survey form:

[*Link to Survey*]

If you have any concerns about your data privacy or would like to learn more about how we handle your information, please review our Privacy & Cookie Policy or contact our Data team IFPRI-Data@CGIAR.ORG.

Sincerely,
[Country lead name and position]

D.2 X post

Passionate about research-based policymaking and better communication with policymakers? Join our survey and make an impact!

https://t.co/8OA3YdSBRn

Choose your country and participate for a chance to win 100!

@IFPRI #NPSInitiative #DigitalInnovationInitiative

D.3 LinkedIn

Are you keen in promoting research-based policymaking and contribute to our understanding what role Artificial Intelligence might play in this? Then please select one country that you are professionally most engaged with and participate in our survey. The survey will take about 20 minutes to complete and you will have a chance to win 100 USD by taking the survey. Kenya Egypt Bangladesh Ethiopia Ghana India Malawi Nigeria Rwanda Sudan Tajikistan Uganda . The survey is led by #IFPRI under the CGIAR Initiatives on National Policies and Strategies #NPSInitiative and Digital Innovation Initiative #DigitalInnovationInitiative.

Acknowledgments

We thank James Allen IV, Kate Ambler, Todd Benson, Jessica Leight, Carly Trachtman, and participants from the IFPRI RISE and IFPRI brownbag seminar series for valuable suggestions. We also thank IFPRI country office leads for Bangladesh, Egypt, Ethiopia, Ghana, India, Kenya, Malawi, Nigeria, Rwanda, Sudan, and Uganda, as well as Michael Go, and Omar Fares, and the participants of the study.

Declaration of generative AI and AI-assisted technologies in the writing process

During the preparation of this work the author(s) used ChatGPT-4 in order to create the experiment and to check for grammar. After using this tool/service, the authors reviewed and edited the content as needed and take full responsibility for the content of the publication.
