# Supplementary material for: Man vs. machine: Multi-country experimental evidence on the quality and perceptions of AI-generated research blog content
Source: PLoS One. 2026 Mar 25;21(3):e0342852. doi: 10.1371/journal.pone.0342852 (PMC13016282; doi:10.1371/journal.pone.0342852)
Supplement: Attachment — Submitted filename: responses_to_reviewers.pdf [file pone.0342852.s005.pdf]

Editor's Comments

I read the manuscript "Man vs. Machine: Global experimental evidence on the quality and perceptions of AI generated research content" with great interest, since it deals with the perceived quality of professional content, evaluated by educated readers, in a very specific context. The analysis is thorough and timely, since AI vs human-generated topic represents a highly debated topic both in scientific and in general articles.

The authors set the background in a legitimate manner, engaging with relevant literature, which is presented in a clear and convincing style. The structure of the manuscript is logical and presented in a transparent manner, early in the article and followed through up to the end.

Thank you for your kind words! We very much enjoyed working on this project and hope to do some more work as extensions. The referees gave us some good ideas in this regard.

The data obtained in the experiment is presented fairly and sustains the proposed theme of the article. Personally, I would have liked a corpus having scientific articles accompanied by blogs authored by the same (groups of) researchers, as one sample, and scientific texts accompanied by AI generated blogs as another, but I understand that the logic behind the experiment dictated a different approach. Also, I would have liked to see in the survey a stronger discussion on the engagement of readers with the content (blogs and original research articles that the blogs summarize or popularize). However, the authors of the manuscript explained their endeavors quite legitimately and it can be accepted as such. I particularly signal the fact that the experiment was carried out across a significant number of countries and in four waves, factors that ensure a representativeness of data.

Yes, it would have been great to have more information on engagement. We have noted this in the paper, as one of the referees also mentioned this. Perhaps if we can do a follow-up study, we can include this.

An aspect that I would suggest for strengthening the manuscript is for the authors to acknowledge the limitations of their study and to better announce the possibility of further studies and implications of their findings.

We have now included a discussion section instead of a conclusion and in the discussion section we have noted strengths and limitations of the study and elaborated on potential further research that either we plan to do or that the referees have suggested. All our updated text is copied below.

The figures in the manuscript are sufficient and help understanding the data. Also the appendices are useful, containing valuable information, while not overloading the manuscript with elements that could hinder the flow of the article.

In conclusion, I believe that the manuscript is of publishable quality, with minor revisions that can be easily carried out in the preparation for final editing.

Again, thank you. We have updated the article in response to the referee comments and to reflect all the journal requirements. Our responses appear below each referee comment in italics, and any updated or added text is quoted as well.

Journal Requirements:

When submitting your revision, we need you to address these additional requirements.

1. Please ensure that your manuscript meets PLOS ONE's style requirements, including those for file naming. The PLOS ONE style templates can be found at

https://journals.plos.org/plosone/s/file?id=wjVg/PLOSOne_formatting_sample_main_body.pdf and

https://journals.plos.org/plosone/s/file?id=ba62/PLOSOne_formatting_sample_title_authors_affiliations.pdf

2. PLOS requires an ORCID iD for the corresponding author in Editorial Manager on papers submitted after December 6th, 2016. Please ensure that you have an ORCID iD and that it is validated in Editorial Manager. To do this, go to 'Update my Information' (in the upper left-hand corner of the main menu), and click on the Fetch/Validate link next to the ORCID field. This will take you to the ORCID site and allow you to create a new iD or authenticate a pre-existing iD in Editorial Manager.

The corresponding author has followed the instructions included the information on ORCID in Editorial Manager. In case there are still issues, the ORCID can be found here: Michael Keenan (0000-0001-5191-9146) - ORCID.

3. Thank you for stating the following financial disclosure:
This work was supported by the CGIAR Initiatives on National Policies and Strategies (NPS) and Digital Innovation (DI) and the Bill & Melinda Gates Foundation through the Generative AI for Agriculture project (INV-047346).

Please state what role the funders took in the study. If the funders had no role, please state: "The funders had no role in study design, data collection and analysis, decision to publish, or preparation of the manuscript."
If this statement is not correct you must amend it as needed.
Please include this amended Role of Funder statement in your cover letter; we will change the online submission form on your behalf.

We have added the above quote stating the funders played no role. Thank you for updating it on the online submission form.

4. Your ethics statement should only appear in the Methods section of your manuscript. If your ethics statement is written in any section besides the Methods, please delete it from any other section.

We now refer to the IRB approval only in Section 3: Methods.

5. When completing the data availability statement of the submission form, you indicated that you will make your data available on acceptance. We strongly recommend all authors decide on a data sharing plan before acceptance, as the process can be lengthy and hold up publication timelines. Please note that, though access restrictions are acceptable now, your entire data will need to be made freely accessible if your manuscript is accepted for publication. This policy applies to all data except where public deposition would breach compliance with the protocol approved by your research ethics board. If you are unable to adhere to our open data policy, please kindly revise your statement to explain your reasoning and we will seek the editor's input on an exemption. Please be assured that, once you have provided your new statement, the assessment of your exemption will not hold up the peer review process.

We have made the data available on the IFPRI Dataverse at: https://doi.org/10.7910/DVN/GETDGX

6. Please amend your list of authors on the manuscript to ensure that each author is linked to an affiliation. Authors' affiliations should reflect the institution where the work was done (if authors moved subsequently, you can also list the new affiliation stating "current affiliation:…." As necessary).

Apologies for this oversight, we have updated the list of authors' affiliations.

7. Please include captions for your Supporting Information files at the end of your manuscript, and update any in-text citations to match accordingly. Please see our Supporting Information guidelines for more information: http://journals.plos.org/plosone/s/supporting-information.

We do not have any supporting information that links to in-text citations.

8. Please remove all personal information, ensure that the data shared are in accordance with participant consent, and re-upload a fully anonymized data set.

Note: spreadsheet columns with personal information must be removed and not hidden as all hidden columns will appear in the published file.

Additional guidance on preparing raw data for publication can be found in our Data Policy (https://journals.plos.org/plosone/s/data-availability#loc-human-research-participant-data-and-other-sensitive-data) and in the following article: http://www.bmj.com/content/340/bmj.c181.long.

We have removed all personally identifiable information (PII) from the data that has been posted to IFPRI's Dataverse and we confirm that the PII is not available anywhere. The link to the data is: https://doi.org/10.7910/DVN/GETDGX.

9. If the reviewer comments include a recommendation to cite specific previously published works, please review and evaluate these publications to determine whether they are relevant and should be cited. There is no requirement to cite these works unless the editor has indicated otherwise.

Thank you for the guidance.

Additional Editor Comments:

Title is too broad. It does not specify that the medium studied is blogs nor that the design is cross-country survey experiment.

Thank you for noting this. The study is not so much cross-country comparisons but is a multi-country sample. Consequently, we suggest the title below:

"Man vs. Machine: Multi-Country experimental evidence on the quality and perceptions of AI-generated research blogs"

Extend the abstract section and summarize aim, methods, results, and discussion (without subheadings).

We appreciate this suggestion to flesh out the abstract. It now reads:

"Academic research is not always available in a form that is accessible or engaging to a non-academic audience, hindering readers' engagement with it. Non-academics, even if highly educated and policy experts in their fields, tend to need research to be presented in a more accessible way than peer-reviewed articles --- one example being non-technical blogs. However, writing these requires effort from researchers. Artificial Intelligence (AI) tools can make academic research easier to understand by summarizing and simplifying academic papers much more quickly than researchers can, making it easier for researchers to produce such summaries. However, disclosure of AI use may lower readers' perceived quality of and trust in the blog, generating a tradeoff for the researcher. In this paper, we evaluate an 11-country experiment cross-randomizing a blog's actual and reported author as AI or human. We find that research stakeholders rate the quality of AI-generated blogs marginally lower than human-written ones ($p < 0.1$), but disclosure of AI use offsets the negative effect ($p < 0.1$). The sample in this study, while being the policy relevant sample who would engage with such policy-oriented academic research, is one that is highly educated and includes some thematic specialists. Indeed, findings indicate that their view of "accessibility" of research is such that they prefer slightly more technical summaries of research. The nature of the respondents may thus explain the particular findings in this study, suggesting that researchers should tailor their prompts for their intended audience. There are no effects on readers' reported likelihood of engaging with the blog or on beliefs about others predicted engagement with it. Consequently, we hypothesize that researchers can leverage AI to communicate their research more easily without a penalty from disclosing its use."

In several places, the manuscript cites references by number only (e.g., "the seminal paper by [15]"). Author names should be included where appropriate, e.g., "The seminal paper by Akerlof GA [15]."

Thank you for pointing this out, and apologies for the oversight. The author names have now been included where appropriate.

Methodological details were spread between the Introduction and the Experiment Design section. Please consolidate into a standalone Methods section, following standard IMRAD structure. This section should include study design and population, outcomes measured and statistical analysis plan.

Thank you for this suggestion. We have re-structured the Methods section to reflect that of the IMRAD structure, which has made it read better.

Specify the method, algorithm, or tool employed for randomization.

We have added details on the tool and method employed on page 11 (<u>**NOTE:**</u> the page numbers are those in the *new, clean* version of the paper, not the tracked changes version) *as follows:*

"Participants were assigned to treatment groups within the online survey that used the program SurveyCTO. The method used was systematic, stratified randomization."

Which software used for data analysis? Add with details at the Methods section.

We have specified that we used Stata 18 for our analysis (page 13).

AI-generated blogs were rated lower in quality despite being more accessible. I suggest examining potential bias against AI alongside textual features (e.g., tone, jargon, stylistic markers) where AI outputs may differ from human blogs. Such analysis could clarify why readers perceive AI texts as lower quality.

This would have been a great insight to dig into further. We did examine two potential sources of bias. One potential bias we examined is whether people had not previously used AI (potentially leading to bias because they are unfamiliar with it) and the other is whether people trust AI. We had reported in the paper that people who reported previously having used AI do display differential impacts, with truthful revelations being more highly valued by them. Interestingly, there were no differences in perceived quality or engagement by whether the respondent reports trusting AI. So, with the data we have, we can conjecture that people may have biases stemming from being unfamiliar with the technology. We have done so on page 19 in the results for prior AI use where we say:

“This result could point to a behavioural bias against AI as this unfamiliarity increases ambiguity.”

Consider including one figure that directly summarizes the main result e.g., a concise visualization comparing perceptions of quality across the four experimental arms.

We agree that “a picture is worth a thousand words” and have thus summarized the main results in Figure 1 on the left-hand side panels. The primary result is the one on the left in Panel A. We have now emphasized it more in the text on page 17:

“The left-hand side of Panel (a) summarizes the main result of the paper.”

I recommend separating out a full Discussion section. This will allow a fuller exploration of:
• Interpretation of findings.
• Limitations (e.g., highly educated sample, external validity).
• Implications for research communication and directions for future research.

This was a recommendation from other referees as well, and we have now included a Discussion section with a detailed interpretation of findings, strengths and limitations of the study, and suggestions for future research.

Reviewers' comments:

Reviewer's Responses to Questions

Comments to the Author

1. Is the manuscript technically sound, and do the data support the conclusions?

The manuscript must describe a technically sound piece of scientific research with data that supports the conclusions. Experiments must have been conducted rigorously, with appropriate controls, replication, and sample sizes. The conclusions must be drawn appropriately based on the data presented.

Reviewer #1: Partly

Reviewer #2: Yes

Reviewer #3: Yes

2. Has the statistical analysis been performed appropriately and rigorously?

Reviewer #1: Yes

Reviewer #2: Yes

Reviewer #3: Yes

3. Have the authors made all data underlying the findings in their manuscript fully available?

The PLOS Data policy requires authors to make all data underlying the findings described in their manuscript fully available without restriction, with rare exception (please refer to the Data Availability Statement in the manuscript PDF file). The data should be provided as part of the manuscript or its supporting information, or deposited to a public repository. For example, in addition to summary statistics, the data points behind means, medians and variance measures should be available. If there are restrictions on publicly sharing data—e.g. participant privacy or use of data from a third party—those must be specified.

Reviewer #1: Yes

Reviewer #2: Yes

Reviewer #3: Yes

4. Is the manuscript presented in an intelligible fashion and written in standard English?

PLOS ONE does not copyedit accepted manuscripts, so the language in submitted articles must be clear, correct, and unambiguous. Any typographical or grammatical errors should be corrected at revision, so please note any specific errors here.

Reviewer #1: Yes

Reviewer #2: Yes

Reviewer #3: Yes

5. Review Comments to the Author

Please use the space provided to explain your answers to the questions above. You may also include additional comments for the author, including concerns about dual publication, research ethics, or publication ethics. (Please upload your review as an attachment if it exceeds 20,000 characters)

Reviewer #1: General Comments

This manuscript presents a well-designed 2x2 factorial experiment investigating how AI-generated versus human-written research blogs, and the disclosure of their authorship, affect perceptions of quality and engagement among research consumers. The study is timely, addresses a highly relevant topic in academic communication, and is grounded in a solid theoretical framework of information asymmetry. The methodology is clearly described, including the pre-registration and IRB approval, and the statistical analysis is appropriate. The paper is exceptionally well-written and logically structured.

Thank you for these kind words!

However, the study has two major weaknesses that require substantial revision, along with a few minor points that would further strengthen the manuscript.

Major Weaknesses:

Sample Generalizability: The manuscript's primary limitation is its reliance on a convenience sample drawn from IFPRI mailing lists and social media, which consists of highly educated specialists (over 80% with a master's degree or higher). While the authors acknowledge this, the paper's framing and language frequently generalize the findings to broader populations (e.g., "readers," "the informed general public"). The study's conclusions are only directly applicable to a very specific, elite group of research consumers. The manuscript must be systematically revised to consistently frame the context, interpretation, and implications of the findings around this specific demographic. This should be explicitly stated in the Abstract, Methods, Discussion, and Conclusion to avoid overstating the external validity of the results.

This is a good point, and we have updated the text throughout the manuscript to be more precise and refer either to just the “respondents” or “this sample.” We have also re-framed

some of the interpretation of the results and have emphasized the point that the nature of this sample is potentially the main driver of these particular results (closely related to your second point, below). We also note that this sample, while a convenience sample, is the policy relevant sample for this study because they are the ones most interested in IFPRI research. Consequently, researchers should target their products (and thus their prompts) to ensure they are reaching the right audience for their work.

*On page 26 (***<u>NOTE:</u>** the page numbers are those in the *new, clean* version of the paper, not the tracked changes version) *we say:*

"while the sample is relevant and lends internal validity, it is a convenience sample and does not represent all types of users who would find the type of evidence produced relevant; our sample is highly educated and connected to research and policy and does not include journalists, for example. In fact, the demographics of our sample, particularly high levels of education, is likely the most important driver of our result. Consequently, these results are most applicable for researchers who would like to communicate their research to stakeholders who are technically advanced compared to the general public."

Interpretation of Accessibility: The study uses the Flesch-Kincaid grade level as an "objective measure of accessibility" and finds that AI-generated blogs are more "accessible" (i.e., have a lower grade level). However, for the highly specialized audience surveyed, a lower reading level may not be perceived as a positive attribute. It could instead signal a lack of technical nuance, oversimplification, or lower scientific rigor. The assumption that greater simplicity equates to higher quality is a critical conceptual issue for this specific sample. The Discussion section should more deeply explore this alternative interpretation as a primary mechanism for the findings, rather than just a penalty for ambiguity.

Thank you for noting this. We did note this in the results section, but we have expanded this in the discussion section on page 26 where we say:

"…the Flesch-Kincaid model was developed for English speakers in the US and may not be as good of a measure in contexts where English is not the first language. Additionally, as the results indicate, our sample of highly-educated readers may not value the oversimplicity that AI may generate."

On page 25 we also say:

"Our highly educated sample (80% with a master's or higher and with technical expertise in the subject matter) may view accessibility differently, favoring technical language and detail. Their preferred blog length exceeded the actual length, suggesting preferences

regarding accessibility depend on the audience. A lower reading level may also signal weaker research quality, as academic language often conveys rigor. Researchers should thus tailor their AI prompts accordingly."

As a limitation in the methods section, we also include, on page 13:

"a limitation is that it was developed for American English and thus may contain some measurement error."

Minor Weaknesses:

Justification for Statistical Model: The analysis relies on OLS for ordinal outcomes (Likert scales). While this is common practice, a brief justification for this choice over models designed for ordinal data (e.g., ordered logit) would strengthen the statistical methodology section. A robustness check using such a model could also be beneficial.

Thank you for noting this, as it is a methodologically important point. While the questions we asked respondents are indeed ordinal on a Likert scale, the outcomes we use in our regressions are mostly indices of these questions (either weighted or unweighted averages, or a principal components analysis). Below are histograms of the outcomes. Due to the averaging, most of the variables go from 0-4 but not in clear discrete steps. These outcomes resemble a continuous variable instead. There are two outcomes, the sum of likely or very likely responses to statements for own engagement and for others' engagement that can be considered ordinal. See histograms below.

Quality:

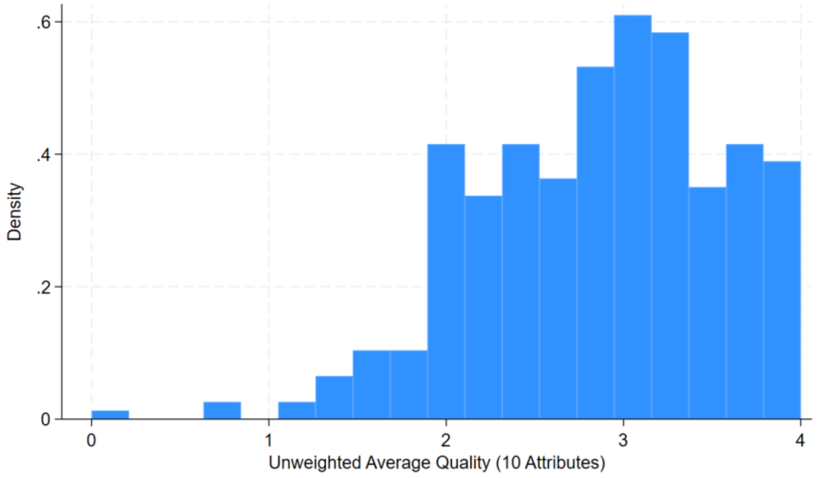

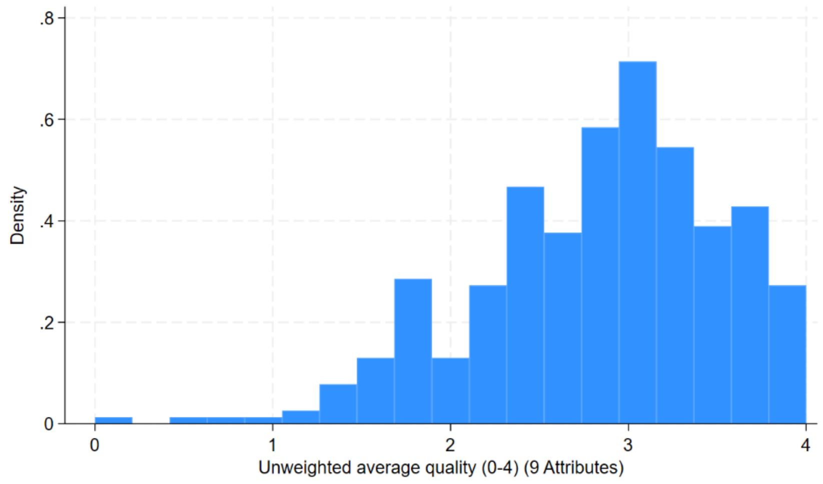

Density
Unweighted average quality (0-4) (9 Attributes)

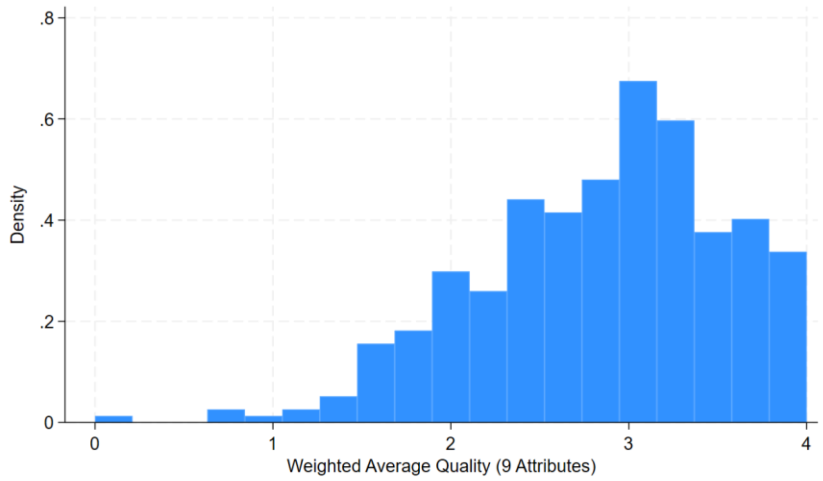

Density
Weighted Average Quality (9 Attributes)

Engagement:

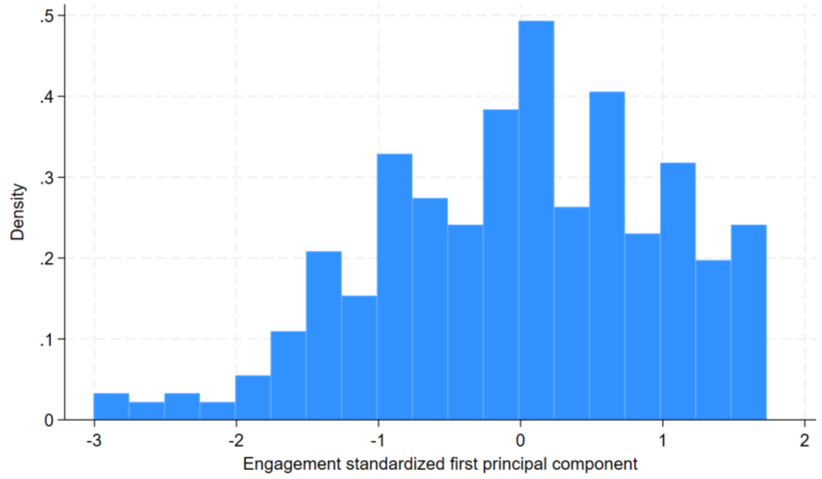

Density
Engagement standardized first principal component

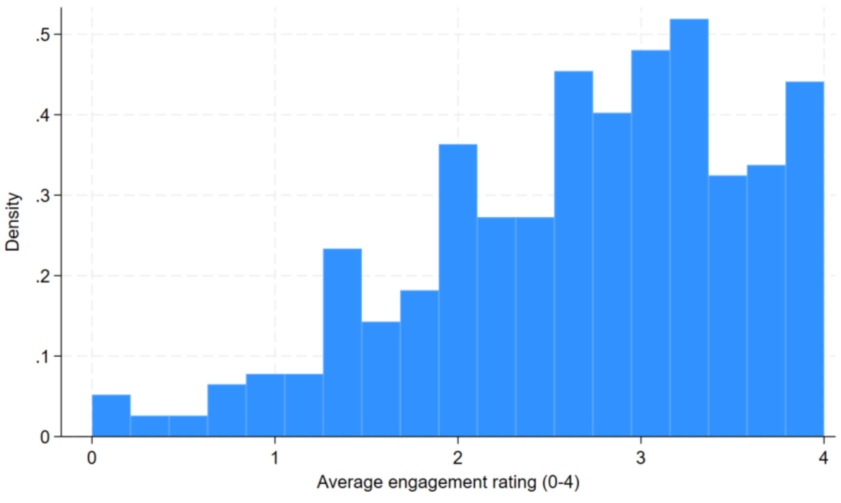

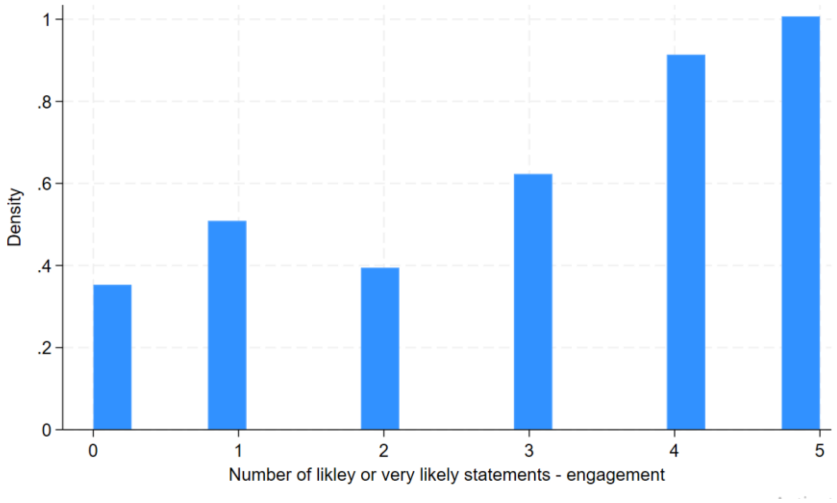

Others' engagement:

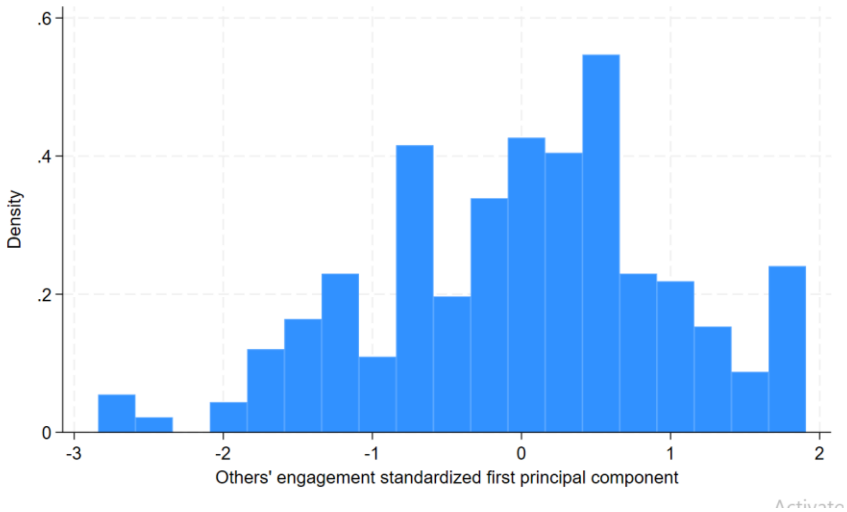

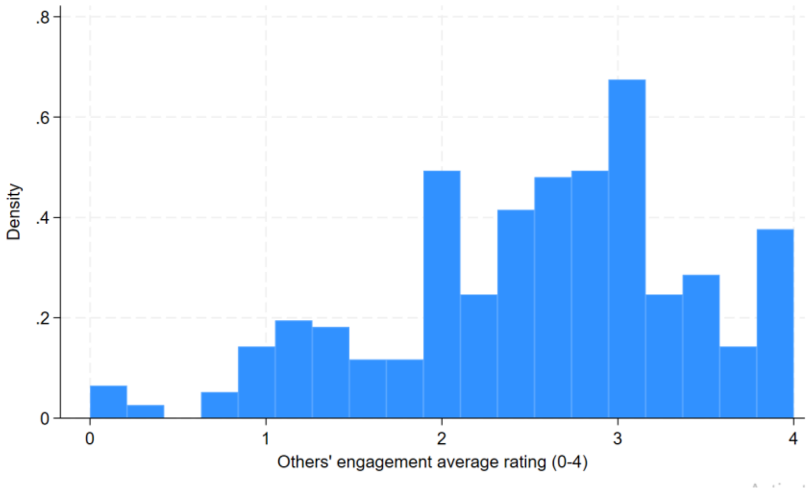

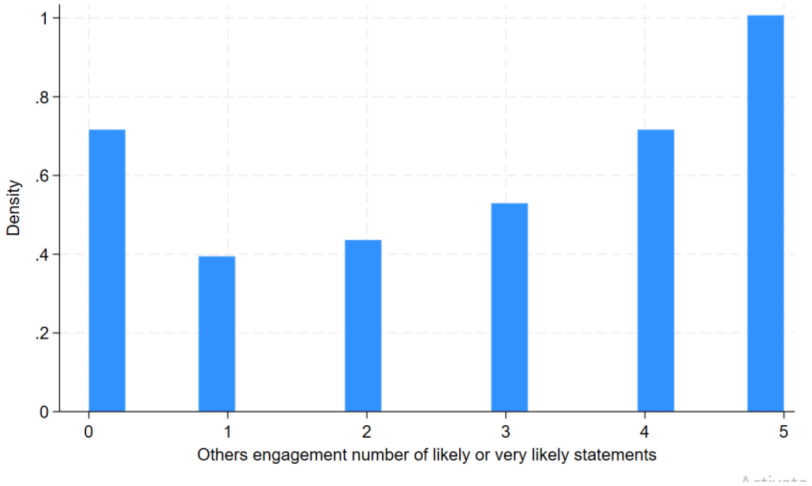

On page 14, we explain our reasoning:

"There are four reasons that we use OLS for all of our outcomes despite the sums of engagement behaviours being ordinal. First, our primary interest is to compare differences in ***means*** *rather than the probability of being in a particular category. Second, when there are 2-7 categories, OLS often fares just as well as ordered logit (Robitzsch, 2020; Robitzsch, 2022). Third, in specifications with interactions (in our case, there are triple interactions), the interpretation of the odds ratios from ordered logit is extremely difficult. Fourth, we preferred to keep the specifications consistent over the outcomes so that they can be more comparable."*

Cross-Cultural Validity of Readability Metric: The Flesch-Kincaid score was developed for American English. Its application across a diverse sample from 11 countries, where English is often not the first language, is a limitation. While acknowledged, this point could be briefly noted in the methodology section as a potential source of measurement error.

Thank you for this important point. We have included this limitation in the methodology section (page 13) where we say:

"While a limitation is that it was developed for American English and thus may contain some measurement error, it serves as our best available metric for text accessibility in this context."

and we note this in the discussion section (page 25) as quoted above.

Power for Subgroup Analyses: The heterogeneity analyses, particularly concerning gender, are intriguing . However, with a total sample of 366, the statistical power for these subgroup comparisons may be limited. A brief comment on the exploratory nature of these findings or a post-hoc power analysis would be appropriate.

Thank you, this is an important point. On page 18 we have added:

"We first note that given our relatively small sample size, statistical power could potentially be limited. Ex-post power calculations suggest that we are reasonably powered in the quality outcomes,and thus, to the extent that we may be sufficiently powered to detect some differential effects, we attempt such an analysis."

We have also conducted post-hoc power analysis. The following are our MDEs for our main outcomes (assuming power of 0.8, p 0.05, means and standard deviations of the sample, and 366 observations divided across 8 groups (to account for the interaction of 4 groups plus the variable used for heterogeneity analysis):

Quality:

- *Unweighted average 10 attributes: 0.40 points (on the scale of 0-4)*
- *Unweighted average 9 attributes: 0.41 points*
- *Weighted average 10 attributes: 0.41 points*

Engagement:

- *Engagement PCA score: 0.59 standard deviations*
- *Engagement unweighted average (0-4): 1.56 points*
- *Engagement sum (0-5): 0.98 points*

Others' engagement:

- *Others' engagement PCA score: 0.59 standard deviations*
- *Others' engagement unweighted average (0-4): 0.49 points*

- *Others' engagement sum (0-5): 1.1 points*

We are thus reasonably powered for the quality outcomes, but less so for the engagement and others' engagement outcomes. Consequently, we note your point that this is still exploratory and have added details about power in the methods section.

Specific Comments

Page 1, Abstract: The phrase "readers rate the quality" should be specified to better reflect the sample, for instance, "research stakeholders rate the quality...". This clarifies the population from the outset.

We have amended the wording in the abstract.

Page 11, Section 3.1, Paragraph 3: The statement that the online survey was distributed to reach a "variety of stakeholders" should be more precise. The description that follows indicates a rather homogenous group of highly educated professionals in specific fields. Please rephrase to accurately reflect the sample's characteristics.

This is a good point. We have updated the wording to "a large set of stakeholders."

Page 12, Section 4.2, Paragraph 1: The statement "truthful disclosure of AI use offsets the negative effect more for women and those who have used AI" is a key finding from the heterogeneity analysis. Given the potential power issues mentioned in the general comments, this claim should be presented with a degree of caution.

This is a fair point. We have updated the text on page 5 to:

"Note that statistical power is lower in these specifications compared to the primary specifications, so we interpret these with some degree of caution."

Page 19, Section 3.3, Paragraph 4: Regarding the Flesch-Kincaid score, please add a sentence explicitly acknowledging the limitations of applying this US-centric, English-language metric to a diverse, multi-national, non-native English-speaking sample.

Thank you, another reviewer also noted this and we have included updated language on page 13 in the methodology section:

“a limitation is that Flesch-Kinkaid score was developed for American English and thus may contain some measurement error.”

and on page 25 in the discussion section as a limitation:

“the Flesch-Kincaid model was developed for English speakers in the US and may not be as good of a measure in contexts where English is not the first language.”

Page 22, Section 4.2, Paragraph 4: The discussion posits that the highly educated sample "may view accessibility differently, favoring technical language and detail". This is a crucial point and likely a primary driver of the results. <u>This argument should be elevated from an exploratory point to a central theme in the Discussion section.</u> The negative perception of the lower-grade-level AI text may not be a penalty for ambiguity but a correct assessment of its lower value to this specific audience.

We agree with your assessment. We have fleshed out this point in the discussion session, noting that:

“However, the study also has some limitations. First, while the sample is relevant, it is a convenience sample and does not represent all types of users who would find the type of evidence produced relevant; our sample is highly educated and does not include journalists, for example. In fact, the demographics of our sample, particularly high levels of education, is likely the ***most important driver*** *of our result. Consequently, these results are most applicable for researchers who would like to communicate their research to stakeholders who are technically advanced compared to the general public. Our result on “readability" may indicate a preference for more technical language among this group.”*

Page 25, Section 4.2, Paragraph 4: The gender analysis reveals that women "rate truthfully disclosed AI-generated blogs more highly ($p<0.05$)". This is a fascinating result. The manuscript would benefit from a more developed theoretical interpretation of this finding. Why might women value the truthful signal of AI authorship more than men in this context?

This is a very interesting question that we also spent a lot of time thinking through. There is another paper we mention (Aldaroso et al. 2024) that finds gender differences as well. They find that privacy concerns and potential risks explain a quarter of the gap in the gender use of AI. With that in mind, we could conjecture that trust may drive this result. In fact, our data shows that women report trusting AI 10 percentage points less than men do ($p = 0.067$). We

could potentially run a triple interaction with both gender and trust, but given the likelihood of being underpowered, we do not believe that would be a good strategy to address this question. We have included this result in the manuscript on page 20 where we say:

"We conduct a pre-specified analysis given some evidence that gender influences perceptions about AI (Aldasoro 2024) with privacy and potential risks explaining a quarter of the gender gap in AI use."

and

"Note that we find that women are ten percentage points less likely to report trusting AI compared to men (p = 0.067), suggesting that trust may play a role."

A theoretical exploration of this issue is, unfortunately, beyond the scope of this manuscript, but we have included this issue in the discussion section as potential future research and are considering expanding on this question in future work. Thanks for the suggestion!

Recommendations for Additional Citations

Page 10, Introduction, Paragraph 4: To strengthen the discussion on the scientific community grappling with the responsible use of AI, consider including literature that reflects on the broader ethical and practical dilemmas AI poses to core academic processes like peer review.

Ben Saad H, Dergaa I, Ghouili H, Ceylan Hİ, Chamari K, Dhahbi W: The assisted Technology dilemma: a reflection on AI chatbots use and risks while reshaping the peer review process in scientific research. AI & SOCIETY 2025:1-8.

Page 13, Introduction, Paragraph 5: When discussing the main use-cases of AI for economic research, such as idea generation and summarizing text, it would be beneficial to cite a review that illustrates the breadth of AI applications in another specific scientific domain. This would exemplify its role beyond communication and into areas like motion analysis and injury prevention.

[Souaifi M, Dhahbi W, Jebabli N, Ceylan Hİ, Boujabli M, Muntean RI, Dergaa I. Artificial Intelligence in Sports Biomechanics: A Scoping Review on Wearable Technology, Motion Analysis, and Injury Prevention. Bioengineering. 2025; 12(8):887.]

Page 14, Conceptual Framework, Paragraph 4: In the discussion of readers' "cognitive effort and time investment," you could briefly contextualize the importance of cognitive performance by citing an example from another field that demonstrates how even simple physiological interventions can significantly impact cognitive outcomes, reinforcing the value of reducing cognitive load for the reader.

[Bouzouraa E, Dhahbi W, Ferchichi A, Geantă VA, Kunszabo MI, Chtourou H, Souissi N. Single-Night Sleep Extension Enhances Morning Physical and Cognitive Performance Across Time of Day in Physically Active University Students: A Randomized Crossover Study. Life. 2025; 15(8), 1178.]

Page 13, Introduction, Paragraph 4: When discussing the need for responsible use and disclosure of AI, you could support this point by drawing a parallel to the broader scientific need for rigorous validation of any new technology or device before its widespread adoption, citing an example of such a validation study from the sports science literature.

[Ardigò LP, Palermi S, Padulo J, Dhahbi W, Russo L, Linetti S, Cular D, Tomljanovic M: External responsiveness of the SuperOpTM device to assess recovery after exercise: A pilot study. Frontiers in sports and active living 2020, 2:67.]

Page 23, Results, Paragraph 1: In the exploratory analysis where you discuss biases in perceptions about AI, you could draw a brief analogy to other fields where subjective perception is a key driver of behavior, such as how taste perception influences dietary intake, to frame the broader relevance of studying perceptual biases.

Karmous I, Ben Othman R, Dergaa I, Ceylan Hİ, Bey C, Dhahbi W, Sayed KA, Jamoussi H, Ioan MR, Khan NA. Sweet and Fat Taste Perception: Impact on Dietary Intake in Diabetic Pregnant Women—A Cross-Sectional Observational Study. Nutrients. 2025;17(15), 2515.

Thank you for these additional references; they are very helpful in fleshing out the literature. We have included some in the paper. The paper on the use of AI in the peer-review process was especially relevant and interesting to read; we felt it went up front and highlighted it in the introduction. A couple of them are quite specific to other areas, and we prefer to keep the discussion focused on theory and evidence in communication in academic research, so we have not included all of them this time around. We also need to adhere to the word limit imposed by the journal.

Reviewer #2: The manuscript studies how readers judge short research blogs when authorship is either human or AI and when the label shown to readers either tells the truth or misleads. Across an eleven country sample and a preregistered two by two experiment, the authors report that AI generated posts receive slightly lower quality ratings than human written posts, yet this difference disappears when readers are truthfully told that a post was produced by AI. Readability, measured with standard indices, is higher for the AI versions, while stated intentions to engage with the underlying research do not differ across conditions. The question is timely, the design is clean, and the results are easy to apply for scholars who are deciding whether or how to disclose AI assistance.
The study's contribution rests on three solid pillars. First, the authors pose a concrete, policy relevant dilemma that institutions and journals face now, and they frame it with an information and signaling logic that distinguishes ambiguity from honest disclosure. Second, the cross randomization of true authorship and displayed authorship neatly isolates that mechanism while preregistration, IRB approval, and clear outcomes bolster credibility. Third, the authors report heterogeneity by gender and by prior AI use, which invites practical segmentation for science communication. Taken together, these features make the paper readable, transparent, and directly useful for editorial and outreach policy.

Thank you for your kind words!

The most important limitation concerns <u>who the readers are</u>. The sample is highly educated and connected to a policy research network, which is a strength for internal validity but narrows external validity. Such audiences may be unusually tolerant of technical prose, unusually familiar with AI tools, and unusually attentive to disclosure language. I encourage the authors to <u>foreground this boundary condition in the abstract and opening section</u> and to consider a replication on a general audience panel, in undergraduate populations who often consume research explainers, and in non English settings. Even a brief appendix table that compares demographics and AI use to national benchmarks would help readers judge generalizability.

Thank you, this is an important point. As another reviewer pointed out, the fact that the sample is highly educated and connected to policy research could be a main driver of our results. While we did already acknowledge that in the paper, we have now foregrounded this in several parts of the paper. The abstract is now as follows:

"Academic research is not always available in a form that is accessible or engaging to a non-academic audience, hindering readers' engagement with it. Non-academics, even if highly educated and policy experts in their fields, tend to need research to be presented in a more accessible way than peer-reviewed articles --- one example being non-technical

blogs. However, writing these requires effort from researchers. Artificial Intelligence (AI) tools can make academic research easier to understand by summarizing and simplifying academic papers much more quickly than researchers can, making it easier for researchers to produce such summaries. However, disclosure of AI use may lower readers' perceived quality of and trust in the blog, generating a tradeoff for the researcher. In this paper, we evaluate an 11-country experiment cross-randomizing a blog's actual and reported author as AI or human. We find that research stakeholders rate the quality of AI-generated blogs marginally lower than human-written ones ($p < 0.1$), but disclosure of AI use offsets the negative effect ($p < 0.1$). The sample in this study, while being the policy relevant sample who would engage with such policy-oriented academic research, is one that is highly educated and includes some thematic specialists. Indeed, findings indicate that their view of "accessibility" of research is such that they prefer slightly more technical summaries of research. The nature of the respondents may thus explain the particular findings in this study, suggesting that researchers should tailor their prompts for their intended audience. There are no effects on readers' reported likelihood of engaging with the blog or on beliefs about others predicted engagement with it. Consequently, we hypothesize that researchers can leverage AI to communicate their research more easily without a penalty from disclosing its use."

*In the introduction, on page 4 (***NOTE:** the page numbers are those in the *new, clean* version of the paper, not the tracked changes version)*, we say:*

"we implemented a four-arm factorial design with 366 respondents who are relatively highly educated and connected with policy-relevant research (but were not researchers themselves)."

And in the discussion section on page 26, we say:

"while the sample is relevant and lends internal validity, it is a convenience sample and does not represent all types of users who would find the type of evidence produced relevant; our sample is highly educated and connected to research and policy and does not include journalists, for example. In fact, the demographics of our sample, particularly high levels of education, is likely the ***most important driver*** *of our result. Consequently, these results are most applicable for researchers who would like to communicate their research to stakeholders who are technically advanced compared to the general public."*

In terms of generalizability, we actually don't find that our respondents are more aware of AI or are more likely to use it than in general populations in various low-income countries. For bereviety and to adhere to the maximum word count, we do not include a table. However, a

2025 KPMG report notes the below, that we have added in the results section where we describe the characteristics of the sample on page 15:

"For context, 80% of people in low-income countries use AI tools on a regular or semi-regular basis, compared to 58% in high-income countries. Thus, our sample's use of AI is lower than that of the general population in low-income countries."

A second limitation lies in the outcomes. The paper relies on Likert ratings and self reported intentions to read, share, or follow up, which are informative but do not capture behavior. If server side tracking is not feasible, the authors could add a short follow on task with incentive compatible choices, such as a chance to click through to the underlying article, to bookmark, or to trade earnings for more information. Even a simple measure of time on page or a binary click through outcome would substantively strengthen the claim that disclosure does not reduce engagement.

Thank you for this wonderful idea. It would have been great to integrate this into our experiment. We do intend to conduct further experiments in this space, so we could certainly integrate actual actions rather than simply self-reported intentions in the future. It was unfortunately not possible to have click throughs this time. However, we do have a measure of the amount of time the reader spent reading the blog and we find that there are no statistically significant differences on this outcome between the different experimental groups (this is reported in Appendix Tables A.23 and A.24). For brevity, we put them in the Appendix but would be happy to put them into the main text if preferred. We have also included this as a limitation for the study on page 26:

"The outcomes are also self-reported, potentially rendering them over- or under-rated."

and as a suggestion in the directions for future research in the discussion section on page 26:

"Future work could also incorporate actual behaviour rather than self-reported intended behaviour. For example, providing the opportunity for respondents to click through to the underlying paper or to the authors' websites, and tracking this behaviour."

The presentation of stimuli and the theorized pathway could be elaborated. Readers need more detail on how the AI and human texts were generated and standardized: model and version, prompts, temperature, length constraints, topic balance, and any editing rules. Readability checks are helpful but do not exhaust style markers that shape perceived credibility. Simple text analytics on sentence length variance or lexical diversity would

reassure that the two sources produced comparable prose. On the theory side, the signaling account could be tied more tightly to the observed heterogeneity. A compact path diagram that distinguishes disclosure effects that operate through expectations from those that operate through perceived clarity would clarify what the experiment identifies. Finally, with N spread across four arms and many countries, please report minimum detectable effects, country variance, and results with country fixed effects or clustering to calibrate precision.

This is an important point. Appendix C displays all of the prompts that were used, which were very detailed and prescriptive to ensure that the blogs were consistently generated. We have added the model version (ChatGPT-4) in all instances in which we mention it.

In terms of sentence length variance or lexical diversity, we actually did not want them to necessarily produce comparable texts. We wanted to see whether these differences mattered across the experimental groups. We checked, and sentences are longer in human-written blogs (by 7 words, on average) and the number of paragraphs is larger in human-written blogs (by 1.2 paragraphs, on average). However, human-written blogs have more sentences per paragraph. So, the complexity does not go all in one direction. We have also noted on page 18:

AI-generated blogs have "fewer words per sentence (20 vs. 27), and fewer paragraphs (12.6 vs. 13.8), though more sentences per paragraph (3.6 vs. 2.2)."

Your point on heterogeneity and potentially insufficient power is an important one. In fact, ex-ante, we did not think we would have the power to detect heterogeneous effects. We do so as exploratory analysis and we state on page 18 that:

"We first note that given our relatively small sample size, statistical power could be limited. Ex-post power calculations suggest that we are reasonably powered in the quality outcomes, and thus, to the extent that we may be sufficiently powered to detect some differential effects, we attempt such an analysis."

And on page 5 we additionally note:

"Note that statistical power is lower in these specifications compared to the primary specifications, so we interpret these with some degree of caution."

So, while we conduct heterogeneity analysis, we interpret these results as exploratory. Given that this analysis is secondary and exploratory (though interesting), further development of the theory may not contribute as much to the paper, especially given the mandated word limit of the journal.

The following are our MDEs for our three main outcomes (assuming power of 0.8, p 0.05, means and standard deviations of the sample, and 366 observations divided across 8 groups (to account for the interaction of 4 groups plus the variable used for heterogeneity analysis):

Quality:

- *Unweighted average 10 attributes: 0.40 points (on the scale of 0-4)*
- *Unweighted average 9 attributes: 0.41 points*
- *Weighted average 10 attributes: 0.41 points*

Engagement:

- *Engagement PCA score: 0.59 standard deviations*
- *Engagement unweighted average (0-4): 1.56 points*
- *Engagement sum (0-5): 0.98 points*

Others' engagement:

- *Others' engagement PCA score: 0.59 standard deviations*
- *Others' engagement unweighted average (0-4): 0.49 points*
- *Others' engagement sum (0-5): 1.1 points*

We are thus reasonably powered for the quality outcomes, but less so for the engagement and others' engagement outcomes. Consequently, we note your point that this is still exploratory and have added details about power in the methods section.

Note that our regression specification does include country fixed effects (these are absorbed into the strata fixed effects since randomization strata included countries, gender, and seniority). Additionally, our standard errors are clustered at the individual level since randomization assignment was at the individual level (so they are heteroskedasticity-robust).

The table below shows the variance within countries for each of our primary outcomes.

	Quality			Own engagement			Others' engagement		
Country	Unweighted Average Quality (0-4) (10 Attributes)	Weighted Average Quality (0-4) (9 Attributes)	Unweighted Average Quality (0-4) (9 Attributes)	Standardized First Principal Component	Average engagement rating (0-4)	Number of likely or very likely statements (0-5)	Standardized First Principal Component	Average Engagement Rating (0-4)	Number of likely or very likely statements (0-5)
**Bangladesh**	0.53	0.56	0.56	0.97	0.77	2.93	0.94	0.68	3.56
**Egypt**	0.21	0.2	0.2	0.65	0.44	1.58	0.96	0.64	2.81
**Ethiopia**	0.33	0.36	0.36	0.72	0.51	2.26	0.96	0.66	3.36
**Ghana**	0.57	0.6	0.6	1.17	0.88	2.99	1.03	0.73	3.46
**India**	0.36	0.37	0.38	0.94	0.69	3.02	0.88	0.65	3.41
**Kenya**	0.23	0.25	0.24	0.66	0.42	1.88	0.83	0.53	3
**Malawi**	0.52	0.55	0.55	1.13	0.85	2.65	1.22	0.9	3.99
**Nigeria**	0.39	0.43	0.44	0.91	0.67	2.56	0.88	0.54	3.69
**Rwanda**	1.48	1.33	1.44	2.69	1.99	4.67	1.94	1.43	4.62
**Sudan**	0.29	0.31	0.3	0.3	0.2	1.78	0.63	0.37	2.19
**Uganda**	0.35	0.45	0.36	1.18	0.8	3.19	2.24	1.53	6.5

Transparency and compliance details are mostly in order, but one item needs attention for this journal. Data and code should be available at publication. The paper currently states that data will be provided upon acceptance. Please deposit de identified data, code, survey instrument, preregistration link, and all blog stimuli in a trusted repository and update the data availability statement. With those improvements, my overall view is positive. I recommend revise and resubmit at the level of minor to moderate changes. The practical message will be valuable to editors, communicators, and researchers alike: one can use AI to produce accessible research summaries and, provided disclosure is honest and clear, expect no meaningful penalty in perceived quality or stated engagement.

We have made the data and replication files available on the IFPRI Dataverse: https://doi.org/10.7910/DVN/GETDGX

To deepen your framing of audience priors, trust, and disclosure effects, please engage with recent higher education research on generative AI adoption from Palestinian and

regional contexts. These studies clarify why perceptions differ by prior AI use and offer constructs you can mobilize (performance expectancy, effort expectancy, social influence, facilitating conditions, habit, anxiety). They will also broaden the geographic scope of your literature review and sharpen your policy implications.

1. Ayyoub, A., Khlaif, Z. N., Hamamra, B., Bensalem, E., Mitwally, M., Sanmugam, M., ... & Khaldi, M. (2025). Drivers of acceptance of generative AI through the lens of the extended Unified Theory of Acceptance and Use of Technology. Human Behavior and Emerging Technologies, 2025(1), 6265087.
2. Hamamra, B., Khlaif, Z. N., & Mayaleh, A. (2025). Between promise and precarity: Palestinian university educators' experiences with AI integration in higher education. Interactive Learning Environments, 1–16.
3. Hamamra, B., Khlaif, Z. N., Mayaleh, A., & Baker, A. A. (2025). A phenomenological examination of educators' experiences with AI integration in Palestinian higher education. Cogent Education, 12(1), 2526435.
4. Khlaif, Z. N., Hamamra, B., Bensalem, E., Mitwally, M. A., & Sanmugam, M. (2025). Factors influencing educators' technostress while using generative AI: A qualitative study. Technology, Knowledge and Learning, 1–26.

These sources provide theory backed constructs for your mechanism section, empirics from a Global South context to strengthen external validity claims, and concrete variables you could measure in a follow up (acceptance, anxiety, technostress) to connect disclosure to downstream behavior. I recommend citing them in the related work when introducing audience priors and in the discussion when articulating policy and practice implications for research communication and higher education.

Thank you for pointing us to these important studies. The paper on technostress was especially interesting. We have cited these in the paper and there are several measures that will be useful to emulate in future research. Thank you!

Reviewer #3: I would recommend acknowledging the limitations of the study and a stronger argumentation concerning further studies. Otherwise I consider the manuscript to be well written, presented in a logical and convincing manner, in good style and transparent with respect to the adopted methodology. I particularly highlight the fact that the study covers a solid number of countries and was conducted in four waves, ensuring the representativeness of the data.

Thank you for these kind words! We have included a discussion section that now includes limitations of the study and future directions for research, which has enriched the paper.

---

6. PLOS authors have the option to publish the peer review history of their article (what does this mean?). If published, this will include your full peer review and any attached files.

If you choose “no”, your identity will remain anonymous but your review may still be made public.

Do you want your identity to be public for this peer review? For information about this choice, including consent withdrawal, please see our Privacy Policy.

Reviewer #1: **Yes:** Wissem Dhahbi

Reviewer #2: No

Reviewer #3: **Yes:** Cernicova-Buca Mariana

[NOTE: If reviewer comments were submitted as an attachment file, they will be attached to this email and accessible via the submission site. Please log into your account, locate the manuscript record, and check for the action link "View Attachments". If this link does not appear, there are no attachment files.]

While revising your submission, please upload your figure files to the Preflight Analysis and Conversion Engine (PACE) digital diagnostic tool, https://pacev2.apexcovantage.com/. PACE helps ensure that figures meet PLOS requirements. To use PACE, you must first register as a user. Registration is free. Then, login and navigate to the UPLOAD tab, where you will find detailed instructions on how to use the tool. If you encounter any issues or have any questions when using PACE, please email PLOS at figures@plos.org. Please note that Supporting Information files do not need this step.
