# Supplementary material for: Man vs. machine: Multi-country experimental evidence on the quality and perceptions of AI-generated research blog content
Source: PLoS One. 2026 Mar 25;21(3):e0342852. doi: 10.1371/journal.pone.0342852 (PMC13016282; doi:10.1371/journal.pone.0342852)
Supplement: Attachment — Submitted filename: PONE review blogs vs texts.docx [file pone.0342852.s004.docx]

I read the manuscript “Man vs. Machine: Global experimental evidence on the quality and perceptions of AI generated research content” with great interest, since it deals with the perceived quality of professional content, evaluated by educated readers, in a very specific context. The analysis is thorough and timely, since AI vs human-generated topic represents a highly debated topic both in scientific and in general articles.

The authors set the background in a legitimate manner, engaging with relevant literature, which is presented in a clear and convincing style. The structure of the manuscript is logical and presented in a transparent manner, early in the article and followed through up to the end.

The data obtained in the experiment is presented fairly and sustains the proposed theme of the article. Personally, I would have liked a corpus having scientific articles accompanied by blogs authored by the same (groups of) researchers, as one sample, and scientific texts accompanied by AI generated blogs as another, but I understand that the logic behind the experiment dictated a different approach. Also, I would have liked to see in the survey a stronger discussion on the engagement of readers with the content (blogs and original research articles that the blogs summarize or popularize). However, the authors of the manuscript explained their endeavors quite legitimately and it can be accepted as such. I particularly signal the fact that the experiment was carried out across a significant number of countries and in four waves, factors that ensure a representativeness of data.

An aspect that I would suggest for strengthening the manuscript is for the authors to acknowledge the limitations of their study and to better announce the possibility of further studies and implications of their findings.

The figures in the manuscript are sufficient and help understanding the data. Also the appendices are useful, containing valuable information, while not overloading the manuscript with elements that could hinder the flow of the article.

In conclusion, I believe that the manuscript is of publishable quality, with minor revisions that can be easily carried out in the preparation for final editing.
