# Supplementary material for: Man vs. machine: Multi-country experimental evidence on the quality and perceptions of AI-generated research blog content
Source: PLoS One. 2026 Mar 25;21(3):e0342852. doi: 10.1371/journal.pone.0342852 (PMC13016282; doi:10.1371/journal.pone.0342852)
Supplement: Attachment — Submitted filename: response_to_reviewers_20260112.docx [file pone.0342852.s006.docx]

Dear Dr. Keenan,

Thank you for submitting your manuscript to PLOS ONE. After careful consideration, we feel that it has merit but does not fully meet PLOS ONE’s publication criteria as it currently stands. Therefore, we invite you to submit a revised version of the manuscript that addresses the points raised during the review process.

Please submit your revised manuscript by Jan 21 2026 11:59PM. If you will need more time than this to complete your revisions, please reply to this message or contact the journal office at [plosone@plos.org](mailto:plosone@plos.org). When you're ready to submit your revision, log on to <https://www.editorialmanager.com/pone/> and select the 'Submissions Needing Revision' folder to locate your manuscript file.

Please include the following items when submitting your revised manuscript:

- A rebuttal letter that responds to each point raised by the academic editor and reviewer(s). You should upload this letter as a separate file labeled 'Response to Reviewers'.
- A marked-up copy of your manuscript that highlights changes made to the original version. You should upload this as a separate file labeled 'Revised Manuscript with Track Changes'.
- An unmarked version of your revised paper without tracked changes. You should upload this as a separate file labeled 'Manuscript'.

If you would like to make changes to your financial disclosure, please include your updated statement in your cover letter. Guidelines for resubmitting your figure files are available below the reviewer comments at the end of this letter.

If applicable, we recommend that you deposit your laboratory protocols in protocols.io to enhance the reproducibility of your results. Protocols.io assigns your protocol its own identifier (DOI) so that it can be cited independently in the future. For instructions see: [https://journals.plos.org/plosone/s/submission-guidelines#loc-laboratory-protocols](https://track.editorialmanager.com/CL0/https:%2F%2Fjournals.plos.org%2Fplosone%2Fs%2Fsubmission-guidelines%23loc-laboratory-protocols/1/010f019afb73ff6d-dda78564-a7d9-47d7-b183-379c953bc013-000000/rdtO82TFPQyRhZBpuG-dI0GE9lU4WC4gbnDF27M_4Fk=240). Additionally, PLOS ONE offers an option for publishing peer-reviewed Lab Protocol articles, which describe protocols hosted on protocols.io. Read more information on sharing protocols at [https://plos.org/protocols?utm_medium=editorial-email&utm_source=authorletters&utm_campaign=protocols](https://track.editorialmanager.com/CL0/https:%2F%2Fplos.org%2Fprotocols%3Futm_medium=editorial-email%26utm_source=authorletters%26utm_campaign=protocols/1/010f019afb73ff6d-dda78564-a7d9-47d7-b183-379c953bc013-000000/1e7nL29VkAHWGBl_5Eytrcbp2eeT9aXPjDGSKXulzX4=240).

We look forward to receiving your revised manuscript.

Kind regards,

Jafar Kolahi
Academic Editor
PLOS One

Journal Requirements:

1. If the reviewer comments include a recommendation to cite specific previously published works, please review and evaluate these publications to determine whether they are relevant and should be cited. There is no requirement to cite these works unless the editor has indicated otherwise. 

2. Please review your reference list to ensure that it is complete and correct. If you have cited papers that have been retracted, please include the rationale for doing so in the manuscript text, or remove these references and replace them with relevant current references. Any changes to the reference list should be mentioned in the rebuttal letter that accompanies your revised manuscript. If you need to cite a retracted article, indicate the article’s retracted status in the References list and also include a citation and full reference for the retraction notice.

We thank you for the opportunity to re-submit the manuscript. We appreciate yours and the reviewers’ comments and have incorporated all of them into the revision. In addition, we made some minor grammatical changes (which will appear in the tracked changes) and updated the citations to all have sentence case titles for consistency. Thank you again, and we look forward to hearing back from you.

Additional Editor Comments:

Change the title to "Man vs. Machine: Multi-Country experimental evidence on the quality and perceptions of AI-generated research blog content"

Thank you – we have revised the title accordingly.

[Note: HTML markup is below. Please do not edit.]

Reviewers' comments:

Reviewer's Responses to Questions

**Comments to the Author**

1. If the authors have adequately addressed your comments raised in a previous round of review and you feel that this manuscript is now acceptable for publication, you may indicate that here to bypass the “Comments to the Author” section, enter your conflict of interest statement in the “Confidential to Editor” section, and submit your "Accept" recommendation.

Reviewer #1: All comments have been addressed

Reviewer #2: All comments have been addressed

Reviewer #3: All comments have been addressed

2. Is the manuscript technically sound, and do the data support the conclusions?

The manuscript must describe a technically sound piece of scientific research with data that supports the conclusions. Experiments must have been conducted rigorously, with appropriate controls, replication, and sample sizes. The conclusions must be drawn appropriately based on the data presented.

Reviewer #1: Partly

Reviewer #2: Yes

Reviewer #3: Yes

3. Has the statistical analysis been performed appropriately and rigorously?

Reviewer #1: Yes

Reviewer #2: Yes

Reviewer #3: (No Response)

4. Have the authors made all data underlying the findings in their manuscript fully available?

The [PLOS Data policy](http://track.editorialmanager.com/CL0/http:%2F%2Fwww.plosone.org%2Fstatic%2Fpolicies.action%23sharing/1/010f019afb73ff6d-dda78564-a7d9-47d7-b183-379c953bc013-000000/iJAYx-P-HEctP5nO5VkCMjea2h6BV-okYYJMjw7zQWM=240) requires authors to make all data underlying the findings described in their manuscript fully available without restriction, with rare exception (please refer to the Data Availability Statement in the manuscript PDF file). The data should be provided as part of the manuscript or its supporting information, or deposited to a public repository. For example, in addition to summary statistics, the data points behind means, medians and variance measures should be available. If there are restrictions on publicly sharing data—e.g. participant privacy or use of data from a third party—those must be specified.

Reviewer #1: Yes

Reviewer #2: Yes

Reviewer #3: Yes

5. Is the manuscript presented in an intelligible fashion and written in standard English?

PLOS ONE does not copyedit accepted manuscripts, so the language in submitted articles must be clear, correct, and unambiguous. Any typographical or grammatical errors should be corrected at revision, so please note any specific errors here.

Reviewer #1: Yes

Reviewer #2: Yes

Reviewer #3: Yes

6. Review Comments to the Author

Please use the space provided to explain your answers to the questions above. You may also include additional comments for the author, including concerns about dual publication, research ethics, or publication ethics. (Please upload your review as an attachment if it exceeds 20,000 characters)

Reviewer #1: General Comments

The authors have substantially improved the manuscript in this revision. They have adequately addressed the major concerns regarding sample generalizability and the interpretation of "accessibility" within a highly educated demographic. The introduction of a dedicated "Discussion" section significantly strengthens the paper by placing the findings in the correct context and acknowledging the limitations of the Flesch-Kincaid metric for non-native English speakers. The statistical approach (OLS) has been justified satisfactorily.

Major Weaknesses: There are no remaining major weaknesses. The authors have successfully reframed the study to reflect that the results apply to a specific, policy-relevant, highly educated audience rather than the general public.

Minor Weaknesses: The primary remaining issues are editorial and formatting in nature.

Citation Formatting: There are numerous instances where internal reference codes (e.g., "Noy2023 [3]") remain in the text alongside the bracketed numbers. These must be cleaned.

There must be some confusion with the citations, as these issues are not showing up in the version without tracked changes. In any case, we have gone through to ensure that the citations are not showing up with the author-year code and are in-line with the journal requirements.

Sentence Structure: Several sentences in the added text (Abstract and Discussion) are slightly convoluted and require syntactic polishing for clarity.

We have addressed these below where you have kindly provided us with specific instances.

Typos: There are minor typographical errors introduced during the revision process.

We have also addressed these below where you have provided us with specific instances.

Specific Comments

Abstract

Page 1, Line 119-120: The sentence "The sample in this study, while being the policy relevant sample who would engage with such policy-oriented academic research..." is wordy.

Edit: Simplify to: "The study sample consists of policy-relevant stakeholders who typically engage with academic research; they are highly educated and include thematic specialists."

Thank you – we have changed the sentence to what you have suggested.

Page 1, Line 122: "findings indicate that their view of 'accessibility' of research is such that..."

Edit: Clarify to: "findings indicate that this audience interprets 'accessibility' differently, preferring..."

Thank you – changed.

Introduction

Page 4, Line 6: The citation format "Arnautu2021 [1]" contains the author/year key.

Edit: Ensure all in-text citations follow the journal style (e.g., just "[1]" or "Arnautu and Dagenais [1]"). This error repeats throughout the manuscript (e.g., Line 9 "Korinek2023 [2]", Line 13 "Noy2023 [3]").

We have ensured that these instances appear as they should, not including the author-year code.

Page 4, Line 14: "Jiao2023" appears without brackets.

Please see above comments on citations.

Page 4, Line 15: The sentence starting "In addition, some studies highlight..." is a good addition regarding non-native speakers, but ensure the citation "[5]" is placed correctly.

This sentence had been removed in the revised version.

Page 4, Line 28: The phrasing "The seminal paper by [16] and a more recent paper... by [17]" is grammatically awkward.

Edit: Change to "The seminal paper by Akerlof [16] and a more recent paper by Gans [17]..."

Thank you. We changed it to: “The seminal paper by Akerlof [15] and a more recent paper by Gans [16], specifically related to communication, provide useful frameworks by which to think about such communication and the introduction and perceptions of AI.”

Methods

Page 13, Line 406: "While developed for U.S. school grades a limitation is that it was developed for American English..."

Edit: This sentence is grammatically broken due to the insertion of the limitation clause. Rephrase: "A limitation of this metric is that it was developed for American English and U.S. school grades; thus, it may contain measurement error when applied to this context."

Thank you – fixed.

Results

Page 17, Line 487: The phrase "using the adjusted p-values" is used.

Clarification: Ensure consistency in terminology between "adjusted p-values" and "RI p-values" established in the methods.

Thank you – changed to RI p-values for consistency.

Page 22, Line 544: "This result could point to a behavioural bias against AI as this unfamiliarity increases ambiguity."

Comment: This is a logical inference and well-placed.

Thank you – no changes were made.

Page 23, Line 568: The sentence "We conduct a pre-specified analysis given some evidence that gender influences perceptions about AI Aldasoro2024 [59] with privacy..." contains a formatting error with the citation key and runs on.

Edit: Remove "Aldasoro2024" and fix punctuation.

Please see other comments on the citations. We added a comma after [59], but the issues with the citations are fixed overall.

Discussion

Page 26, Line 663: "In fact, the demographics of our sample... is likely the most important driver of our result."

Comment: This is a crucial acknowledgment that strengthens the paper's validity.

Thank you – no changes made.

Page 26, Line 667: "An additional limitation is that our measure of 'readability'. We note that..."

Edit: Fragmented sentence. Change to: "An additional limitation is our measure of 'readability'."
Thank you – fixed.

Page 26, Line 670: "...rendering them over- or under-stated."

Edit: "stated" should likely be "estimated" or keep "stated" if referring to the reporting itself, but "under-reported" is more standard.

Thank you – we changed it to ‘under-reported’.

Reviewer #2: The authors have addressed the comments fully. I recommend the publication of this timely, pressing article.

Thank you.

Reviewer #3: I read the revised manuscript with great interest and I feel that my initial comments received satisfactory solutions. I gained readability and fluence, reads well and responds to debates in the scientific community. However, there are minor issues that should be addressed.

1. In the current form, both in the Discussions section and in Conclusions, the authors present the idea that using AI in research is welcomed, desired, having merit etc. especially for academic blogging. Using a more careful language here is a must: depends on the culture or expectations of audiences and on other factors. While readers/audiences are more receptive (or less critical, depends on the point of view) when AI use is disclosed, there are sufficient examples when 100% human authored content is considered of premium value. Assessing that AI use is desirable seems more like a technophilic wishful thinking, rather than an established truth.

Thank you for pointing this out. We have toned down the language to indicate that AI can be beneficial in certain situations and *on average* (i.e., not necessarily for every reader). The first four paragraphs of the discussion are now written as follows:

*The main implication of this paper is that academics and other technical writers can credibly use AI to convey their research in a more accessible way, under certain conditions. In this study, since neither the actual nor reported author substantially affects respondents’ engagement and \emph{if} a disclosure statement is provided, AI-generated communications are not viewed as lower quality. AI may be a viable alternative for researchers when writing blogs for technical audiences, since it reduces their cost of effort without compromising perceptions of quality or any potential engagement, on average. However, there may be cases where the audience is less receptive to AI or where a premium is placed on human-generated content.*

*We innovate by using a randomized experiment comparing the actual and reported authorship of research blogs (AI vs. Human) whereby respondents from eleven countries rated a blog's quality. AI-generated content was rated lower in quality than human-written content. However, when an AI-disclosure statement was included, respondents did not penalize AI-generated material, highlighting the importance of truthful signaling of authorship in research communication. Engagement with, and others' predicted engagement with the blogs were not affected by the true or reported author.*

*That the negative perception of quality of AI-generated blogs is offset by truthful disclosure suggests that the respondents in this sample slightly penalize ambiguity and that the advantage of eliminating ambiguity outweighs the penalty of exposing the potentially lower effort involved in using AI. Consequently, researchers can benefit, on average, from the reduced cost of effort of using AI in research blogs without incurring the penalty in perceptions of quality of signaling low effort \emph{provided} they include a disclosure statement.*

*That this sample's preferred blog length exceeded the actual length suggests that preferences regarding accessibility depend on the audience. Results by the grade level of the blog suggest that a lower reading level may also signal weaker research quality, as academic language often conveys rigor. Researchers should thus tailor their AI prompts, and/or the decision to use AI, to their audiences accordingly.*

2. Following my comment above, I wonder why authors left the idea of ethical challenges hanging. The debate around disclosing AI use and using it at all has strong roots in research ethics, as shown by the authors in the literature review and in referring to ethical approval for their survey. However later in the Discussions or Conclusions this idea is no longer present.

This is a good addition to the discussion section. We have added the following as the third-to-last paragraph:

“Beyond its role in reducing informational ambiguity, disclosure of AI use also relates to ongoing debates in research ethics. While our experiment was not designed to evaluate ethical norms, the findings suggest an important alignment: transparent disclosure does not appear to carry a perceived quality or engagement penalty in this context and may mitigate negative perceptions associated with ambiguity. At the same time, our results do not shed light on broader ethical concerns surrounding AI use in research, including authorship credit, responsibility for errors, or the preservation of scholarly norms.”

3. Minor linguistic verification of the manuscript is necessary. There are some sentences which need clarification. For instance "An additional limitation is that our measure of “readability”." This sentence is unfinished. Or, earlier, authors state that "This study has several strengths", but only two are highlighted, which cannot be considered several.

Thank you for pointing this out. We changed ‘several strengths’ to ‘two key strengths’. We have also gone through the manuscript and ensured grammatical issues are fixed.

7. PLOS authors have the option to publish the peer review history of their article ([what does this mean?](https://track.editorialmanager.com/CL0/https:%2F%2Fjournals.plos.org%2Fplosone%2Fs%2Feditorial-and-peer-review-process%23loc-peer-review-history/1/010f019afb73ff6d-dda78564-a7d9-47d7-b183-379c953bc013-000000/aW3XWuRReqEiH7eLgSMeqqjvGaOCbFaNRz-87480G5w=240)). If published, this will include your full peer review and any attached files.


If you choose “no”, your identity will remain anonymous but your review may still be made public.


**Do you want your identity to be public for this peer review?** For information about this choice, including consent withdrawal, please see our [Privacy Policy](https://track.editorialmanager.com/CL0/https:%2F%2Fwww.plos.org%2Fprivacy-policy/1/010f019afb73ff6d-dda78564-a7d9-47d7-b183-379c953bc013-000000/Uqx3eUGbd2hTb_M20ozQrq_YT2vwJ08GiAI_R43MEh8=240).

Reviewer #1: **Yes:** Wissem Dhahbi

Reviewer #2: No

Reviewer #3: **Yes:** Assoc. Prof. Mariana Cernicova-Buca

[NOTE: If reviewer comments were submitted as an attachment file, they will be attached to this email and accessible via the submission site. Please log into your account, locate the manuscript record, and check for the action link "View Attachments". If this link does not appear, there are no attachment files.]

To ensure your figures meet our technical requirements, please review our figure guidelines: <https://journals.plos.org/plosone/s/figures>

You may also use PLOS’s free figure tool, NAAS, to help you prepare publication quality figures: <https://journals.plos.org/plosone/s/figures#loc-tools-for-figure-preparation>.

NAAS will assess whether your figures meet our technical requirements by comparing each figure against our figure specifications.
